# Plasma apolipopotein C-2 elevation is associated with Takayasu arteritis

**DOI:** 10.1038/s41598-021-98615-3

**Published:** 2021-09-23

**Authors:** Natsuko Tamura, Yasuhiro Maejima, Yuka Shiheido-Watanabe, Shun Nakagama, Mitsuaki Isobe, Tetsuo Sasano

**Affiliations:** 1 Department of Cardiovascular Medicine, Tokyo Medical and Dental University, 1-5-45 Yushima, Bunkyo-ku, Tokyo, 113-8519 Japan; 2 Sakakibara Heart Institute, Japan Research Promotion Society for Cardiovascular Diseases, Tokyo, Japan

**Keywords:** Diagnostic markers, Aortic diseases

## Abstract

Takayasu arteritis (TAK) is an autoimmune systemic arteritis of unknown etiology. Although a number of investigators have attempted to determine biomarkers for diagnosing TAK, there exist no specific serological markers of this intractable disease. We undertook the exploration of novel serological markers which could be useful for an accurate diagnosis of TAK using an unbiased proteomics approach. The purified plasma samples from untreated patients with TAK and healthy individuals were separated by two-dimensional electrophoresis. The differentially expressed protein spots were detected by gel comparison and identified using matrix-assisted laser desorption/ionization time-of-flight/time-of-flight mass spectrometry (MS). Next, we validated plasma concentrations of identified proteins by enzyme-linked immunosorbent assay (ELISA). Two-dimensional electrophoresis and numerical analysis revealed 19 spots and 3 spot clusters whose sum of the sample averages was ≥ 0.01, and the average concentrations were ≥ 1.5 times in the patient group compared with the control group. Among them, 10 spots and spot clusters that met the condition of the average spot concentration being 2.5 times more than that in the control group were selected. After processing these spots using MS and conducting MS/MS ion search, we identified 10 proteins: apolipoprotein C-2 (ApoC-2), actin, apolipoprotein A-1, complement C3, kininogen-1, vitronectin, α2-macroglobulin, 14–3–3 protein ζ/δ, complement C4, and inter-α-trypsin inhibitor heavy chain H4 isoform 1 precursor. Finally, ELISA demonstrated that plasma ApoC-2 level was significantly elevated in patients with TAK compared with that in healthy individuals. Thus, ApoC-2 would be a promising candidate biomarker for TAK diagnosis.

## Introduction

Takayasu arteritis (TAK) is an idiopathic systemic arteritis affecting large arteries, including the aorta and its major branches. Although the overall mortality rate of patients with TAK is not high, TAK occasionally causes critical complications, including heart failure due to aortic regurgitation, coronary artery stenosis, or myocarditis^1^. TAK is more prevalent in females than in males and in Asians than in other races, with an average age of onset of 25 years^2^. Thus, epidemiological studies have suggested that genetic factors may play a role. However, the precise etiology of TAK remains unresolved.

An early diagnosis of TAK is extremely difficult because of the nonspecific symptomatic disease presentation and the absence of specific biomarkers as well as the rarity of the disease. To resolve such a diagnostic difficulty associated with TAK, a number of investigators have attempted to identify specific biomarkers for easy diagnosis of TAK. We and several other groups have identified potential serological markers for TAK, including pentraxin-3^3,4^, serum amyloid A^5^, interleukin (IL)-6^6^, IL-12^7^, IL-18^8^, matrix metalloproteinases^9^, soluble intercellular adhesion molecule 1^10^, and soluble receptor for advanced glycation end products^11^. However, as the levels of these serological markers could elevate in response to various inflammatory diseases, they have not been used for the practical diagnosis and/or monitoring of relapse of TAK^12^. Indeed, there are no biomarkers more sensitive than serum C-reactive protein (CRP) for monitoring TAK^13^.

Based on this background, we performed comprehensive proteome analyses, and subsequent validation using enzyme-linked immunosorbent assay (ELISA), from blood samples of patients with TAK to determine biomarkers specifically for the diagnosis of TAK.

## Results

### Two-dimensional electrophoretic analysis

**Figure 1:**
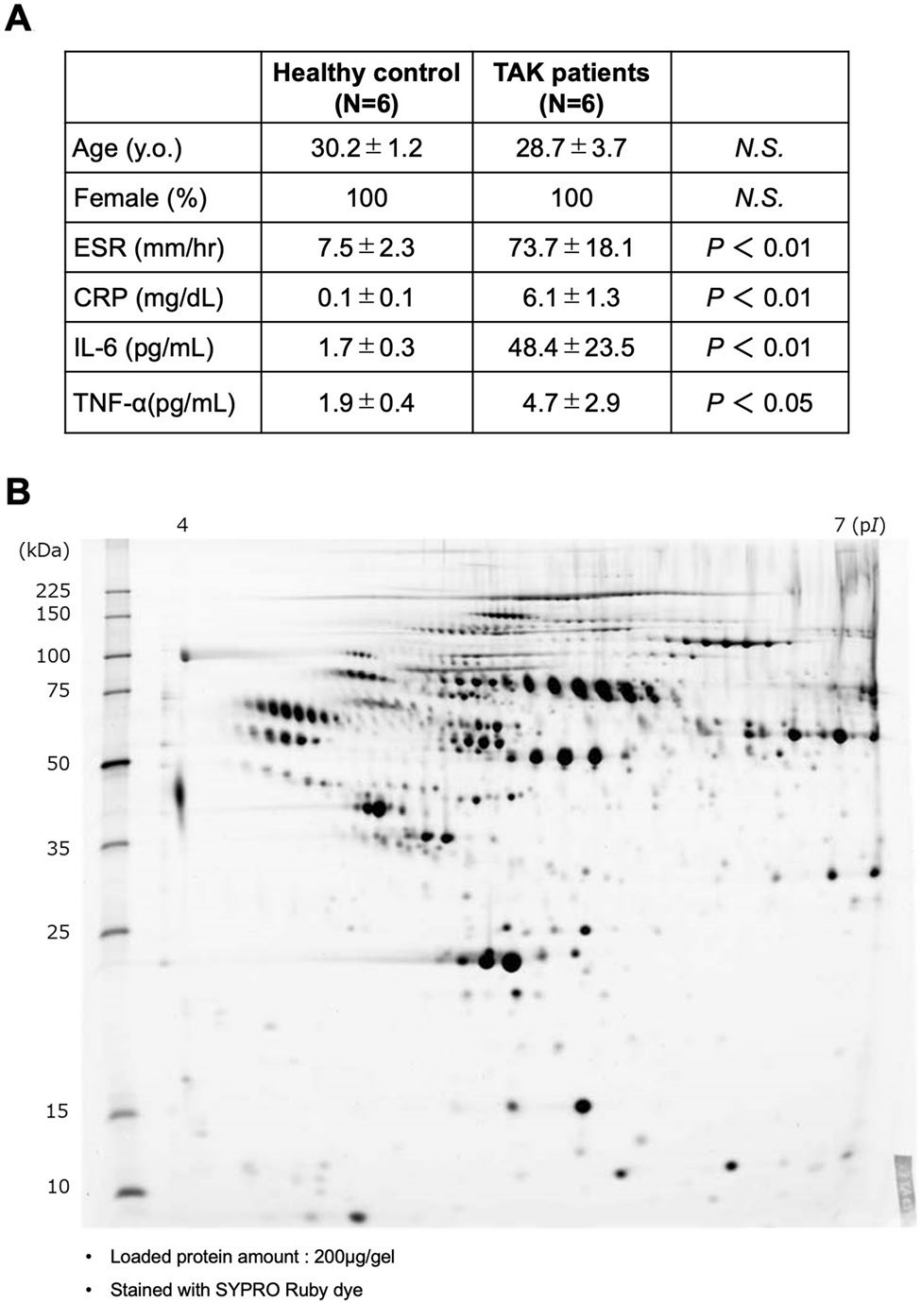
(**A**) The characteristics of subjects whose plasma samples were analyzed by two-dimensional electrophoresis in this study. (**B**) A representative image of the gels [Takayasu arteritis (TAK) patient group: sample #1] stained with a fluorescent stain (SYPRO Ruby Protein) for detecting the total protein content. Protein contaminants were removed from the plasma samples of both groups (patients with TAK, N = 6; healthy control subjects, N = 6), and the purified samples were separated by two-dimensional electrophoresis.

**Figure 2:**
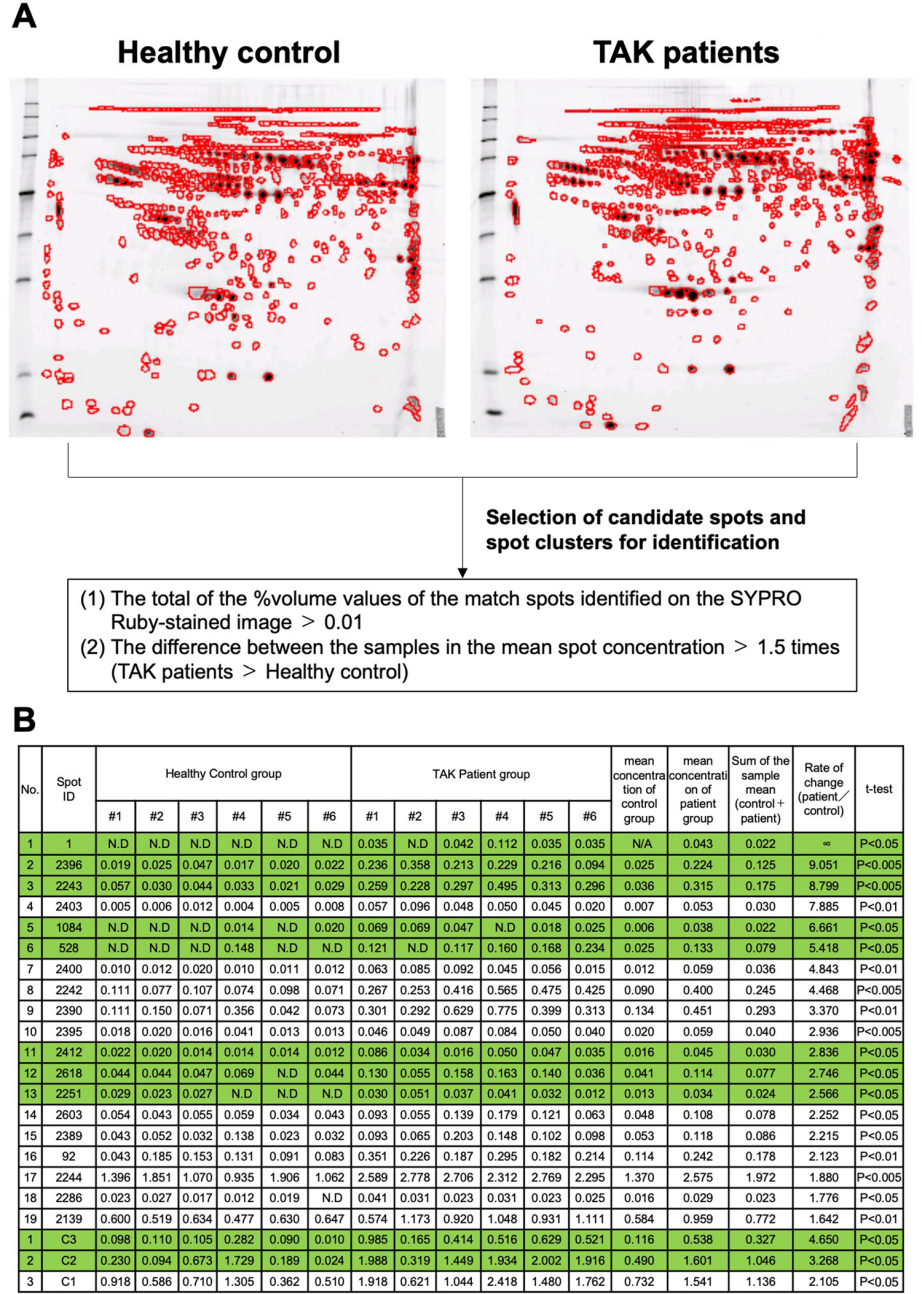
(**A**) Representative gel images visualized using a fluorescent scanner and protein spots detected using Image Master 2D Platinum software. *Left:* healthy control group. *Right:* Takayasu arteritis (TAK) patient group. (**B**) Numerical analysis of the obtained fluorescently stained spots after two-dimensional electrophoresis and digitization. There are 19 spots and 3 spot clusters whose sum of the sample means is 0.01 or greater and the mean spot concentration is 1.5 times or higher (*P* < 0.05) in the TAK patient group compared with the control group. Among them, 10 spots and spot clusters that meet the condition of mean spot concentration of 2.5 times or higher (*P* < 0.05) in the TAK patient group compared with the control group are selected (ID: 1, 2396, 2243, 1084, 528, 2412, 2618, 2251, C3, and C2, highlighted in green).

The characteristics of subjects whose plasma samples were analyzed by two-dimensional electrophoresis are shown in Fig. 1A. The mean ages of the patient (N = 6) and control (N = 6) groups were 28.7 and 30.2 years, respectively. Both groups only consisted of females. The disease status of all six patients was pre-pulseless. The value of erythrocyte sedimentation rate, and levels of CRP, IL-6, and tumor necrosis factor alpha in the plasma were markedly higher in patients with TAK than in healthy individuals. After extracting major proteins, the purified plasma samples obtained from the study subjects were separated by two-dimensional electrophoresis, and each gel was stained with a fluorescent stain (SYPRO Ruby Protein) for detecting total protein levels (Fig. 1B, Supplementary Fig. 3A). Next, the images of these gels were visualized using a fluorescent scanner, with the protein spots detected using Image Master 2D Platinum software (Fig. 2A, Supplementary Figs. 1A,B/3B,C). Numerical analysis of the obtained fluorescent spots was performed. We selected spots and spot clusters that met the following criteria: (1) the total % volume values of the match spots on the SYPRO Ruby-stained image (sum of the sample averages) was ≥ 0.01, and (2) the difference in the mean spot concentration between the samples was ≥ 1.5 fold. In the control group, 50 spots met these criteria (*P* < 0.05, data not shown). Contrastingly, in the patient group, 19 spots and 3 spot clusters met these criteria (*P* < 0.05, Fig. 2B, Supplementary Fig. 2A–F). Among these 19 spots and 3 spot clusters, ten spots and spot clusters that met the condition of the mean spot concentration, 2.5 fold more than that in the control group (*P* < 0.05, Fig. 2B) were selected (ID: 1, 2396, 2243, 1084, 528, 2412, 2618, 2251, C3, and C2). As the components of spots in #2403, #2400, #2242, #2390 and #2395 were regarded as identical with that of #2396, these spots were not selected. Similarly, as the component of spots in #2242 was regarded as identical with that of #2243, this spot was not selected.

### MS/MS analysis

**Figure 3:**
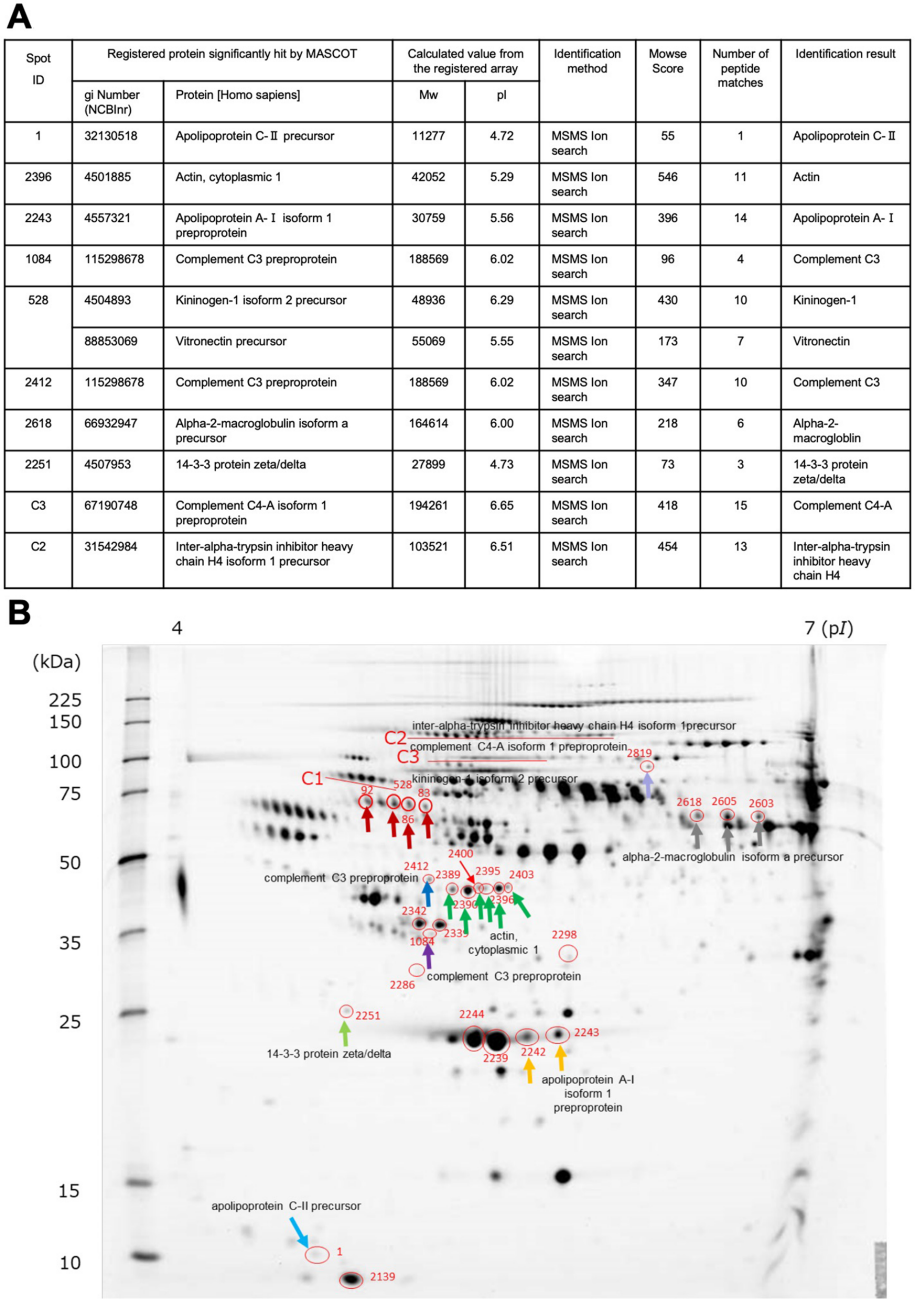
(**A**) Gel pieces containing ten spots and spot clusters, having mean concentration of 2.5 times or higher (*P* < 0.05) in the Takayasu arteritis (TAK) patient group compared with the healthy control group, were cut out, and the peptides were extracted and measured using matrix-assisted laser desorption/ionization time-of-flight/ time-of-flight mass spectrometry (Ultrafle Xtreme, Bruker Daltonics, Billerica, MA, USA). Indicated table shows the measured values for the registered sequences, Mowse scores, number of peptide matches and identification results. (**B**) A representative image of the two-dimensional electrophoresis and fluorescence staining using a patient plasma sample (Takayasu arteritis (TAK) patient group: sample #5). Red circles indicate protein spots and spot clusters whose sums of the means are 1.5 times or higher in the TAK patient group compared with that in the control group. The arrows indicate spot and spot cluster names of the proteins detected by mass spectrometry.

The gel pieces containing the ten spots and spot clusters were cut out, and the peptides were extracted and measured using matrix-assisted laser desorption/ionization time-of-flight/ time-of-flight mass spectrometry (MALDI-TOF/TOF MS) (UltrafleXtreme) (Supplementary Fig. 4A–J). Two proteins were detected in sample no. 528 and the same proteins (complement C3) were detected in sample no. 1084 and 2412; therefore, 10 proteins were searched by MS/MS ion search from the ten spots and spot clusters. Figure 3A lists the registered proteins that received significant hits on MASCOT, measured values from the registered sequences, Mowse scores, number of peptide matches, and identification results. The identified proteins were apolipoprotein C2 (ApoC-2), actin, apolipoprotein A1 (ApoA-1), complement C3, kininogen-1, vitronectin, α2-macroglobulin, 14–3–3 protein ζ/δ, complement C4, and ITIH4.

The spots and spot clusters with proteins identified by MS/MS ion search are shown on a two-dimensional gel in Fig. 3B. Red circles indicate spots and spot clusters with mean concentrations more than 1.5 times in the patient group compared with the control group, and the arrow indicates the identified protein.

### ELISA

**Figure 4:**
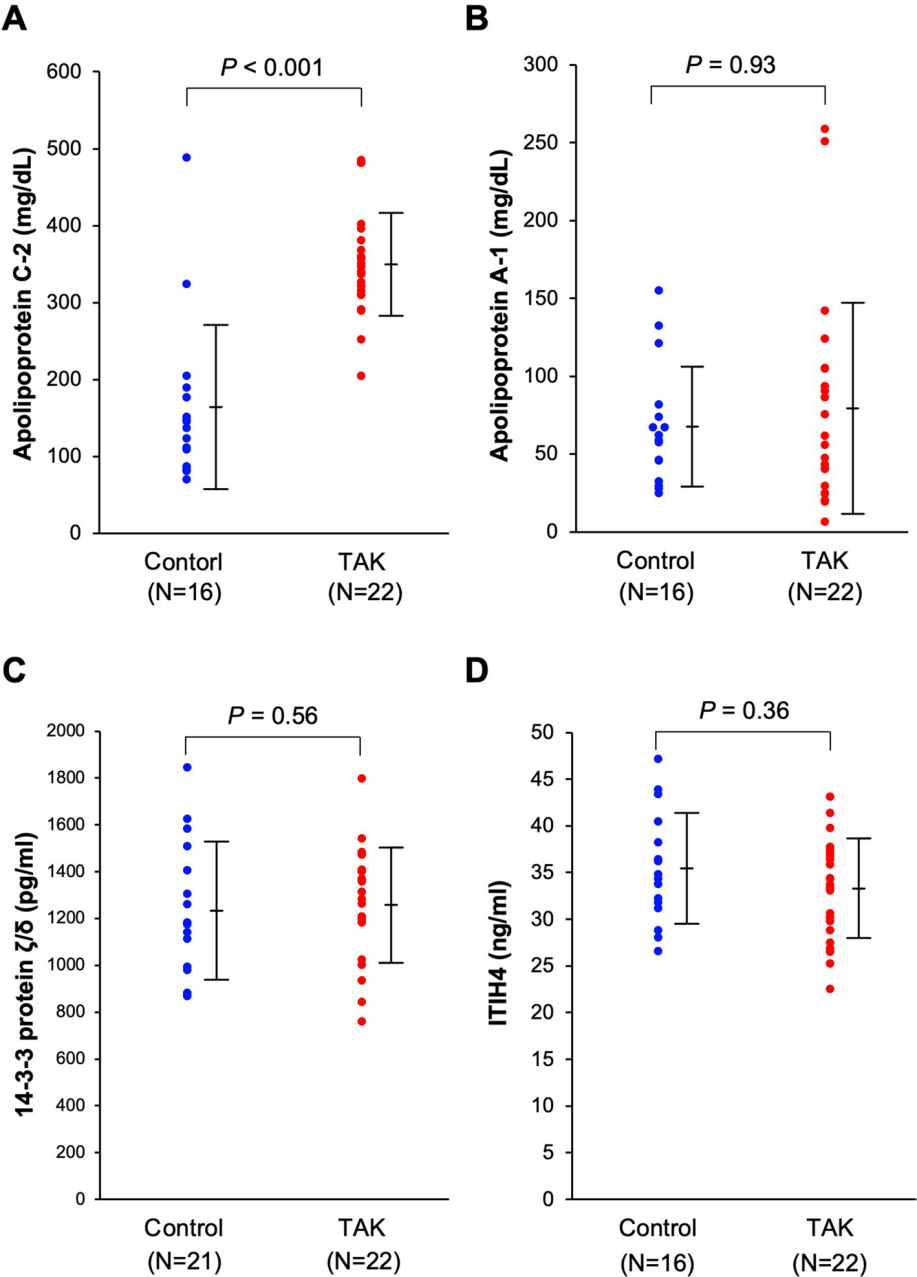
(**A**) The plasma level of apolipoprotein C2 (ApoC-2) is significantly increased in the Takayasu arteritis (TAK) patient group compared with that in the control group (control: N = 16, mean = 82.1 mg/dL; TAK: N = 22, mean = 173.6 mg/dL; *P* < 0.001). The clinical standard level of ApoC-2 in the plasma is 1.5–3.8 mg/dL for women. (**B**) The plasma level of apolipoprotein A1 (ApoA-1) shows no significant difference between the TAK patient and control groups (control: N = 16, mean = 67.6 mg/dL; TAK: N = 22, mean = 79.2 mg/dL; *P* = 0.93). (**C**)The plasma level of 14–3–3 protein ζ/δ shows no significant difference between the TAK patient and control groups (control: N = 16, mean = 1233.6 pg/mL; TAK: N = 22, mean = 1259.3 pg/mL; *P* = 0.53). (**D**) The plasma level of inter-α-trypsin inhibitor heavy chain H4 isoform 1 precursor (ITIH4) shows no significant difference between the TAK patient and control groups (control: N = 16, mean = 35.4 ng/mL; TAK: N = 22, mean = 33.6 ng/mL; *P* = 0.50).

Before conducting ELISA for validating plasma concentrations of identified proteins by proteome analyses, we made a short list of biomarker candidates using the 10 identified proteins. Among them, complement C3, complement C4, kininogen-1, vitronectin, and α2-macroglobulin levels were not measured by ELISA, because it is widely known that levels of nonspecific inflammatory markers, such as CRP, erythrocyte sedimentation rate, and IL-6, are elevated in patients with TAK. Similarly, actin level was not measured because it is widely used as a protein control in quantitative analyses. We increased the number of samples (control, N = 16, patient, N = 22) and measured the plasma protein levels of ApoC-2, ApoA-1, 14–3–3 protein ζ/δ, and ITIH4 by ELISA. Medical background of the 22 patients is shown in Supplementary Fig. 5. All were female, mean age at blood collection was 42.0 years, mean age of onset was 26.0 years, and mean duration of illness was 16.0 years. According to the Numano classification, there were 6 patients with type 1, 3 patients with type 2a, 5 patients with type 2b, 0 patients with type 3, 1 patient with type 4, and 7 patients with type 5. The level of plasma ApoC-2 was significantly higher in the TAK patient group than that in the control group (control: N = 16, mean = 82.1 mg/dL; TAK: N = 22, mean = 173.6 mg/dL; *P* < 0.001; Fig. 4A). The clinical standard level of ApoC-2 in the plasma is 11.8–4.6 mg/dL (for women; SRL Company, Tokyo, Japan). Contrastingly, although the plasma levels of ApoA-1 tended to increase in the TAK patient group compared with the control group, the difference was not significant (control: N = 16, mean = 67.6 mg/dL; TAK: N = 22, mean = 79.2 mg/dL; *P* = 0.93; Fig. 4B). Similarly, the plasma levels of both 14–3–3 protein ζ/δ and ITIH4 showed no significant differences between the TAK patient and control groups (14–3-3 protein ζ/δ: control: N = 16, mean = 1233.6 pg/mL; TAK: N = 22, mean = 1259.3 pg/mL; *P* = 0.53; Fig. 4C/ITIH4: control: N = 16, mean = 35.4 ng/mL; TAK: N = 22, mean = 33.6 ng/mL; *P* = 0.50; Fig. 4D).

Taken together, these results suggested that the plasma level of ApoC-2 was elevated with a significant clinical difference in patients with TAK.

## Discussion

Here, we conducted the comprehensive proteomic analysis of plasma samples obtained from patients with TAK. Two-dimensional electrophoresis and MS revealed ten proteins with increased concentrations in the plasma of patients with TAK before treatment, compared with that in the plasma of healthy individuals. Finally, we validated that ApoC-2 level was significantly elevated in the plasma of patients, compared with that in the plasma of healthy individuals through ELISA.

ApoC-2 is present on chylomicrons and in very low-density lipoproteins and high-density lipoproteins, and activates the lipid transport system as a catalyst for the core triglycerides of circulating chylomicrons and very low-density lipoprotein^14^. A number of epidemiological studies suggested that there is a significant association between ApoC-2 and cardiovascular diseases^15^. Middelberg RP et al. demonstrated that the gene cluster of Tomm40/ApoE/ApoC-1/ApoC-2/ApoC4 is a marker of cardiovascular risk as well as inflammation by genome wide association study^16^. Recently, various patho-physiological roles of ApoC-2, other than that in lipid metabolism, have been discovered. Zhang et al. demonstrated that ApoC-2 mRNA expression is markedly up-regulated in patients with acute myeloid leukemia, and ApoC-2 promotes the growth of leukemic cells by interacting with CD36, thereby enhancing Lyn-mediated ERK signaling^17^. Similarly, Medeiros et al. reported that ApoC-2 physically interacts with CD36, thereby activating the Lyn-ERK signaling pathway^18^. While little is known about the role of ApoC-2 in autoimmune diseases, the role of its receptor CD36 is more established. The transmembrane CD36 glycoprotein plays a preventive role in autoimmunity by scavenging modified self-antigens, such as apoptotic cells and oxidized low-density lipoprotein in macrophages^19^. Grajchen et al. recently demonstrated that CD36 plays a crucial role in eliminating myelin debris and suppressing neuro-inflammation, thereby alleviating demyelinating disorders, such as multiple sclerosis^20^. Yagi et al. reported the possible association between TAK and CD36 based on their observation of four unrelated patients with TAK who presented CD36 deficiency^21^. These previous observations caused us to hypothesize that the impairment of CD36 expression could be associated with the onset and/or progression of TAK, which resulted in the compensatory elevation of expression of its ligand, ApoC-2, in the plasma. Consistently, our data demonstrated that the plasma ApoC-2 level was significantly elevated in patients with TAK compared with that in healthy individuals, regardless of the disease severity and/or therapeutic status. However, it remains unknown whether the plasma ApoC-2 level is inversely correlated with the expression level or function of CD36 in patients with TAK, thus additional examinations need to be conducted.

ApoA-1, a major component of high-density lipoprotein cholesterol, critically regulates lipid metabolism and acts as an anti-inflammatory and antioxidative modulator, thereby playing a protective role in the cardiovascular system^22^. As it is also known that the presence of auto-antibodies against ApoA-1 is closely associated with disease activity or incidence of cardiovascular events in patients with autoimmune diseases, such as systemic lupus erythematosus, antiphospholipid syndrome, and rheumatoid arthritis^23,24^, we expected the plasma level of ApoA-1 to be a novel biomarker of TAK. The 14–3–3 protein ζ/δ is known to be involved in the homeostatic regulation of T cell trafficking by B cells in autoimmune and chronic inflammatory diseases^25^. As the dysregulation of T cell activity is involved in the etiology of TAK^26,27^, we expected the plasma level of 14–3–3 protein ζ/δ to be a novel biomarker of TAK. ITIH4 is an acute-phase plasma protein produced in the liver in response to the IL-6-mediated proinflammatory stimulation^28^. As TAK disease activity is closely associated with IL-6 expression^29^, we expected the plasma level of ITIH4 to be a novel biomarker of TAK as well. Although the increased levels of ApoA-1, 14–3–3 protein ζ/δ, and ITIH4 were observed in two-dimensional electrophoresis and the following MS/MS analyses in this study, we could not observe a significant elevation of ApoA-1,14–3–3 protein ζ/δ, and ITIH4 levels in the plasma of patients with TAK, as determined by ELISA. Such discrepancy between the proteomic data and ELISA could be caused by the randomness of the characteristics of the ELISA samples. Unlike the samples of the untreated patients with TAK used in the two-dimensional electrophoresis and MS/MS analyses, the plasma samples for ELISA were collected from patients with TAK in various clinical settings, regardless of drug therapy. Thus, to clarify the usefulness of ApoA-1, 14–3–3 protein ζ/δ, and/or ITIH4 as biomarkers of TAK, we need to re-evaluate the plasma concentrations of these molecules in samples from patients with TAK derived from the same conditions in the future.

There are three other limitations to this study. First, we could not compare plasma levels of ApoC-2, ApoA-1, 14–3–3 protein ζ/δ, and ITIH4 in patients with TAK with those in patients with other inflammatory diseases, such as systemic lupus erythematosus, rheumatoid arthritis, coronary artery diseases, and other bacterial infectious diseases. Second, the number of patients with TAK for validating the plasma levels of ApoC-2, ApoA-1, 14–3–3 protein ζ/δ, and ITIH4 was relatively small. Third, we could not examine the usefulness of ApoC-2 for monitoring the disease activity of TAK. Specifically, we did not evaluate the correlation between ApoC-2 and disease activity of TAK or compare the plasma ApoC-2 level before and after initiation of therapy for TAK. Thus, we aim to conduct validation analyses for the current findings through a nationwide large-scale prospective clinical study on patients with TAK.

## Conclusion

In summary, our unbiased proteomics approach demonstrated that ApoC-2 could be a novel sensitive biomarker for use in diagnosis of TAK. We believe that our findings could motivate other investigators to explore the pathogenesis of TAK, especially the lipid-mediated, inflammation-associated mechanism.

## Materials and methods

### Human subjects and ethics declaration

Twenty-eight Japanese patients with TAK who visited University Hospital of Tokyo Medical and Dental University and twenty-two healthy individuals were investigated. Patients were diagnosed with TAK according to the American College of Rheumatology 1990 criteria for the classification of TAK^30^, and their samples were collected before the start of treatment. This research conformed to the ethical guidelines of the 1975 Declaration of Helsinki and was approved by the Ethics Committee of the Tokyo Medical and Dental University for Medical Experiments (Permission number: G2000-180-02), and informed consent was obtained from all participants.

### Sample preparation

Blood samples obtained from six patients with TAK who visited the university hospital of Tokyo Medical and Dental University and six healthy individuals were analyzed using two-dimensional electrophoresis. Venous blood samples were centrifuged at 3000 rpm for 5 min at room temperature. Plasma samples were stored in microtubes at – 20 °C until the assays were performed.

### Two-dimensional electrophoresis

#### Electrophoresis protocol

Plasma samples were processed using a Multiple Affinity Removal Spin Cartridge for Human Serum (Agilent, Santa Clara, CA, USA) to remove protein contaminants and added to a buffer for two-dimensional electrophoresis. Pharmalyte (Merck KGaA, Darmstadt, Germany) was added to the prepared sample to a final concentration of 2%, followed by a protein lysis solution (6 M urea, 2 M thiourea, 2% CHAPTERS, 1% Triton X-100, 1% DTT), adjusted to 0.34 mL. The sample was placed in a swelling tray and covered with the Immobiline DryStrip (Merck KGaA, Darmstadt, Germany). PlusOne DryStrip Cover Fluid was poured over the DryStrip and left overnight. The swollen Immobiline DryStrip was set in the Multiphor II Electrophoresis Unit (Merck KGaA, Darmstadt, Germany) and run at 500 V for 1 min, 3500 V for 8 h at 20 °C. After electrophoresis, the samples were shaken with equilibration buffer A solution (6 M urea, 32% glycerol, 10% SDS, 0.25% DTT, 50 mM Tris–HCl, pH 6.8) for 30 min to equilibrate the solution and set in the DryStrip equilibration tray. Another buffer solution (6 M urea, 32% glycerol, 10% SDS, 4.5% iodoacetamide, 0.125% bromophenol blue, 50 mM Tris–HCl, pH 6.8) was added and the mixture was shaken for 20 min. An acrylamide gel having a concentration gradient of 10–20% was prepared with the DALT Gradient maker (Merck KGaA, Darmstadt, Germany), using a light solution (0.23% bis-acrylamide, 8.77% acrylamide, 0.1% SDS, 0.036% APS, 0.0069% TEMED, 0.375 M Tris–HCl, pH 8.8) and a dense solution (0.46% bis-acrylamide, 17.54% acrylamide, 0.1% SDS, 0.018% APS, 0.001% TEMED, 0.375 M Tris–HCl, pH 8.8). The internal size of the gel was 21 cm × 20 cm, and the thickness was 1 mm. To polymerize the acrylamide completely, the mixture was left to stand for 24 h. The equilibrated Immobiline DryStrip was loaded on the acrylamide gel and fixed with a 1% agarose solution containing 0.125% bromophenol blue. A molecular weight marker was added to the left end of the gel. Electrophoresis was performed using the DALT Multiple Electrophoresis Unit (Merck KGaA, Darmstadt, Germany) at 80 V for 18 h, until a bromophenol blue band was visible at the bottom of the gel. The electrophoresis gel was identified using the serial number printed on the strip in the first dimension isoelectric focusing. The second dimension SDS polyacrylamide gel was identified using the serial number written on a piece of paper enclosed in the gel.

#### Staining protein spots

After electrophoresis, the gel was stained with a fluorescent stain (SYPRO Ruby protein gel stain, S21900, Thermo Fisher Scientific Inc., Waltham, MA, U.S.A.) to detect the total protein content and visualized using a fluorescent scanner with a 488 nm excitation wavelength and 640 nm band pass fluorescence filter at 100 µm resolution.

### Numerical analysis

#### Calculation of the spot signal concentration

A TIFF image file showing fluorescently stained spots was imported into ImageMaster 2D Platinum software (Merck KGaA, Darmstadt, Germany) and digitized. In the numerical analysis, spot detection was performed for each gel, and the spot concentration was measured. Numerical correction between gels was performed by dividing the signal concentration of the spot by the sum of the signal concentrations of all spots on the gel (% volume value). The volume value was expressed as a percentage, and the minimum presentation was 0.001%.

#### Correction processing

After automatic spot detection and matching of identical spots by the software, errors in recognizing match spots and spots with low roundness were corrected.

### Selection of candidate spots and spot clusters for protein identification

When spots and spot clusters corresponded to the following two conditions, they were listed as protein identification candidates. (1) The total of the % volume values of the match spots identified on the SYPRO Ruby-stained image (sum of the sample means) was 0.01 or more. (2) The difference between the samples in the mean spot concentration was 1.5 times or more.

### Tandem mass spectrometry (MS/MS) analysis

Spots stained by silver staining were cut out in 1 mm squares, and 100 μL of 15 mM potassium ferricyanide and 50 mM sodium thiosulfate were added to the gel and shaken for 10 min. After discarding the solution, ultrapure water was added, and the gel pieces were washed until the color from the pieces was removed. To dehydrate the gels, acetonitrile was added. The gels were expanded by adding 10 µL of an oxygen solution of 100 mM ammonium bicarbonate and 0.01 µg/µL trypsin and placed at 37 °C for 16 h. 50 µL each of 0.1% TFA and 50% acetonitrile were added and the gel pieces were shaken for 20 min to extract the peptides. The extraction was repeated twice. The peptide extract was concentrated in a vacuum centrifuge until its volume was approximately 10 µL. After adsorption of the sample on Zip Tip C18 (ZTC18S960, Merck KGaA, Darmstadt, Germany), the peptide was eluted with 2.5 μL of 60% acetonitrile and 0.1% TFA solution. 1 μL of the sample solution and 1 μL of the matrix solution were mixed. The sample was placed on a measurement plate of a mass spectrometer, dried, and then analyzed.

The measurement was conducted using the following procedure. Analysis equipment: Ultrafle Xtreme (Bruker Daltonics, Billerica, MA, U.S.A.), target plate: MTP Anchorchip 600/384 (209513, Bruker Daltonics, Billerica, MA, U.S.A.), polarity: positive mode, detection mode: reflector mode (300–6000 m/z), matrix solution: 0.3 g/L CHCA, 33% acetone, and 66% ethanol, identification software: Mascot (Matrix Science, Boston, MA, U.S.A.), and database: NCBI Refseq human.

MS/MS ion search was performed as follows. Search conditions: human, enzyme: trypsin, fixation modification: carbamide methylation, variable modification: methionine oxidation, peptide mass tolerance: ± 0.1 Da, fragment mass tolerance: ± 0.5 Da, and maximum number of missed cleavages: 1.

### ELISA

We validated plasma concentrations of the identified proteins through ELISA (TAK: n = 22; control (CONT): N = 16). Plasma samples of 22 patients with TAK who visited our university hospital and 16 healthy individuals were examined by ELISA. The following ELISA kits were used: human ApoA-1Quantikine ELISA kit (DAPA10, R&D Systems, Minneapolis, MN, USA), human ApoC-2 ELISA kit (EHAPOC2, Thermo Fisher Scientific Inc., Waltham, MA, USA), human PLA2G2A/SPLA2 ELISA kit (LS-F27815, LifeSpan BioSciences, Seattle, WA, USA), and human inter-α-trypsin inhibitor heavy chain H4 isoform 1 precursor (ITIH4) ELISA kit (LS-F13281-1, LifeSpan Biosciences, Seattle, WA, USA). The assays were performed according to the manufacturers’ instructions.

### Statistical analysis

Data are presented as the mean ± standard deviation. All statistical analyses were performed using IBM SPSS Statistics for Windows, Version 24.0. (IBM Corp., Armonk, NY, USA). A *t*-test was used as a statistical method. Prior to the *t*-test, an F-test was performed to determine whether both sample groups had equal variance. If the variances were equal, a *t*-test was performed using the ordinary method. If the groups had unequal variances, a *t*-test was performed using the Welch method. The spot density “N.D” was treated as zero. *P* < 0.05 denoted a significant difference.

## Supplementary Information

Supplementary Information.

## Data Availability

The data supporting findings of this study are available from the corresponding author upon reasonable request.

## Abbreviations

TAK: Takayasu arteritis
HLA: Human leukocyte antigen
IL: Interleukin
CRP: C-reactive protein
ELISA: Enzyme-linked immunosorbent assay
MS: Mass spectrometry
Apo: Apolipoprotein
ITIH4: Inter-alpha-trypsin inhibitor heavy chain 4
CD: Cluster of differentiation
