# Supplementary material for: Plasma apolipopotein C-2 elevation is associated with Takayasu arteritis
Source: Sci Rep. 2021 Sep 23;11:18958. doi: 10.1038/s41598-021-98615-3 (PMC8460645; doi:10.1038/s41598-021-98615-3)
Supplement: Supplementary file 1 — Supplementary Information. [file 41598_2021_98615_MOESM1_ESM.pdf]

Supplementary Figure 1A

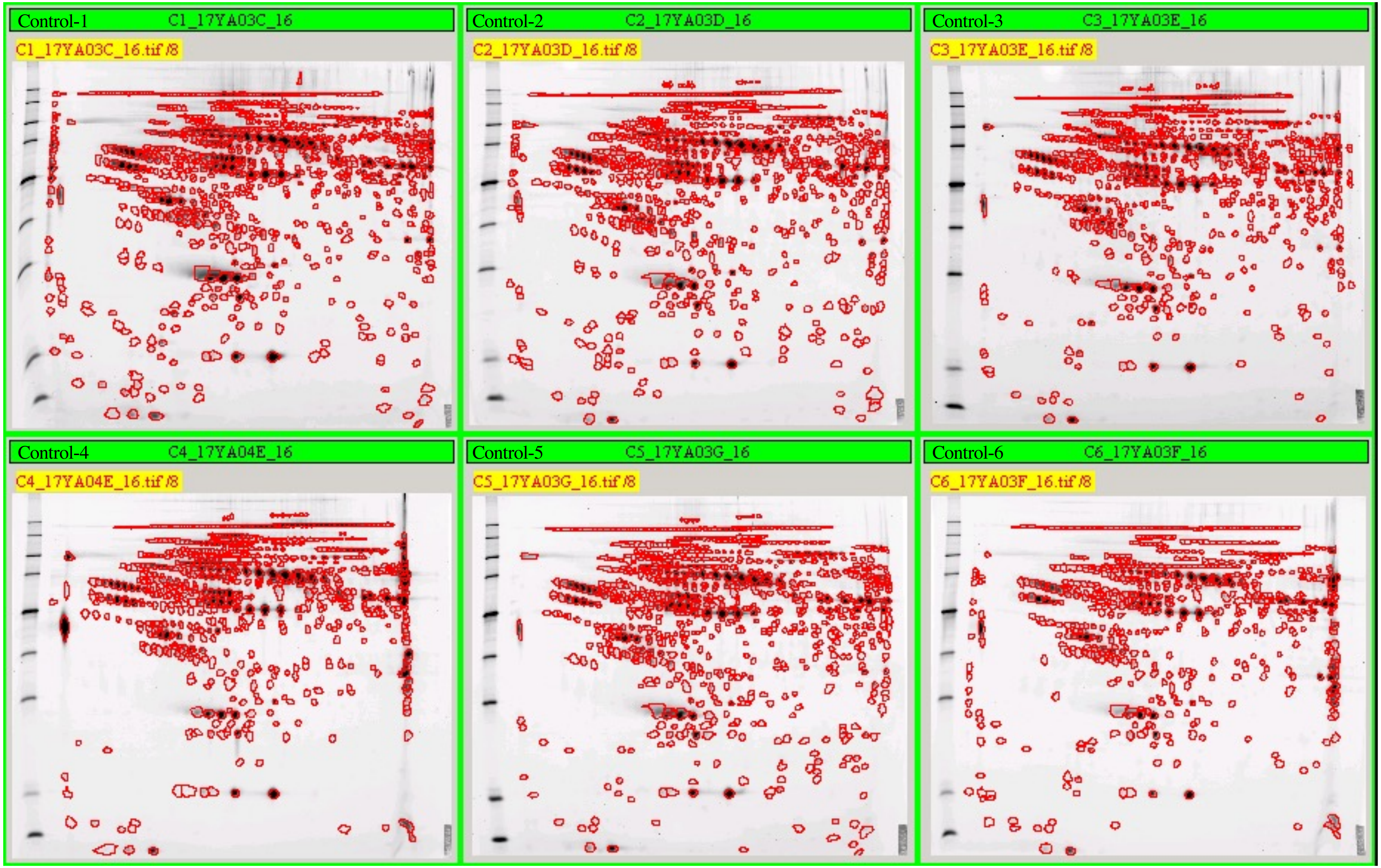

Full gel images visualized using a fluorescent scanner and protein spots detected using Image Master 2D Platinum software (Healthy control group; N=6).

Supplementary Figure 1B

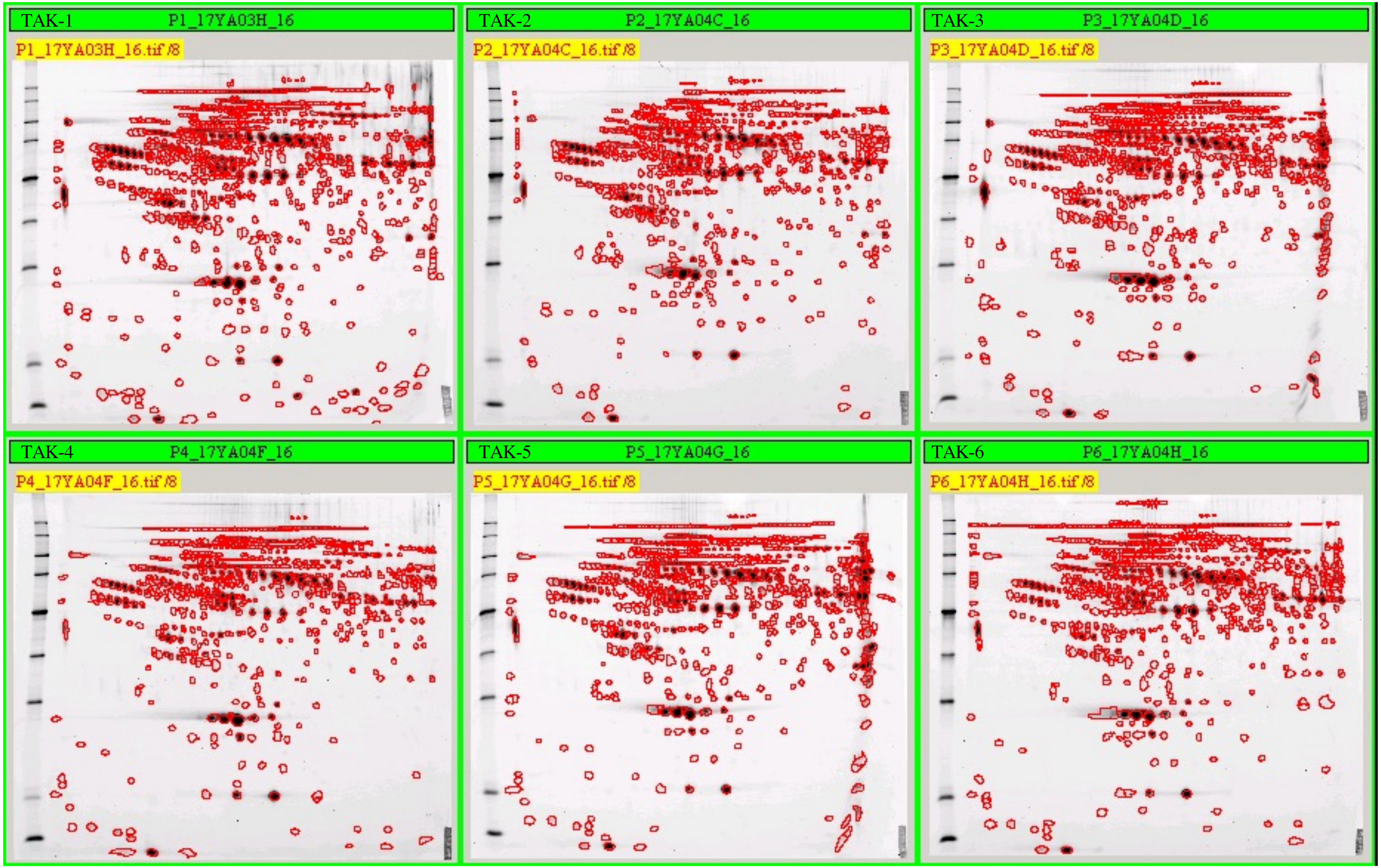

Full gel images visualized using a fluorescent scanner and protein spots detected using Image Master 2D Platinum software (TAK patient group; N=6).

Supplementary Figure 2A

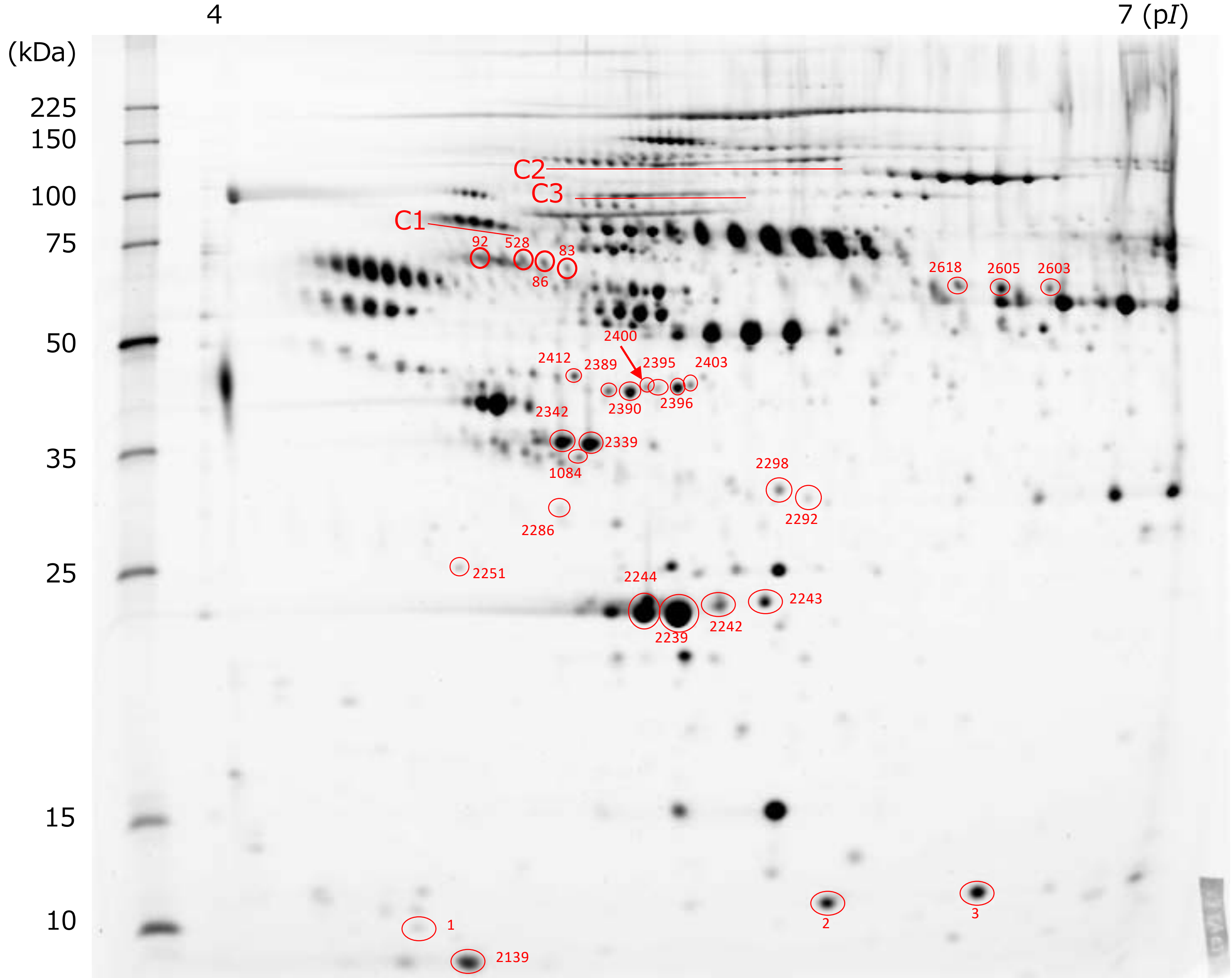

A gel image of 2D-electrophoresis stained with a SYPRO Ruby dye (TAK patient-1). Red circles indicate protein spots and spot clusters that were 1.5 times or more in the TAK patient group compared with that in the control group.

Supplementary Figure 2B

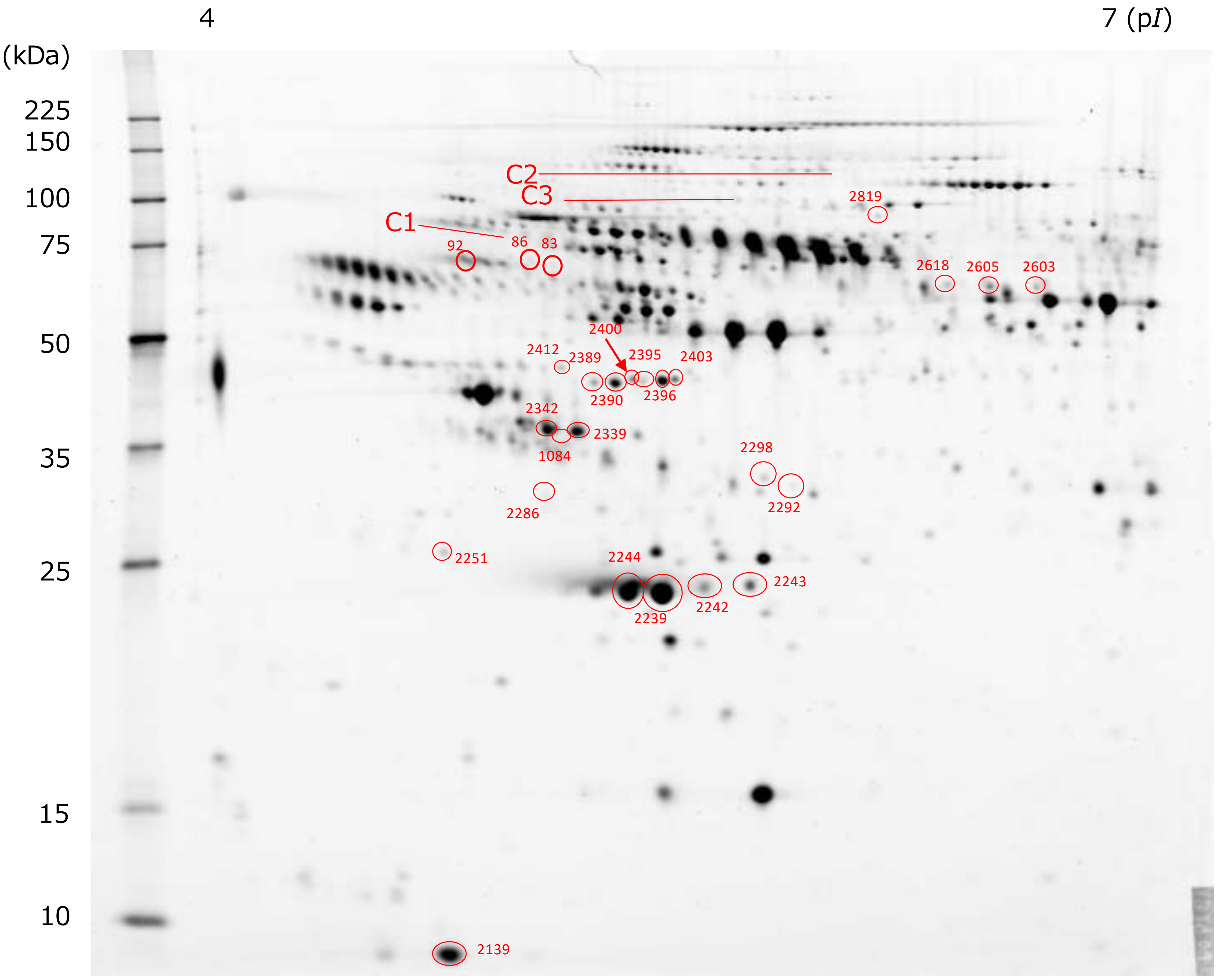

A gel image of 2D-electrophoresis stained with a SYPRO Ruby dye (TAK patient-2). Red circles indicate protein spots and spot clusters that were 1.5 times or more in the TAK patient group compared with that in the control group.

Supplementary Figure 2C

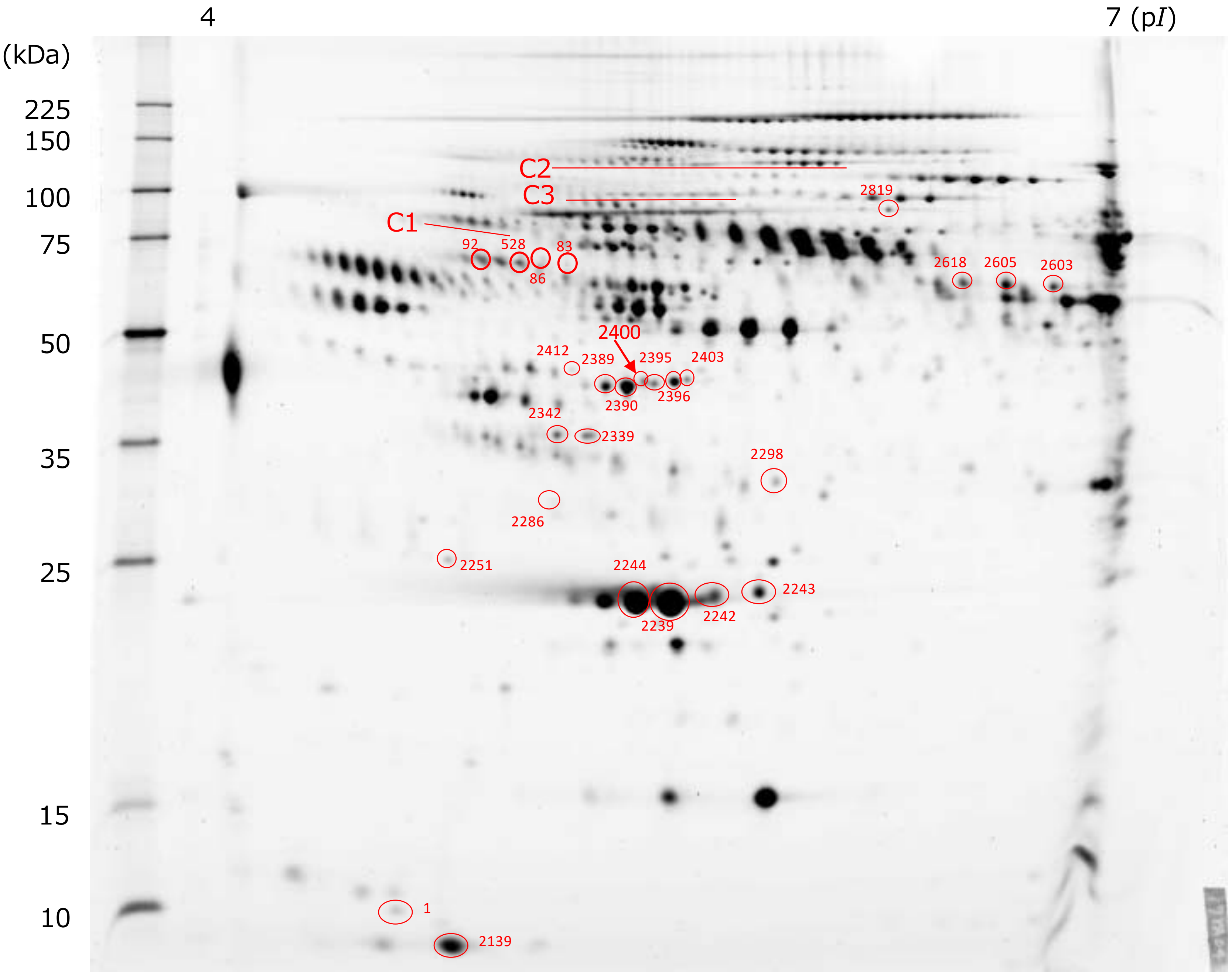

A gel image of 2D-electrophoresis stained with a SYPRO Ruby dye (TAK patient-3). Red circles indicate protein spots and spot clusters that were 1.5 times or more in the TAK patient group compared with that in the control group.

Supplementary Figure 2D

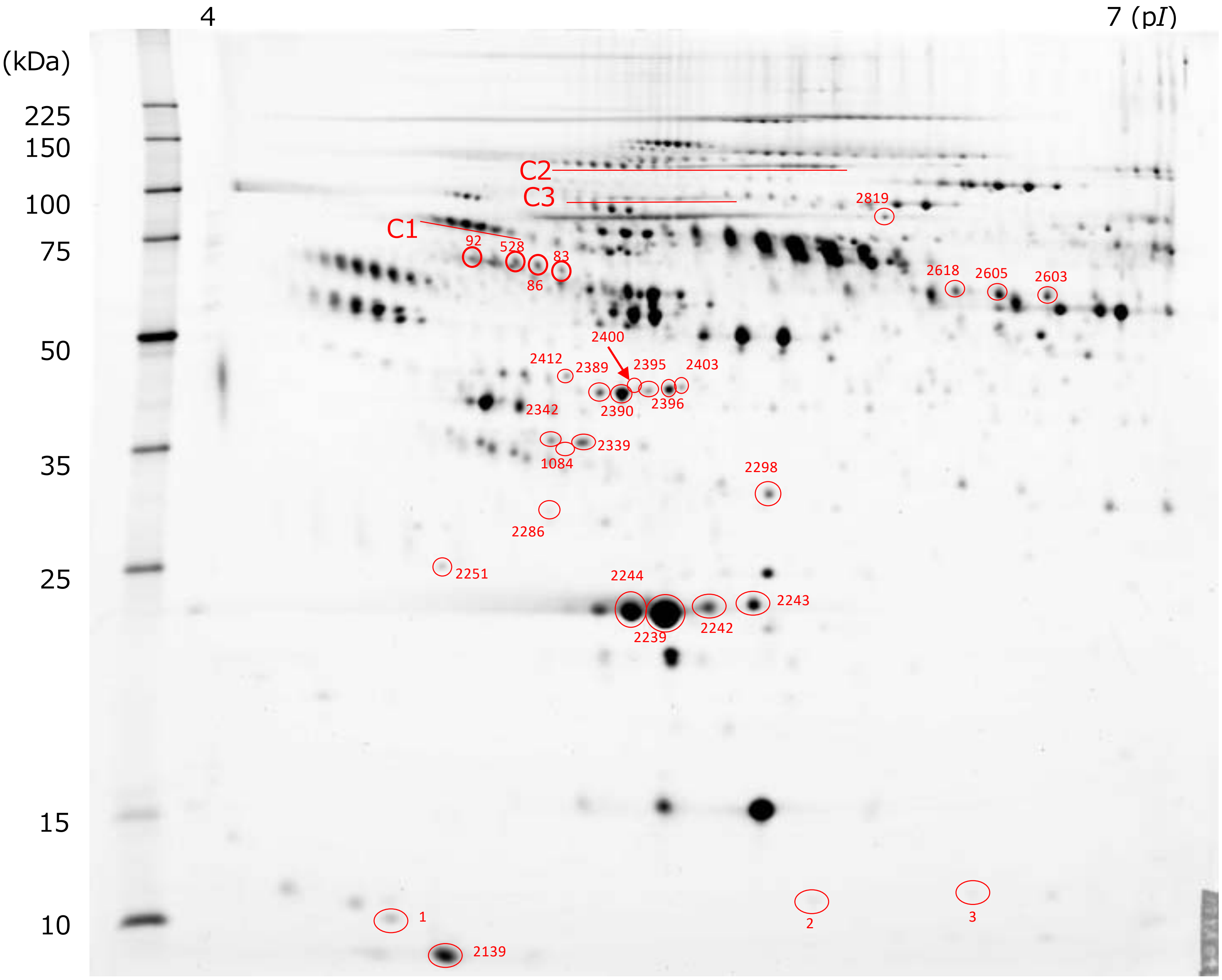

A gel image of 2D-electrophoresis stained with a SYPRO Ruby dye (TAK patient-4). Red circles indicate protein spots and spot clusters that were 1.5 times or more in the TAK patient group compared with that in the control group.

Supplementary Figure 2E

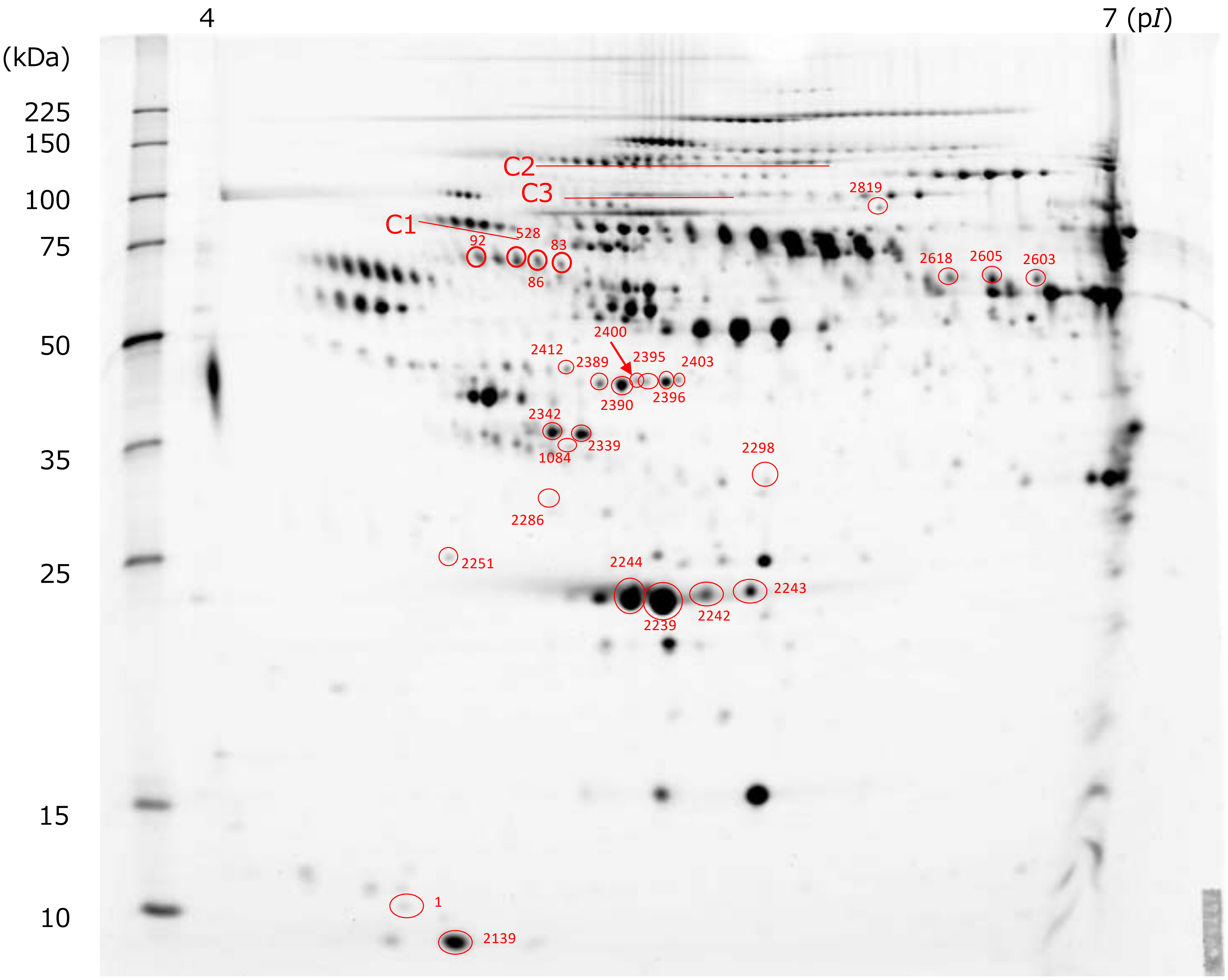

A gel image of 2D-electrophoresis stained with a SYPRO Ruby dye (TAK patient-5). Red circles indicate protein spots and spot clusters that were 1.5 times or more in the TAK patient group compared with that in the control group.

Supplementary Figure 2F

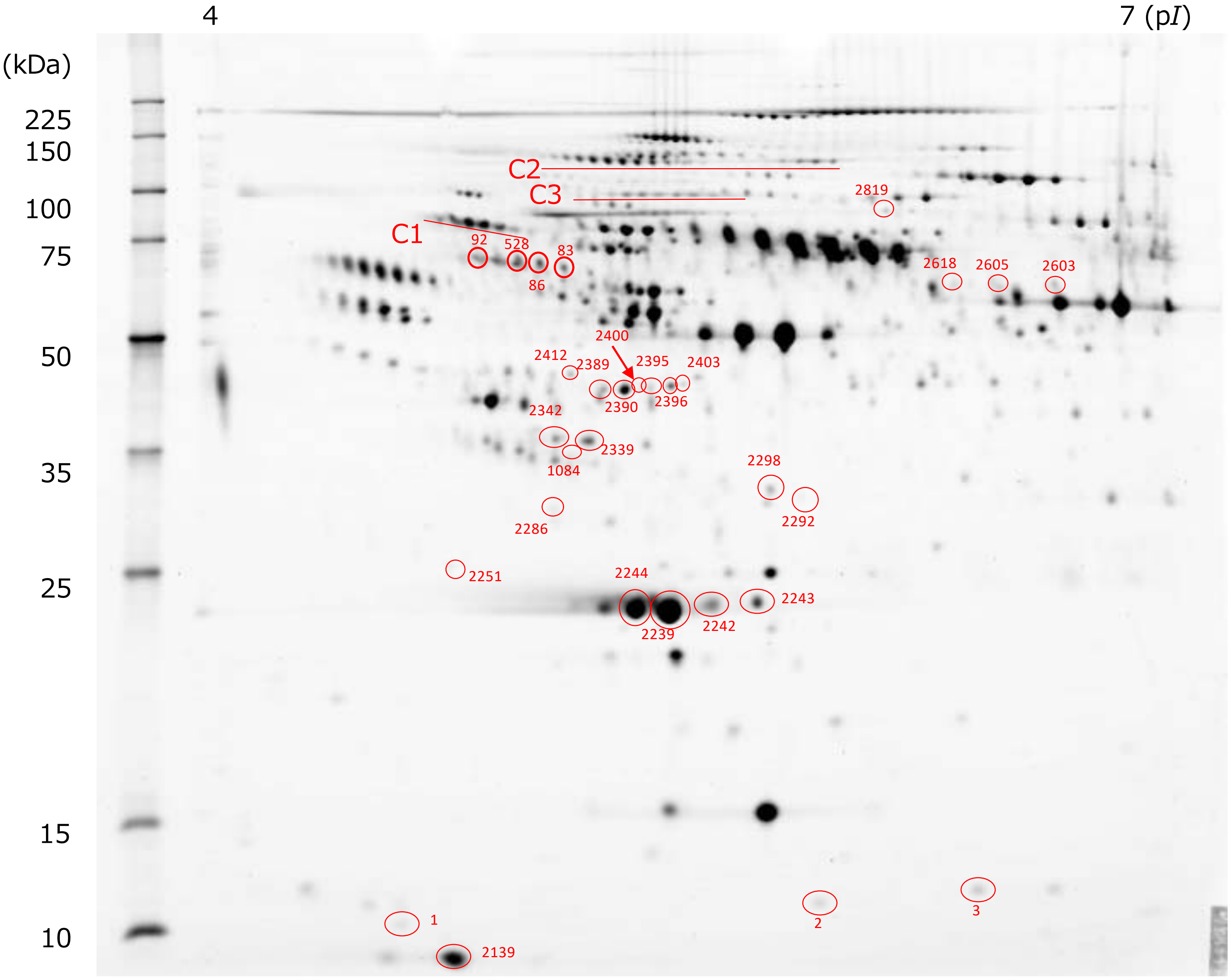

A gel image of 2D-electrophoresis stained with a SYPRO Ruby dye (TAK patient-6). Red circles indicate protein spots and spot clusters that were 1.5 times or more in the TAK patient group compared with that in the control group.

Supplementary Figure 3A

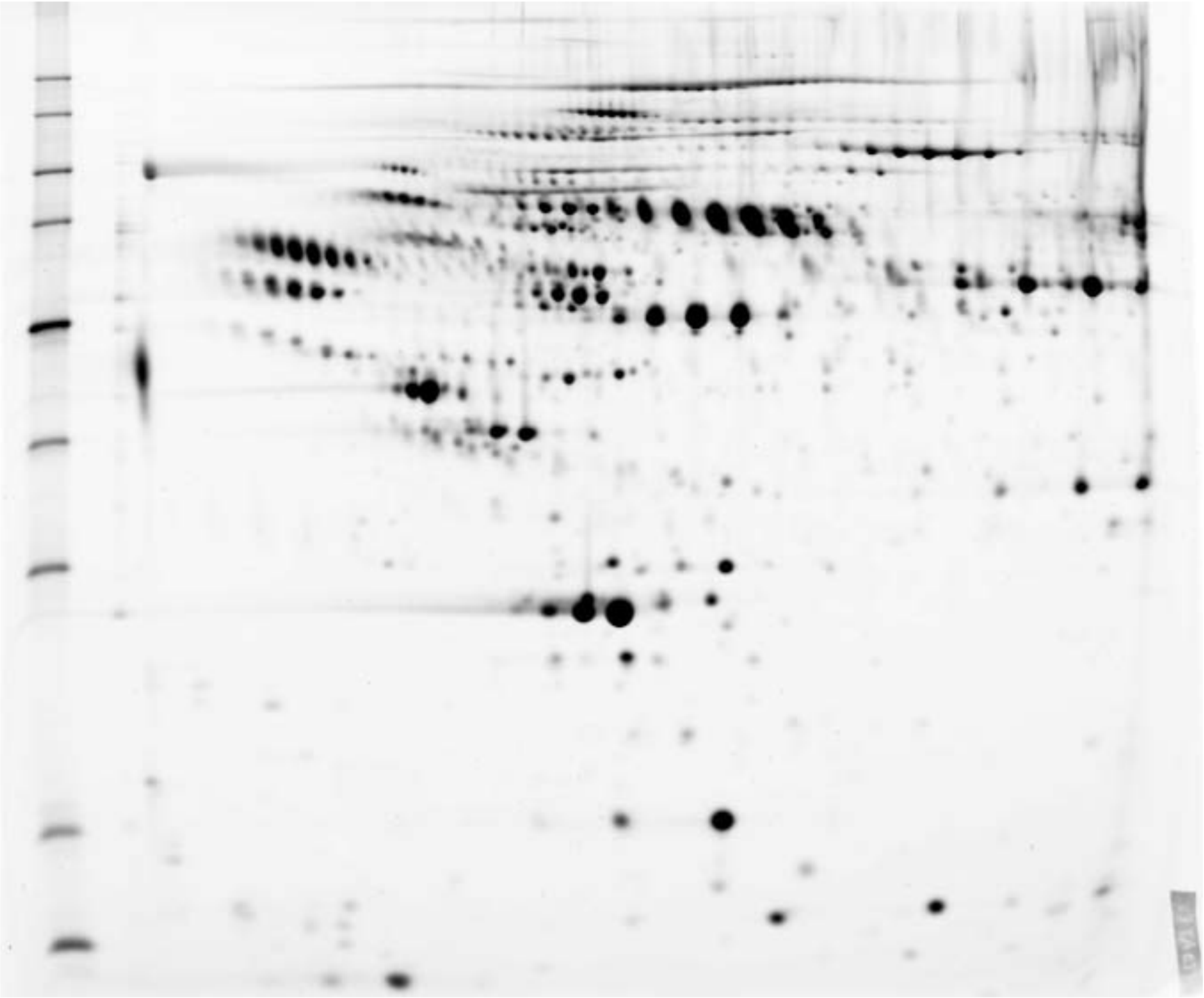

A raw gel image of 2D-electrophoresis stained with a SYPRO Ruby dye showing in Figure 1B (TAK patient-1).

Supplementary Figure 3B

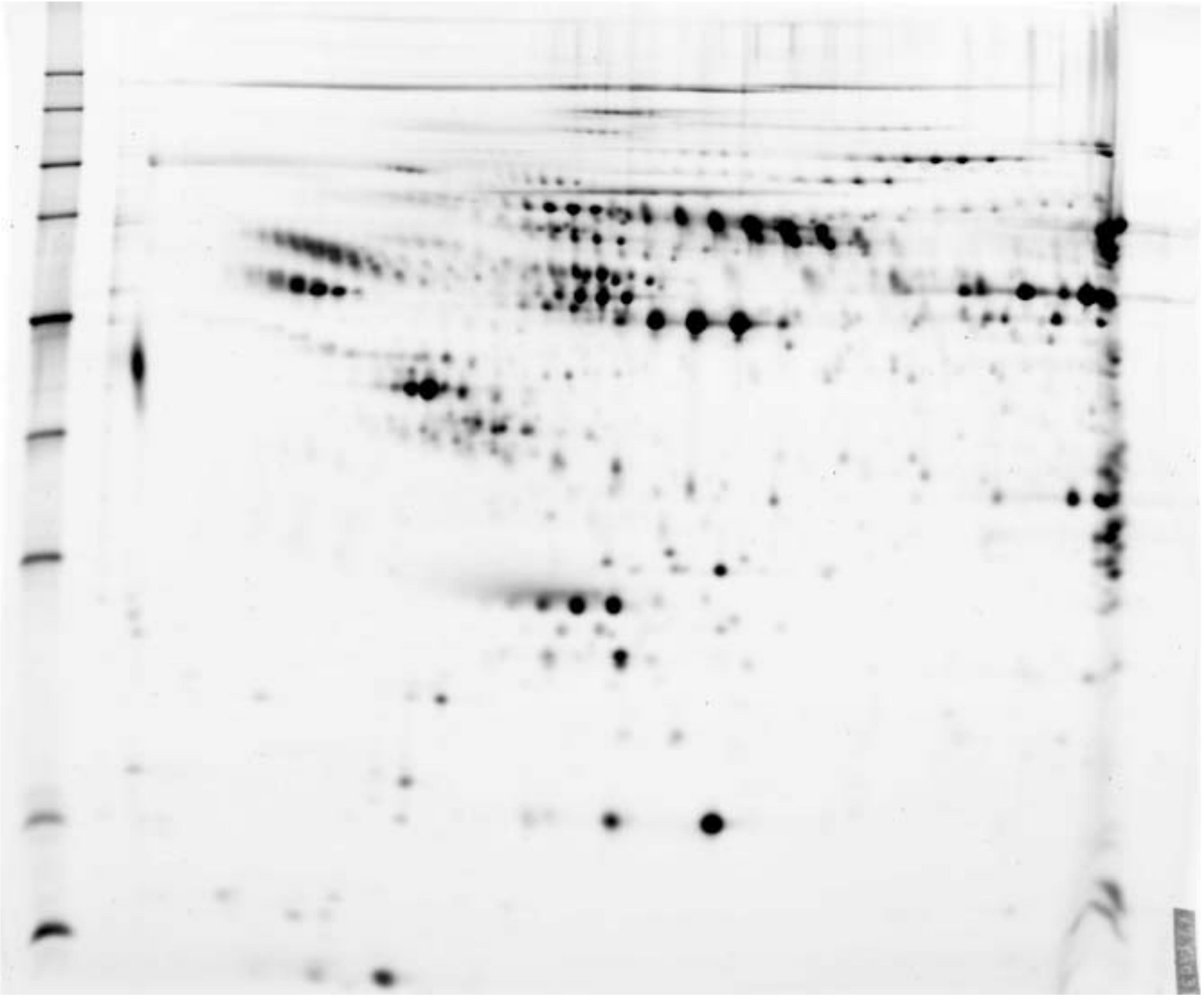

A raw gel image of 2D-electrophoresis stained with a SYPRO Ruby dye showing in Figure 2A [Healthy control] (Healthy control-6).

Supplementary Figure 3C

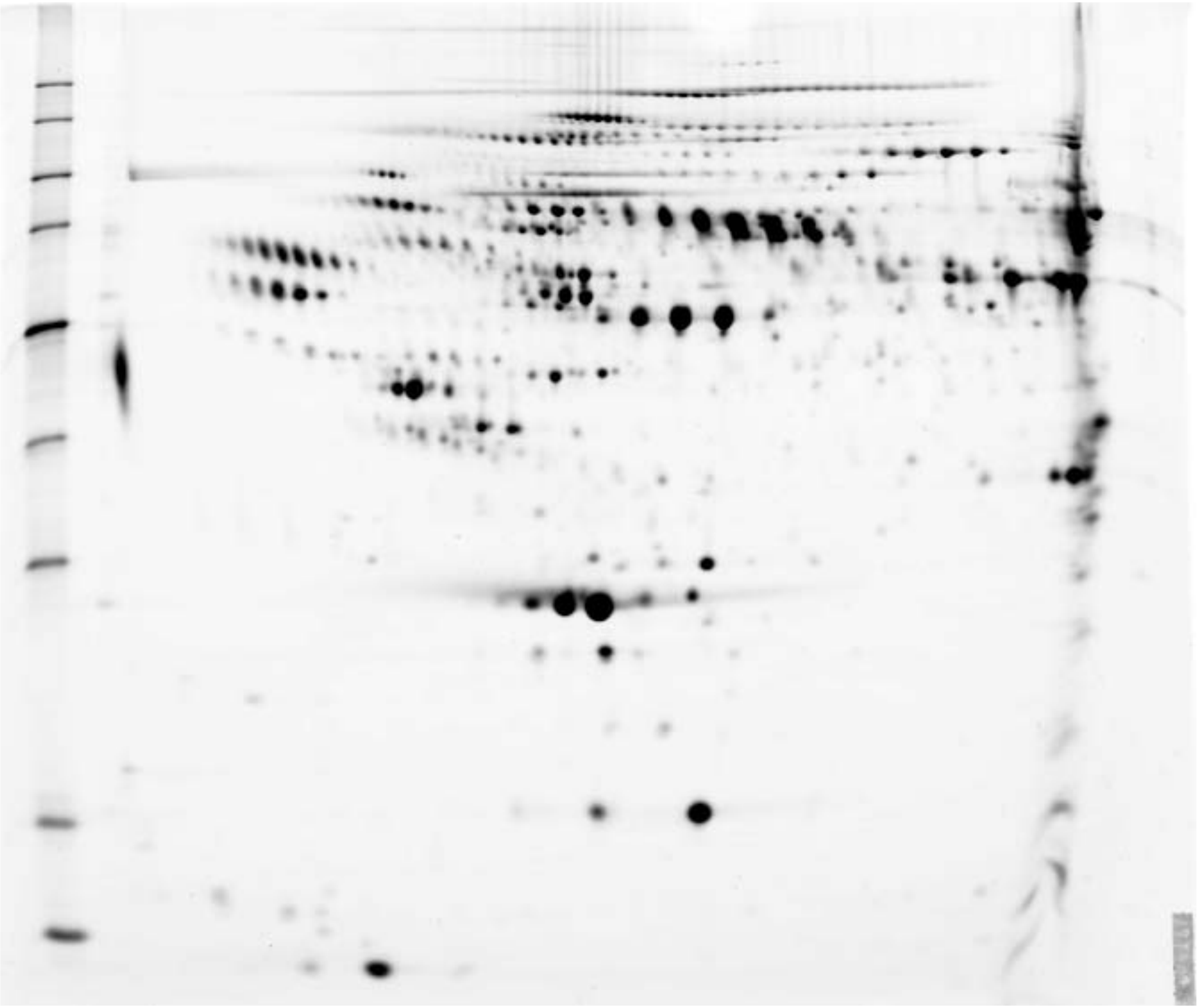

A raw gel image of 2D-electrophoresis stained with a SYPRO Ruby dye showing in both Figure 2A [TAK patients] and Figure 3B (TAK patient-5).

Supplementary Figure 4A

Match to: **gi|32130518** Score: **55**
apolipoprotein C-II precursor [Homo sapiens]
Found in search of DATA.TXT

Nominal mass (M_r): **11277**; Calculated pI value: **4.72**
NCBI BLAST search of gi|32130518 against nr
Unformatted sequence string for pasting into other applications

Taxonomy: Homo sapiens

Fixed modifications: Carbamidomethyl (C)
Variable modifications: Oxidation (M)
Cleavage by Trypsin: cuts C-term side of KR unless next residue is P
Sequence Coverage: **10%**

Matched peptides shown in **Bold Red**

1 MGTRLLPALF LVLLVLGFEV QGTQQPQQDE MPSPTFLTQV KESLSSYWES
51 AKTAAQNLYE K**TYLPAVDEK LR**DLYSKSTA AMSTYTGIFT DQVLSVLKGE
101 E

MS/MS Fragmentation of **TYLPAVDEKLR**
Found in **gi|32130518**, apolipoprotein C-II precursor [Homo sapiens]

Match to Query 3: 1303.700546 from(1304.707822,1+)
From data file DATA.TXT

Click mouse within plot area to zoom in by factor of two about that point
Or, Plot from 0 to 1200 Da

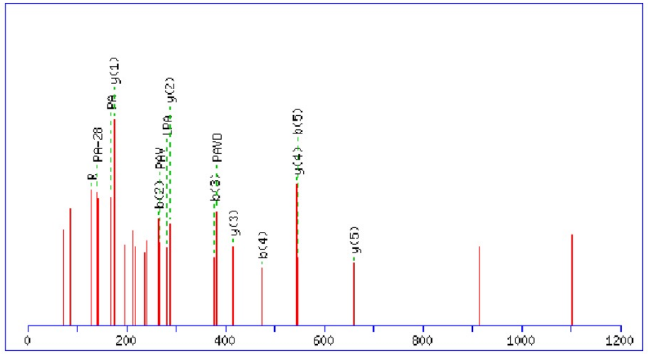

Monoisotopic mass of neutral peptide Mr(calc): 1303.71
Fixed modifications: Carbamidomethyl (C)
Ions Score: 55 **Expect:** 0.00098
Matches (Bold Red): 15/152 fragment ions using 18 most intense peaks

#	Immon.	a	a*	a^0	b	b*	b^0	Seq.	v	y	y*	y^0	#
1	74.06	74.06		56.05	102.05		84.04	T					11
2	136.08	237.12		219.11	**265.12**		247.11	Y	1095.62	1203.67	1186.65	1185.66	10
3	86.10	350.21		332.20	**378.20**		360.19	L	982.53	1040.61	1023.58	1022.60	9
4	70.07	447.26		429.25	**475.26**		457.24	P	885.48	927.53	910.50	909.52	8
5	44.05	518.30		500.29	**546.29**		528.28	A	814.44	830.47	813.45	812.46	7
6	72.08	617.37		599.36	645.36		627.35	V	715.37	759.44	742.41	741.43	6
7	88.04	732.39		714.38	760.39		742.38	D	600.35	**660.37**	643.34	642.36	5
8	102.05	861.44		843.42	889.43		871.42	E	471.30	**545.34**	528.31	527.33	4
9	101.11	989.53	972.50	971.52	1017.53	1000.50	999.51	K	343.21	**416.30**	399.27		3
10	86.10	1102.61	1085.59	1084.60	1130.61	1113.58	1112.60	L	230.12	**288.20**	271.18		2
11	**129.11**							R	74.02	**175.12**	158.09		1

Spot 1 analyzed by MS/MS ion search

Supplementary Figure 4B

Match to: **gi|4501885** Score: **546**
actin, cytoplasmic 1 [Homo sapiens]
Found in search of DATA.TXT

Nominal mass (M_r): **42052**; Calculated pI value: **5.29**
NCBI BLAST search of gi|4501885 against nr
Unformatted sequence string for pasting into other applications

Taxonomy: Homo sapiens

Fixed modifications: Carbamidomethyl (C)
Variable modifications: Oxidation (M)
Cleavage by Trypsin: cuts C-term side of KR unless next residue is P
Sequence Coverage: **32%**

Matched peptides shown in **Bold Red**

1 MDDDIAALVV DNGSGMCK**AG FAGDDAPRAV FPSIVGRPR**H QGVMVGMGQK
51 **DSYVGDEAQS KR**GILTLKYP IEHGIVTNWD DMEKIWHHTF YNELR**VAPEE**
101 **HPVLLTEAPL NPK**ANREKMT QIMFETFNTP AMYVAIQAVL SLYASGRTTG
151 IVMDSGDGVT HTVPIYEGYA LPHAILRLDL AGRDLTDYLM KILTER**GYSF**
201 **TTTAER**EIVR DIKEKLCYVA LDFEQEMATA ASSSSLEK**SY ELPDGQVITI**
251 **GNER**FRCPEA LFQPSFLGME SCGIHETTFN SIMKCDVDIR K**DLYANTVLS**
301 **GGTTMYPGIA DR**MQKEITAL APSTMK**IKII APPERK**YSVW IGGSILASLS
351 TFQQMWISK**Q EYDESGPSIV HR**KCF

MS/MS Fragmentation of **IIAPPER**
Found in **gi|4501885**, actin, cytoplasmic 1 [Homo sapiens]

Match to Query 1: 794.484516 from(795.491792,1+)
From data file DATA.TXT

Click mouse within plot area to zoom in by factor of two about that point
Or, Plot from 50 to 800 Da

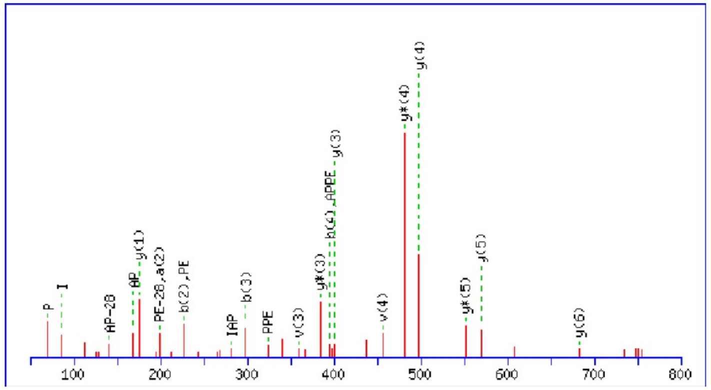

Monoisotopic mass of neutral peptide Mr(calc): 794.46
Fixed modifications: Carbamidomethyl (C)
Ions Score: 36 **Expect:** 0.049
Matches (Bold Red): 26/64 fragment ions using 30 most intense peaks

#	Immon.	a	a^0	b	b^0	Seq.	v	y	y*	y^0	#
1	**86.10**	**86.10**		114.09		I					7
2	**86.10**	**199.18**		**227.18**		I	624.31	**682.39**	665.36	664.38	6
3	44.05	270.22		**298.21**		A	553.27	**569.30**	**552.28**	551.29	5
4	**70.07**	367.27		**395.27**		P	**456.22**	**498.27**	**481.24**	480.26	4
5	**70.07**	464.32		492.32		P	**359.17**	**401.21**	**384.19**	383.20	3
6	102.05	593.37	575.36	621.36	603.35	E	230.12	304.16	287.13	286.15	2
7	129.11					R	74.02	**175.12**	158.09		1

MS/MS Fragmentation of **IIAPPERK**
Found in **gi|4501885**, actin, cytoplasmic 1 [Homo sapiens]

Match to Query 6: 922.576777 from(923.584053,1+)
From data file DATA.TXT

Click mouse within plot area to zoom in by factor of two about that point
Or, Plot from 50 to 900 Da

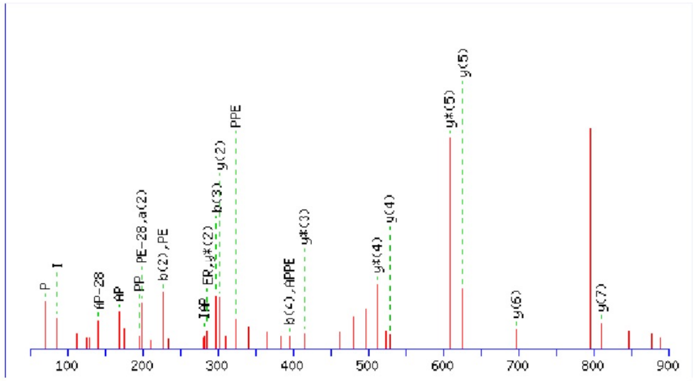

Monoisotopic mass of neutral peptide Mr(calc): 922.56
Fixed modifications: Carbamidomethyl (C)
Ions Score: 30 **Expect:** 0.15
Matches (Bold Red): 27/84 fragment ions using 35 most intense peaks

#	Immon.	a	a*	a^0	b	b*	b^0	d	Seq.	v	y	y*	y^0	#
1	**86.10**	**86.10**			114.09				I					8
2	**86.10**	**199.18**			**227.18**				I	752.40	**810.48**	793.46	792.47	7
3	44.05	270.22			**298.21**				A	681.37	**697.40**	680.37	679.39	6
4	**70.07**	367.27			**395.27**				P	584.32	**626.36**	**609.34**	608.35	5
5	**70.07**	464.32			492.32				P	487.26	**529.31**	**512.28**	511.30	4
6	102.05	593.37		575.36	621.36		603.35		E	358.22	432.26	**415.23**	414.25	3
7	129.11	749.47	732.44	731.46	777.46	760.44	759.45	664.40	R	202.12	**303.21**	**286.19**		2
8	101.11								K		147.11	130.09		1

Spot 2396 analyzed by MS/MS ion search

Supplementary Figure 4B

MS/MS Fragmentation of **AGFAGDDAPR**
Found in **gi|4501885**, actin, cytoplasmic 1 [Homo sapiens]

Match to Query 9: 975.454203 from(976.461479,1+)
From data file DATA.TXT

Click mouse within plot area to zoom in by factor of two about that point
Or, Plot from 50 to 950 Da

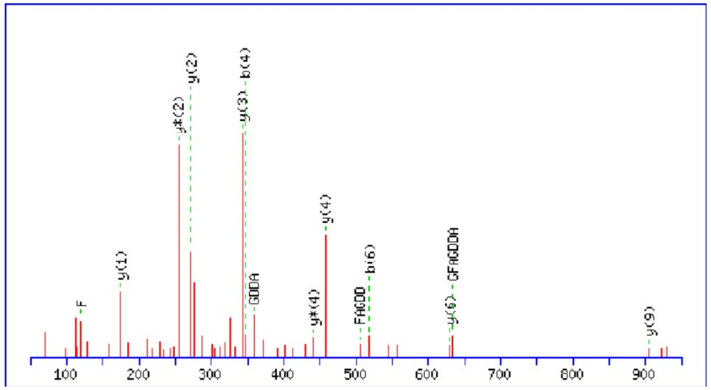

Monoisotopic mass of neutral peptide Mr(calc): 975.44
Fixed modifications: Carbamidomethyl (C)
Ions Score: 55 **Expect:** 0.0011
Matches (Bold Red): 16/121 fragment ions using 16 most intense peaks

#	Immon.	a	a^0	b	b^0	Seq.	v	y	y*	y^0	#
1	44.05	44.05		72.04		A					10
2	30.03	101.07		129.07		G		**905.41**	888.38	887.40	9
3	**120.08**	248.14		276.13		F	756.33	848.39	831.36	830.38	8
4	44.05	319.18		**347.17**		A	685.29	701.32	684.29	683.31	7
5	30.03	376.20		404.19		G		**630.28**	613.26	612.27	6
6	88.04	491.22	473.21	**519.22**	501.21	D	513.24	573.26	556.24	555.25	5
7	88.04	606.25	588.24	**634.25**	616.24	D	398.21	**458.24**	**441.21**	440.23	4
8	44.05	677.29	659.28	705.28	687.27	A	327.18	**343.21**	326.18		3
9	70.07	774.34	756.33	802.34	784.33	P	230.12	**272.17**	**255.15**		2
10	129.11					R	74.02	**175.12**	158.09		1

MS/MS Fragmentation of **IKIIAPPER**
Found in **gi|4501885**, actin, cytoplasmic 1 [Homo sapiens]

Match to Query 11: 1035.653645 from(1036.660921,1+)
From data file DATA.TXT

Click mouse within plot area to zoom in by factor of two about that point
Or, Plot from 50 to 1000 Da

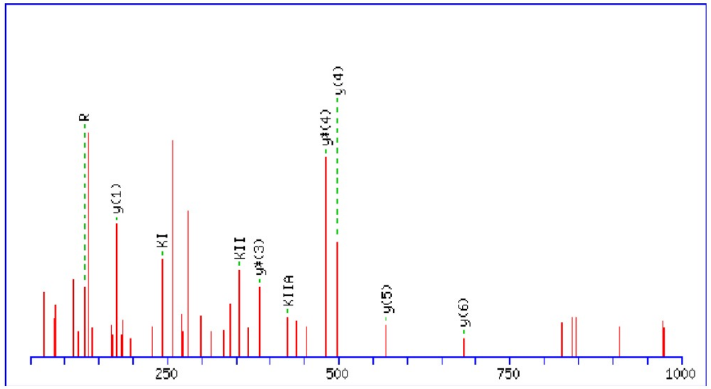

Monoisotopic mass of neutral peptide Mr(calc): 1035.64
Fixed modifications: Carbamidomethyl (C)
Ions Score: 16 **Expect:** 3
Matches (Bold Red): 12/112 fragment ions using 22 most intense peaks

#	Immon.	a	a*	a^0	b	b*	b^0	Seq.	v	y	y*	y^0	#
1	86.10	86.10			114.09			I					9
2	101.11	214.19	197.16		**242.19**	225.16		K	850.48	923.57	906.54	905.56	8
3	86.10	327.28	310.25		**355.27**	338.24		I	737.39	795.47	778.45	777.46	7
4	86.10	440.36	423.33		468.35	451.33		I	624.31	**682.39**	665.36	664.38	6
5	44.05	511.40	494.37		539.39	522.36		A	553.27	**569.30**	552.28	551.29	5
6	70.07	608.45	591.42		636.44	619.42		P	456.22	**498.27**	**481.24**	480.26	4
7	70.07	705.50	688.48		733.50	716.47		P	359.17	401.21	**384.19**	383.20	3
8	102.05	834.54	817.52	816.53	862.54	845.51	844.53	E	230.12	304.16	287.13	286.15	2
9	**129.11**							R	74.02	**175.12**	158.09		1

Spot 2396 analyzed by MS/MS ion search

Supplementary Figure 4B

MS/MS Fragmentation of **GYSFTTTAER**
Found in **gi|4501885**, actin, cytoplasmic 1 [Homo sapiens]

Match to Query 15: 1131.529396 from(1132.536672,1+)
From data file DATA.TXT

Click mouse within plot area to zoom in by factor of two about that point
Or, Plot from 0 to 1100 Da

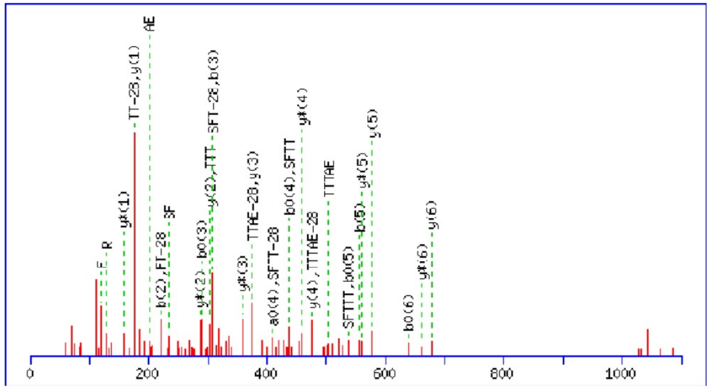

Monoisotopic mass of neutral peptide Mr(calc): 1131.52
Fixed modifications: Carbamidomethyl (C)
Ions Score: 39 **Expect:** 0.041
Matches (Bold Red): 36/126 fragment ions using 35 most intense peaks

#	Immon.	a	a^0	b	b^0	Seq.	v	y	y*	y^0	#
1	30.03	30.03		58.03		G					10
2	136.08	193.10		**221.09**		Y	967.45	1075.51	1058.48	1057.49	9
3	60.04	280.13	262.12	**308.12**	**290.11**	S	880.42	912.44	895.42	894.43	8
4	**120.08**	427.20	**409.19**	455.19	**437.18**	F	733.35	825.41	808.38	807.40	7
5	74.06	528.25	510.23	**556.24**	**538.23**	T	632.30	**678.34**	**661.32**	660.33	6
6	74.06	629.29	611.28	657.29	**639.28**	T	531.25	**577.29**	**560.27**	559.28	5
7	74.06	730.34	712.33	758.34	740.32	T	430.20	**476.25**	**459.22**	458.24	4
8	44.05	801.38	783.37	829.37	811.36	A	359.17	**375.20**	**358.17**	357.19	3
9	102.05	930.42	912.41	958.42	940.40	E	230.12	**304.16**	**287.13**	286.15	2
10	**129.11**					R	74.02	**175.12**	**158.09**		1

MS/MS Fragmentation of **AVFPSIVGRPR**
Found in **gi|4501885**, actin, cytoplasmic 1 [Homo sapiens]

Match to Query 21: 1197.709388 from(1198.716664,1+)
From data file DATA.TXT

Click mouse within plot area to zoom in by factor of two about that point
Or, Plot from 0 to 1200 Da

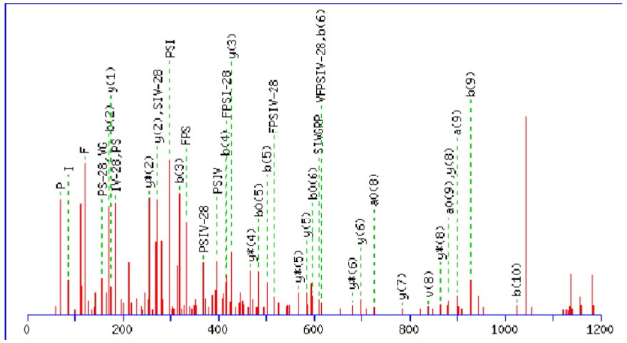

Monoisotopic mass of neutral peptide Mr(calc): 1197.70
Fixed modifications: Carbamidomethyl (C)
Ions Score: 42 **Expect:** 0.011
Matches (Bold Red): 44/144 fragment ions using 54 most intense peaks

#	Immon.	a	a*	a^0	b	b*	b^0	d	Seq.	v	y	y*	y^0	#
1	44.05	44.05			72.04				A					11
2	72.08	143.12			**171.11**				V	1083.61	1127.67	1110.64	1109.66	10
3	**120.08**	290.19			**318.18**				F	936.54	1028.60	1011.57	1010.59	9
4	**70.07**	387.24			**415.23**				P	**839.48**	**881.53**	**864.50**	863.52	8
5	60.04	474.27		456.26	**502.27**		**484.26**		S	752.45	**784.48**	767.45	766.47	7
6	**86.10**	587.36		569.34	**615.35**		**597.34**		I	639.37	**697.45**	**680.42**		6
7	72.08	686.42		668.41	714.42		696.41		V	540.30	**584.36**	**567.34**		5
8	30.03	743.45		**725.43**	771.44		753.43		G		485.29	**468.27**		4
9	129.11	**899.55**	882.52	**881.54**	**927.54**	910.51	909.53	814.48	R	327.18	**428.27**	411.25		3
10	**70.07**	996.60	979.57	978.59	**1024.59**	1007.57	1006.58	970.58	P	230.12	**272.17**	**255.15**		2
11	129.11								R	74.02	**175.12**	158.09		1

Spot 2396 analyzed by MS/MS ion search

Supplementary Figure 4B

MS/MS Fragmentation of **DSYVGDEAQSKR**
Found in **gi|4501885**, actin, cytoplasmic 1 [Homo sapiens]

Match to Query 28: 1353.635200 from(1354.642476,1+)
From data file DATA.TXT

Click mouse within plot area to zoom in by factor of two about that point
Or, Plot from 0 to 1300 Da

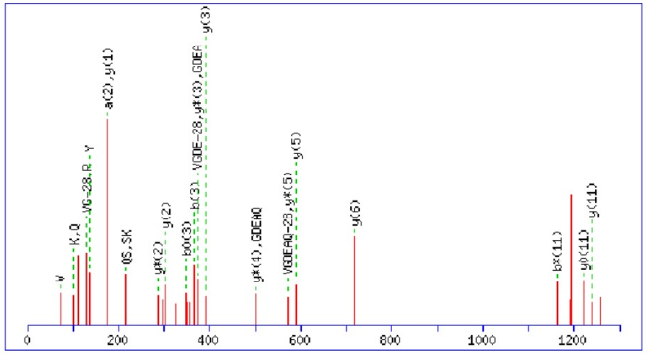

Monoisotopic mass of neutral peptide Mr(calc): 1353.62
Fixed modifications: Carbamidomethyl (C)
Ions Score: 40 **Expect:** 0.026
Matches (Bold Red): 27/177 fragment ions using 24 most intense peaks

#	Immon.	a	a*	a^0	b	b*	b^0	Seq.	v	y	y*	y^0	#
1	88.04	88.04		70.03	116.03		98.02	D					12
2	60.04	**175.07**		157.06	203.07		185.06	S	1207.57	**1239.60**	1222.57	**1221.59**	11
3	**136.08**	338.13		320.12	**366.13**		**348.12**	Y	1044.51	1152.56	1135.54	1134.55	10
4	**72.08**	437.20		419.19	465.20		447.19	V	945.44	989.50	972.47	971.49	9
5	30.03	494.22		476.21	522.22		504.21	G		890.43	873.41	872.42	8
6	88.04	609.25		591.24	637.25		619.24	D	773.39	833.41	816.38	815.40	7
7	102.05	738.29		720.28	766.29		748.28	E	644.35	**718.38**	701.36	700.37	6
8	44.05	809.33		791.32	837.33		819.32	A	573.31	**589.34**	**572.32**	571.33	5
9	**101.07**	937.39	920.36	919.38	965.38	948.36	947.37	Q	445.25	518.30	**501.28**	500.29	4
10	60.04	1024.42	1007.40	1006.41	1052.42	1035.39	1034.41	S	358.22	**390.25**	**373.22**	372.24	3
11	**101.11**	1152.52	1135.49	1134.51	1180.51	**1163.49**	1162.50	K	230.12	**303.21**	**286.19**		2
12	**129.11**							R	74.02	**175.12**	158.09		1

MS/MS Fragmentation of **QEYDESGPSIVHR**
Found in **gi|4501885**, actin, cytoplasmic 1 [Homo sapiens]

Match to Query 31: 1515.718288 from(1516.725564,1+)
From data file DATA.TXT

Click mouse within plot area to zoom in by factor of two about that point
Or, Plot from 0 to 1200 Da

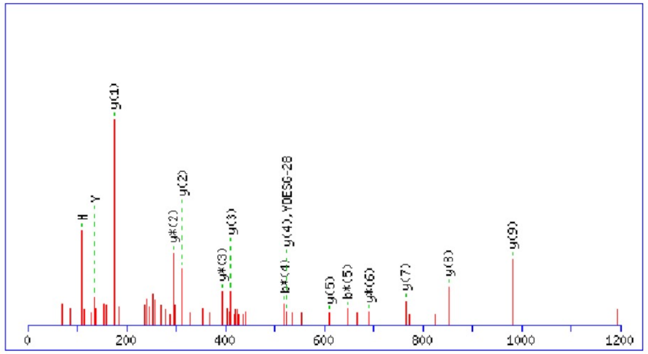

Monoisotopic mass of neutral peptide Mr(calc): 1515.70
Fixed modifications: Carbamidomethyl (C)
Ions Score: 64 **Expect:** 9.5e-005
Matches (Bold Red): 16/212 fragment ions using 19 most intense peaks

#	Immon.	a	a*	a^0	b	b*	b^0	Seq.	v	y	y*	y^0	#
1	101.07	101.07	84.04		129.07	112.04		Q					13
2	102.05	230.11	213.09	212.10	258.11	241.08	240.10	E	1314.61	1388.64	1371.62	1370.63	12
3	**136.08**	393.18	376.15	375.17	421.17	404.15	403.16	Y	1151.54	1259.60	1242.57	1241.59	11
4	88.04	508.20	491.18	490.19	536.20	**519.17**	518.19	D	1036.52	1096.54	1079.51	1078.53	10
5	102.05	637.25	620.22	619.24	665.24	**648.21**	647.23	E	907.47	**981.51**	964.48	963.50	9
6	60.04	724.28	707.25	706.27	752.27	735.25	734.26	S	820.44	**852.47**	835.44	834.46	8
7	30.03	781.30	764.27	763.29	809.29	792.27	791.28	G		**765.44**	748.41	747.43	7
8	70.07	878.35	861.33	860.34	906.35	889.32	888.34	P	666.37	708.42	**691.39**	690.40	6
9	60.04	965.38	948.36	947.37	993.38	976.35	975.37	S	579.34	**611.36**	594.34	593.35	5
10	86.10	1078.47	1061.44	1060.46	1106.46	1089.44	1088.45	I	466.25	**524.33**	507.30		4
11	72.08	1177.54	1160.51	1159.53	1205.53	1188.51	1187.52	V	367.18	**411.25**	**394.22**		3
12	**110.07**	1314.60	1297.57	1296.59	1342.59	1325.56	1324.58	H	230.12	**312.18**	**295.15**		2
13	129.11							R	74.02	**175.12**	158.09		1

Spot 2396 analyzed by MS/MS ion search

Supplementary Figure 4B

MS/MS Fragmentation of **SYELPDGQVITIGNER**
Found in **gi|4501885**, actin, cytoplasmic 1 [Homo sapiens]

Match to Query 35: 1789.896892 from(1790.904168,1+)
From data file DATA.TXT

Click mouse within plot area to zoom in by factor of two about that point
Or, Plot from 0 to 1500 Da

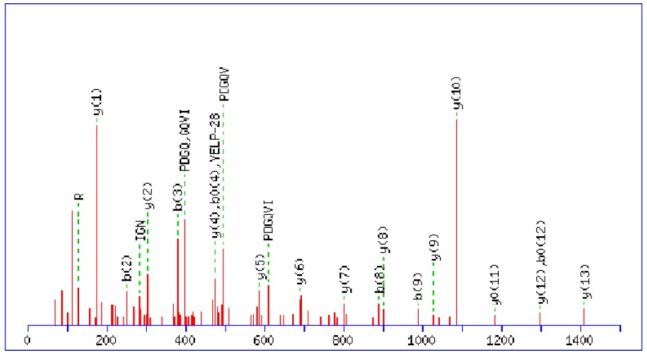

Monoisotopic mass of neutral peptide Mr(calc): 1789.88
Fixed modifications: Carbamidomethyl (C)
Ions Score: 105 **Expect:** 6.6e-009
Matches (Bold Red): 27/266 fragment ions using 25 most intense peaks

#	Immon.	a	a*	a^0	b	b*	b^0	Seq.	v	y	y*	y^0	#
1	60.04	60.04		42.03	88.04		70.03	S					16
2	136.08	223.11		205.10	**251.10**		233.09	Y	1595.80	1703.86	1686.83	1685.85	15
3	102.05	352.15		334.14	**380.15**		362.13	E	1466.76	1540.80	1523.77	1522.79	14
4	86.10	465.23		447.22	493.23		**475.22**	L	1353.68	**1411.75**	1394.73	1393.74	13
5	70.07	562.29		544.28	590.28		572.27	P	1256.62	**1298.67**	1281.64	1280.66	12
6	88.04	677.31		659.30	705.31		687.30	D	1141.60	1201.62	1184.59	**1183.61**	11
7	30.03	734.34		716.32	762.33		744.32	G		**1086.59**	1069.56	1068.58	10
8	101.07	862.39	845.37	844.38	**890.39**	873.36	872.38	Q	956.52	**1029.57**	1012.54	1011.56	9
9	72.08	961.46	944.44	943.45	**989.46**	972.43	971.45	V	857.45	**901.51**	884.48	883.50	8
10	86.10	1074.55	1057.52	1056.54	1102.54	1085.51	1084.53	I	744.36	**802.44**	785.42	784.43	7
11	74.06	1175.59	1158.57	1157.58	1203.59	1186.56	1185.58	T	643.32	**689.36**	672.33	671.35	6
12	86.10	1288.68	1271.65	1270.67	1316.67	1299.65	**1298.66**	I	530.23	**588.31**	571.28	570.30	5
13	30.03	1345.70	1328.67	1327.69	1373.69	1356.67	1355.68	G		**475.23**	458.20	457.22	4
14	87.06	1459.74	1442.72	1441.73	1487.74	1470.71	1469.73	N	359.17	418.20	401.18	400.19	3
15	102.05	1588.79	1571.76	1570.77	1616.78	1599.75	1598.77	E	230.12	**304.16**	287.13	286.15	2
16	**129.11**							R	74.02	**175.12**	158.09		1

MS/MS Fragmentation of **VAPEEHPVLLTEAPLNPK**
Found in **gi|4501885**, actin, cytoplasmic 1 [Homo sapiens]

Match to Query 37: 1953.065572 from(1954.072848,1+)
From data file DATA.TXT

Click mouse within plot area to zoom in by factor of two about that point
Or, Plot from 0 to 1900 Da

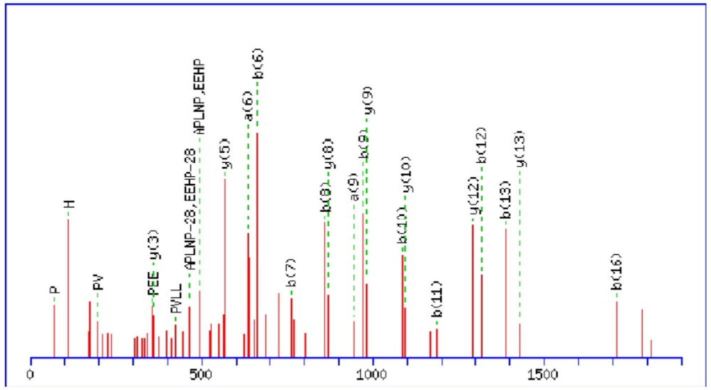

Monoisotopic mass of neutral peptide Mr(calc): 1953.06
Fixed modifications: Carbamidomethyl (C)
Ions Score: 81 **Expect:** 8.6e-007
Matches (Bold Red): 32/261 fragment ions using 29 most intense peaks

#	Immon.	a	a*	a^0	b	b*	b^0	Seq.	y	y*	y^0	#
1	72.08	72.08			100.08			V				18
2	44.05	143.12			171.11			A	1855.00	1837.97	1836.99	17
3	**70.07**	240.17			268.17			P	1783.96	1766.93	1765.95	16
4	102.05	369.21		351.20	397.21		379.20	E	1686.91	1669.88	1668.90	15
5	102.05	498.26		480.25	526.25		508.24	E	1557.86	1540.84	1539.85	14
6	**110.07**	**635.31**		617.30	**663.31**		645.30	H	**1428.82**	1411.79	1410.81	13
7	**70.07**	732.37		714.36	**760.36**		742.35	P	**1291.76**	1274.74	1273.75	12
8	72.08	831.44		813.43	**859.43**		841.42	V	1194.71	1177.68	1176.70	11
9	86.10	**944.52**		926.51	**972.51**		954.50	L	**1095.64**	1078.61	1077.63	10
10	86.10	1057.60		1039.59	**1085.60**		1067.59	L	**982.56**	965.53	964.55	9
11	74.06	1158.65		1140.64	**1186.65**		1168.64	T	**869.47**	852.45	851.46	8
12	102.05	1287.69		1269.68	**1315.69**		1297.68	E	768.43	751.40	750.41	7
13	44.05	1358.73		1340.72	**1386.73**		1368.72	A	639.38	622.36		6
14	**70.07**	1455.78		1437.77	1483.78		1465.77	P	**568.35**	551.32		5
15	86.10	1568.87		1550.86	1596.86		1578.85	L	471.29	454.27		4
16	87.06	1682.91	1665.88	1664.90	**1710.91**	1693.88	1692.90	N	**358.21**	341.18		3
17	**70.07**	1779.96	1762.94	1761.95	1807.96	1790.93	1789.95	P	244.17	227.14		2
18	101.11							K	147.11	130.09		1

Spot 2396 analyzed by MS/MS ion search

Supplementary Figure 4B

MS/MS Fragmentation of **DLYANTVLSGGTTMYPGIADR**
Found in **gi|4501885**, actin, cytoplasmic 1 [Homo sapiens]

Match to Query 39: 2230.056790 from(2231.064066,1+)
From data file DATA.TXT

Click mouse within plot area to zoom in by factor of two about that point
Or, Plot from 0 to 2200 Da

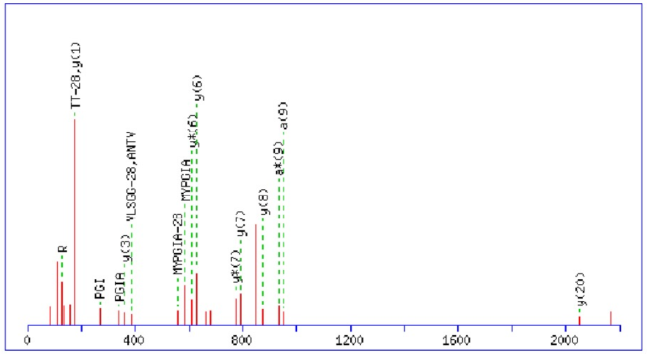

Monoisotopic mass of neutral peptide Mr(calc): 2230.06
Fixed modifications: Carbamidomethyl (C)
Variable modifications:
M14 : Oxidation (M)
Ions Score: 38 **Expect:** 0.025
Matches (Bold Red): 18/394 fragment ions using 20 most intense peaks

#	Immon.	a	a*	a^0	b	b*	b^0	Seq.	v	y	y*	y^0	#
1	88.04	88.04		70.03	116.03		98.02	D					21
2	86.10	201.12		183.11	229.12		211.11	L	1993.96	**2052.04**	2035.01	2034.03	20
3	136.08	364.19		346.18	392.18		374.17	Y	1830.90	1938.96	1921.93	1920.94	19
4	44.05	435.22		417.21	463.22		445.21	A	1759.86	1775.89	1758.87	1757.88	18
5	87.06	549.27	532.24	531.26	577.26	560.24	559.25	N	1645.82	1704.86	1687.83	1686.84	17
6	74.06	650.31	633.29	632.30	678.31	661.28	660.30	T	1544.77	1590.81	1573.79	1572.80	16
7	72.08	749.38	732.36	731.37	777.38	760.35	759.37	V	1445.70	1489.76	1472.74	1471.75	15
8	86.10	862.47	845.44	844.46	890.46	873.44	872.45	L	1332.62	1390.70	1373.67	1372.69	14
9	60.04	**949.50**	**932.47**	931.49	977.49	960.47	959.48	S	1245.59	1277.61	1260.59	1259.60	13
10	30.03	1006.52	989.49	988.51	1034.52	1017.49	1016.50	G		1190.58	1173.55	1172.57	12
11	30.03	1063.54	1046.52	1045.53	1091.54	1074.51	1073.53	G		1133.56	1116.53	1115.55	11
12	74.06	1164.59	1147.56	1146.58	1192.58	1175.56	1174.57	T	1030.50	1076.54	1059.51	1058.53	10
13	74.06	1265.64	1248.61	1247.63	1293.63	1276.61	1275.62	T	929.45	975.49	958.46	957.48	9
14	56.05	1348.67	1331.65	1330.66	1376.67	1359.64	1358.66	M	846.41	**874.44**	857.42	856.43	8
15	136.08	1511.74	1494.71	1493.73	1539.73	1522.71	1521.72	Y	683.35	**791.40**	**774.38**	773.39	7
16	70.07	1608.79	1591.76	1590.78	1636.79	1619.76	1618.77	P	586.29	**628.34**	**611.31**	610.33	6
17	30.03	1665.81	1648.79	1647.80	1693.81	1676.78	1675.80	G		531.29	514.26	513.28	5
18	86.10	1778.90	1761.87	1760.89	1806.89	1789.86	1788.88	I	416.19	474.27	457.24	456.26	4
19	44.05	1849.93	1832.91	1831.92	1877.93	1860.90	1859.92	A	345.15	**361.18**	344.16	343.17	3
20	88.04	1964.96	1947.93	1946.95	1992.95	1975.93	1974.94	D	230.12	290.15	273.12	272.14	2
21	**129.11**							R	74.02	**175.12**	158.09		1

Spot 2396 analyzed by MS/MS ion search

Supplementary Figure 4C

Match to: **gi|4557321** Score: **396**
apolipoprotein A-I isoform 1 preproprotein [Homo sapiens]
Found in search of DATA.TXT

Nominal mass (M_r): **30759**; Calculated pI value: **5.56**
NCBI BLAST search of gi|4557321 against nr
Unformatted sequence string for pasting into other applications

Taxonomy: Homo sapiens

Fixed modifications: Carbamidomethyl (C)
Variable modifications: Oxidation (M)
Cleavage by Trypsin: cuts C-term side of KR unless next residue is P
Sequence Coverage: **37%**

Matched peptides shown in **Bold Red**

```
  1 MKAAVLTLAV LFLTGSQARH FWQQDEPPQS PWDRVKDLAT VYVDVLKDSG
 51 RDYVSQFEGS ALGKQLNLKL LDNWDSVTST FSKLREQLGP VTQEFWDNLE
101 KETEGLRQEM SKDLEEVKAK VQPYLDDFQK KWQEEMELYR QKVEPLRAEL
151 QEGARQKLHE LQEKLSPLGE EMRDRARAHV DALRTHLAPY SDELRQRLAA
201 RLEALKENGG ARLAEYHAKA TEHLSTLSEK AKPALEDLRQ GLLPVLESFK
251 VSFLSALEEY TKKLNTQ
```

MS/MS Fragmentation of **AHVDALR**
Found in **gi|4557321**, apolipoprotein A-I isoform 1 preproprotein [Homo sapiens]

Match to Query 1: 780.444614 from(781.451890,1+)
From data file DATA.TXT

Click mouse within plot area to zoom in by factor of two about that point
Or, [Plot from] [50] to [750] Da

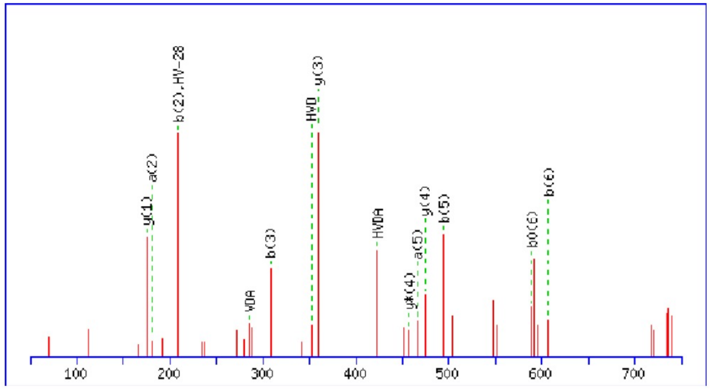

Monoisotopic mass of neutral peptide Mr(calc): 780.42
Fixed modifications: Carbamidomethyl (C)
Ions Score: 31 **Expect:** 0.25
Matches (Bold Red): 16/66 fragment ions using 27 most intense peaks

#	Immon.	a	a^0	b	b^0	Seq.	v	y	y*	y^0	#
1	44.05	44.05		72.04		A					7
2	110.07	**181.11**		**209.10**		H	628.34	710.39	693.37	692.38	6
3	72.08	280.18		**308.17**		V	529.27	573.34	556.31	555.32	5
4	88.04	395.20	377.19	**423.20**	405.19	D	414.25	**474.27**	**457.24**	456.26	4
5	44.05	**466.24**	448.23	**494.24**	476.23	A	343.21	**359.24**	342.21		3
6	86.10	579.32	561.31	**607.32**	**589.31**	L	230.12	288.20	271.18		2
7	129.11					R	74.02	**175.12**	158.09		1

Spot 2243 analyzed by MS/MS ion search

MS/MS Fragmentation of **LAEYHAK**
Found in **gi|4557321**, apolipoprotein A-I isoform 1 preproprotein [Homo sapiens]

Match to Query 2: 830.436220 from(831.443496,1+)
From data file DATA.TXT

Click mouse within plot area to zoom in by factor of two about that point
Or, [Plot from] [50] to [800] Da

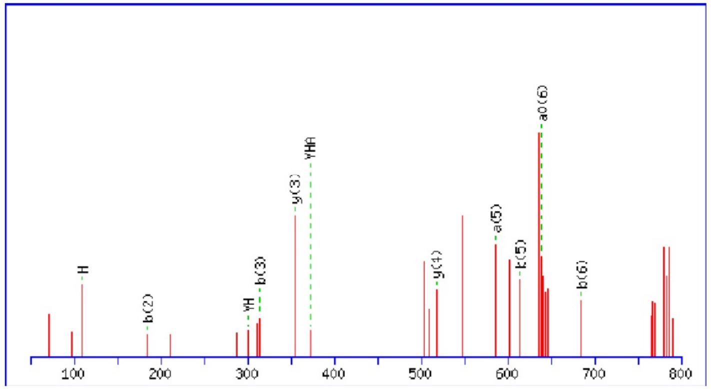

Monoisotopic mass of neutral peptide Mr(calc): 830.43
Fixed modifications: Carbamidomethyl (C)
Ions Score: 13 **Expect:** 24
Matches (Bold Red): 11/61 fragment ions using 31 most intense peaks

#	Immon.	a	a^0	b	b^0	Seq.	y	y*	y^0	#
1	86.10	86.10		114.09		L				7
2	44.05	157.13		**185.13**		A	718.35	701.33	700.34	6
3	102.05	286.18	268.17	**314.17**	296.16	E	647.31	630.29	629.30	5
4	136.08	449.24	431.23	477.23	459.22	Y	**518.27**	501.25		4
5	**110.07**	**586.30**	568.29	**614.29**	596.28	H	**355.21**	338.18		3
6	44.05	657.34	**639.32**	**685.33**	667.32	A	218.15	201.12		2
7	101.11					K	147.11	130.09		1

Supplementary Figure 4C

MS/MS Fragmentation of **QKVEPLR**
Found in **gi|4557321**, apolipoprotein A-I isoform 1 preproprotein [Homo sapiens]

Match to Query 6: 868.518712 from(869.525988,1+)
From data file DATA.TXT

Click mouse within plot area to zoom in by factor of two about that point
Or, Plot from 50 to 900 Da

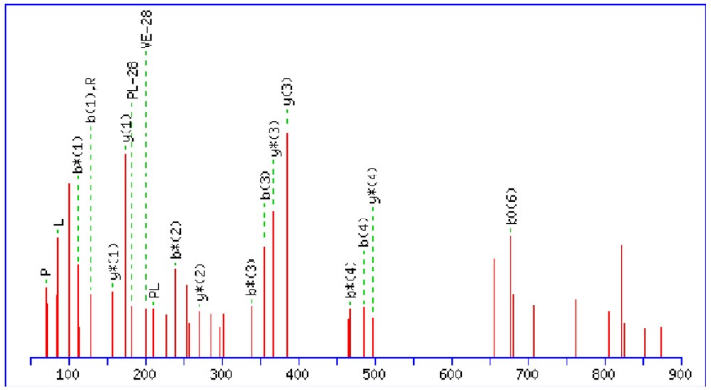

Monoisotopic mass of neutral peptide Mr(calc): 868.51
Fixed modifications: Carbamidomethyl (C)
Ions Score: 16 **Expect:** 5
Matches (Bold Red): 20/78 fragment ions using 33 most intense peaks

#	Immon.	a	a*	a^0	b	b*	b^0	Seq.	v	y	y*	y^0	#
1	101.07	101.07	84.04		**129.07**	**112.04**		Q					7
2	101.11	229.17	212.14		257.16	**240.13**		K	668.37	741.46	724.44	723.45	6
3	72.08	328.23	311.21		**356.23**	**339.20**		V	569.30	613.37	596.34	595.36	5
4	102.05	457.28	440.25	439.27	**485.27**	**468.25**	467.26	E	440.26	514.30	**497.27**	496.29	4
5	**70.07**	554.33	537.30	536.32	582.32	565.30	564.31	P	343.21	**385.26**	**368.23**		3
6	**86.10**	667.41	650.39	649.40	695.41	678.38	**677.40**	L	230.12	288.20	**271.18**		2
7	**129.11**							R	74.02	**175.12**	**158.09**		1

MS/MS Fragmentation of **AELQEGAR**
Found in **gi|4557321**, apolipoprotein A-I isoform 1 preproprotein [Homo sapiens]

Match to Query 7: 872.437001 from(873.444277,1+)
From data file DATA.TXT

Click mouse within plot area to zoom in by factor of two about that point
Or, Plot from 50 to 850 Da

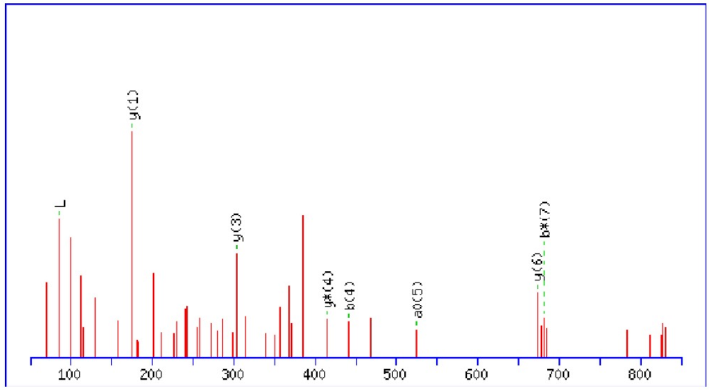

Monoisotopic mass of neutral peptide Mr(calc): 872.44
Fixed modifications: Carbamidomethyl (C)
Ions Score: 15 **Expect:** 14
Matches (Bold Red): 8/96 fragment ions using 11 most intense peaks

#	Immon.	a	a*	a^0	b	b*	b^0	Seq.	v	y	y*	y^0	#
1	44.05	44.05			72.04			A					8
2	102.05	173.09		155.08	201.09		183.08	E	728.37	802.41	785.38	784.39	7
3	**86.10**	286.18		268.17	314.17		296.16	L	615.28	**673.36**	656.34	655.35	6
4	101.07	414.23	397.21	396.22	**442.23**	425.20	424.22	Q	487.23	560.28	543.25	542.27	5
5	102.05	543.28	526.25	**525.27**	571.27	554.25	553.26	E	358.18	432.22	**415.19**	414.21	4
6	30.03	600.30	583.27	582.29	628.29	611.27	610.28	G		**303.18**	286.15		3
7	44.05	671.34	654.31	653.33	699.33	**682.30**	681.32	A	230.12	246.16	229.13		2
8	129.11							R	74.02	**175.12**	158.09		1

Spot 2243 analyzed by MS/MS ion search

Supplementary Figure 4C

MS/MS Fragmentation of **AKPALEDLR**
Found in **gi|4557321**, apolipoprotein A-I isoform 1 preproprotein [Homo sapiens]

Match to Query 9: 1011.558825 from(1012.566101,1+)
From data file DATA.TXT

Click mouse within plot area to zoom in by factor of two about that point
Or, Plot from 50 to 1000 Da

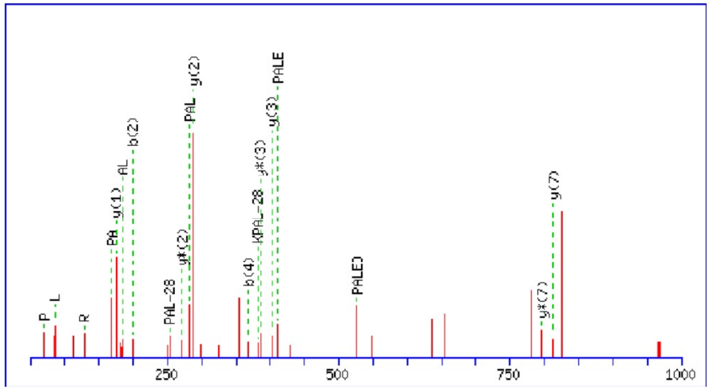

Monoisotopic mass of neutral peptide Mr(calc): 1011.57
Fixed modifications: Carbamidomethyl (C)
Ions Score: 21 **Expect:** 2.2
Matches (**Bold Red**): 20/115 fragment ions using 28 most intense peaks

#	Immon.	a	a*	a^0	b	b*	b^0	Seq.	v	y	y*	y^0	#
1	44.05	44.05			72.04			A					9
2	101.11	172.14	155.12		**200.14**	183.11		K	868.45	941.54	924.51	923.53	8
3	**70.07**	269.20	252.17		297.19	280.17		P	771.40	**813.45**	**796.42**	795.44	7
4	44.05	340.23	323.21		**368.23**	351.20		A	700.36	716.39	699.37	698.38	6
5	**86.10**	453.32	436.29		481.31	464.29		L	587.28	645.36	628.33	627.35	5
6	102.05	582.36	565.33	564.35	610.36	593.33	592.35	E	458.24	532.27	515.25	514.26	4
7	88.04	697.39	680.36	679.38	725.38	708.36	707.37	D	343.21	**403.23**	**386.20**	385.22	3
8	**86.10**	810.47	793.45	792.46	838.47	821.44	820.46	L	230.12	**288.20**	**271.18**		2
9	**129.11**							R	74.02	**175.12**	158.09		1

MS/MS Fragmentation of **QKLHELQEK**
Found in **gi|4557321**, apolipoprotein A-I isoform 1 preproprotein [Homo sapiens]

Match to Query 14: 1151.597464 from(1152.604740,1+)
From data file DATA.TXT

Click mouse within plot area to zoom in by factor of two about that point
Or, Plot from 0 to 1200 Da

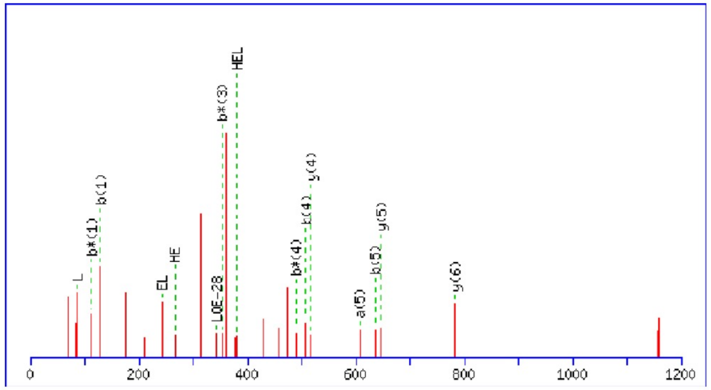

Monoisotopic mass of neutral peptide Mr(calc): 1151.63
Fixed modifications: Carbamidomethyl (C)
Ions Score: 14 **Expect:** 11
Matches (**Bold Red**): 18/108 fragment ions using 24 most intense peaks

#	Immon.	a	a*	a^0	b	b*	b^0	Seq.	y	y*	y^0	#
1	101.07	101.07	84.04		**129.07**	**112.04**		Q				9
2	101.11	229.17	212.14		257.16	240.13		K	1024.58	1007.55	1006.57	8
3	**86.10**	342.25	325.22		370.24	**353.22**		L	896.48	879.46	878.47	7
4	110.07	479.31	462.28		**507.30**	**490.28**		H	**783.40**	766.37	765.39	6
5	102.05	**608.35**	591.32	590.34	**636.35**	619.32	618.34	E	**646.34**	629.31	628.33	5
6	**86.10**	721.44	704.41	703.42	749.43	732.40	731.42	L	**517.30**	500.27	499.29	4
7	101.07	849.49	832.47	831.48	877.49	860.46	859.48	Q	404.21	387.19	386.20	3
8	102.05	978.54	961.51	960.53	1006.53	989.51	988.52	E	276.16	259.13	258.14	2
9	101.11							K	147.11	130.09		1

Spot 2243 analyzed by MS/MS ion search

Supplementary Figure 4C

MS/MS Fragmentation of **VQPYLDDFQK**
Found in **gi|4557321**, apolipoprotein A-I isoform 1 preproprotein [Homo sapiens]

Match to Query 16: 1251.590959 from(1252.598235,1+)
From data file DATA.TXT

Click mouse within plot area to zoom in by factor of two about that point
Or, Plot from 0 to 1200 Da

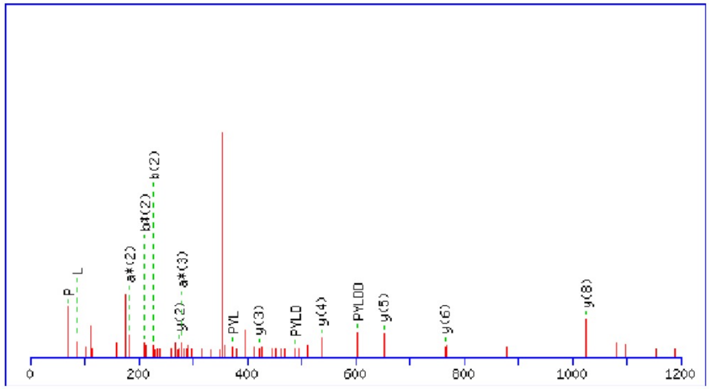

Monoisotopic mass of neutral peptide Mr(calc): 1251.61
Fixed modifications: Carbamidomethyl (C)
Ions Score: 19 **Expect:** 2.8
Matches (Bold Red): 15/120 fragment ions using 34 most intense peaks

#	Immon.	a	a*	a^0	b	b*	b^0	Seq.	y	y*	y^0	#
1	72.08	72.08			100.08			V				10
2	101.07	200.14	**183.11**		**228.13**	**211.11**		Q	1153.55	1136.53	1135.54	9
3	**70.07**	297.19	**280.17**		325.19	308.16		P	**1025.49**	1008.47	1007.48	8
4	136.08	460.26	443.23		488.25	471.22		Y	928.44	911.41	910.43	7
5	**86.10**	573.34	556.31		601.33	584.31		L	**765.38**	748.35	747.37	6
6	88.04	688.37	671.34	670.36	716.36	699.33	698.35	D	**652.29**	635.27	634.28	5
7	88.04	803.39	786.37	785.38	831.39	814.36	813.38	D	**537.27**	520.24	519.26	4
8	120.08	950.46	933.44	932.45	978.46	961.43	960.45	F	**422.24**	405.21		3
9	101.07	1078.52	1061.49	1060.51	1106.52	1089.49	1088.50	Q	**275.17**	258.14		2
10	101.11							K	147.11	130.09		1

MS/MS Fragmentation of **THLAPYSDELR**
Found in **gi|4557321**, apolipoprotein A-I isoform 1 preproprotein [Homo sapiens]

Match to Query 19: 1300.623319 from(1301.630595,1+)
From data file DATA.TXT

Click mouse within plot area to zoom in by factor of two about that point
Or, Plot from 0 to 1300 Da

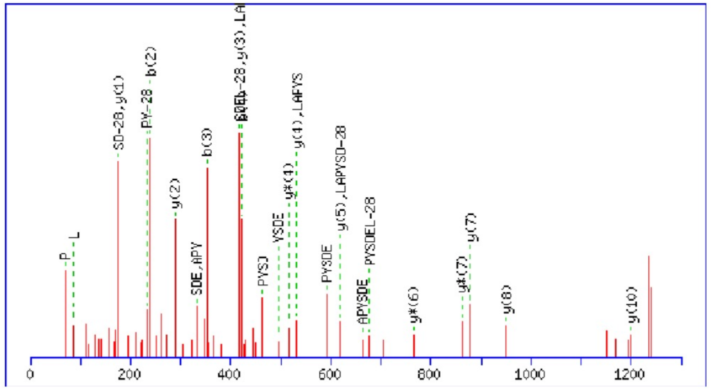

Monoisotopic mass of neutral peptide Mr(calc): 1300.64
Fixed modifications: Carbamidomethyl (C)
Ions Score: 68 **Expect:** 3.9e-005
Matches (Bold Red): 30/148 fragment ions using 29 most intense peaks

#	Immon.	a	a^0	b	b^0	Seq.	v	y	y*	y^0	#
1	74.06	74.06	56.05	102.05	84.04	T					11
2	110.07	211.12	193.11	**239.11**	221.10	H	1118.55	**1200.60**	1183.57	1182.59	10
3	**86.10**	324.20	306.19	**352.20**	334.19	L	1005.46	1063.54	1046.52	1045.53	9
4	44.05	395.24	377.23	**423.24**	405.22	A	934.43	**950.46**	933.43	932.45	8
5	**70.07**	492.29	474.28	520.29	502.28	P	837.37	**879.42**	**862.39**	861.41	7
6	136.08	655.36	637.35	683.35	665.34	Y	674.31	782.37	**765.34**	764.36	6
7	60.04	742.39	724.38	770.38	752.37	S	587.28	**619.30**	602.28	601.29	5
8	88.04	857.42	839.40	885.41	867.40	D	472.25	**532.27**	**515.25**	514.26	4
9	102.05	986.46	968.45	1014.45	996.44	E	343.21	**417.25**	400.22	399.24	3
10	**86.10**	1099.54	1081.53	1127.54	1109.53	L	230.12	**288.20**	271.18		2
11	129.11					R	74.02	**175.12**	158.09		1

Spot 2243 analyzed by MS/MS ion search

Supplementary Figure 4C

MS/MS Fragmentation of **LSPLGEEMRDR**
Found in **gi|4557321**, apolipoprotein A-I isoform 1 preproprotein [Homo sapiens]

Match to Query 20: 1317.609182 from(1318.616458,1+)
From data file DATA.TXT

Click mouse within plot area to zoom in by factor of two about that point
Or, Plot from 0 to 1400 Da

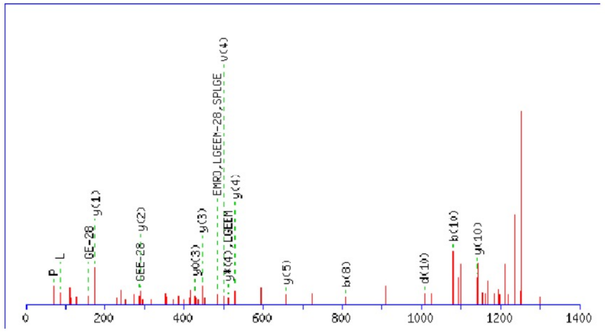

Monoisotopic mass of neutral peptide Mr(calc): 1317.63
Fixed modifications: Carbamidomethyl (C)
Variable modifications:
M8 : Oxidation (M)
Ions Score: 31 **Expect:** 0.19
Matches (Bold Red): 22/155 fragment ions using 37 most intense peaks

#	Immon.	a	a*	a^0	b	b*	b^0	d	Seq.	v	y	y*	y^0	#
1	**86.10**	**86.10**			114.09				L					11
2	60.04	173.13		155.12	201.12		183.11		S	1109.53	**1141.56**	1124.53	1123.55	10
3	**70.07**	270.18		252.17	298.18		280.17		P	1012.48	1054.53	1037.50	1036.52	9
4	**86.10**	383.27		365.25	411.26		393.25		L	899.40	957.47	940.45	939.46	8
5	30.03	440.29		422.28	468.28		450.27		G		844.39	827.36	826.38	7
6	102.05	569.33		551.32	597.32		579.31		E	713.33	787.37	770.34	769.36	6
7	102.05	698.37		680.36	726.37		708.36		E	584.29	**658.33**	641.30	640.32	5
8	56.05	781.41		763.40	**809.40**		791.39		M	**501.25**	**529.28**	**512.26**	511.27	4
9	129.11	937.51	920.48	919.50	965.51	948.48	947.49	852.45	R	345.15	**446.25**	429.22	**428.24**	3
10	88.04	1052.54	1035.51	1034.53	**1080.53**	1063.51	1062.52	**1008.55**	D	230.12	**290.15**	273.12	272.14	2
11	129.11								R	74.02	**175.12**	158.09		1

MS/MS Fragmentation of **VQPYLDDFQKK**
Found in **gi|4557321**, apolipoprotcin A-I isoform 1 prcproprotcin [Homo sapicns]

Match to Query 24: 1379.683477 from(1380.690753,1+)
From data file DATA.TXT

Click mouse within plot area to zoom in by factor of two about that point
Or, Plot from 0 to 1400 Da

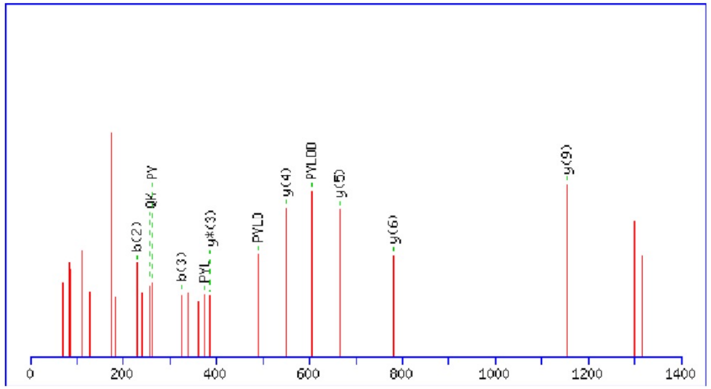

Monoisotopic mass of neutral peptide Mr(calc): 1379.71
Fixed modifications: Carbamidomethyl (C)
Ions Score: 21 **Expect:** 2.1
Matches (Bold Red): 12/137 fragment ions using 19 most intense peaks

#	Immon.	a	a*	a^0	b	b*	b^0	Seq.	y	y*	y^0	#
1	72.08	72.08			100.08			V				11
2	101.07	200.14	183.11		**228.13**	211.11		Q	1281.65	1264.62	1263.64	10
3	70.07	297.19	280.17		**325.19**	308.16		P	**1153.59**	1136.56	1135.58	9
4	136.08	460.26	443.23		488.25	471.22		Y	1056.54	1039.51	1038.53	8
5	86.10	573.34	556.31		601.33	584.31		L	893.47	876.45	875.46	7
6	88.04	688.37	671.34	670.36	716.36	699.33	698.35	D	**780.39**	763.36	762.38	6
7	88.04	803.39	786.37	785.38	831.39	814.36	813.38	D	**665.36**	648.34	647.35	5
8	120.08	950.46	933.44	932.45	978.46	961.43	960.45	F	**550.33**	533.31		4
9	101.07	1078.52	1061.49	1060.51	1106.52	1089.49	1088.50	Q	403.27	**386.24**		3
10	101.11	1206.62	1189.59	1188.60	1234.61	1217.58	1216.60	K	275.21	258.18		2
11	101.11							K	147.11	130.09		1

Spot 2243 analyzed by MS/MS ion search

MS/MS Fragmentation of **DYVSQFEGSALGK**
Found in **gi|4557321**, apolipoprotein A-I isoform 1 preproprotein [Homo sapiens]

Match to Query 25: 1399.633285 from(1400.640561,1+)
From data file DATA.TXT

Click mouse within plot area to zoom in by factor of two about that point
Or, Plot from 0 to 1400 Da

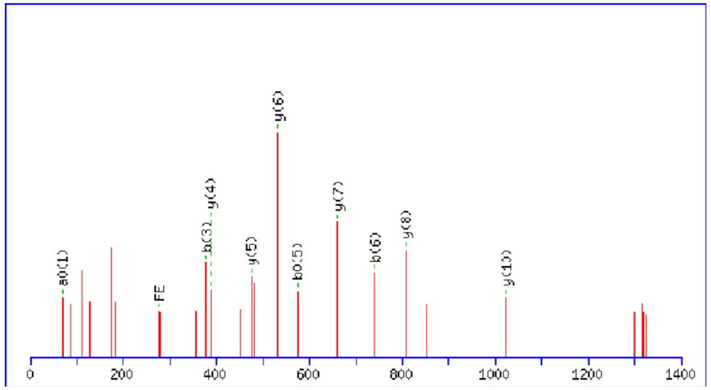

Monoisotopic mass of neutral peptide Mr(calc): 1399.66
Fixed modifications: Carbamidomethyl (C)
Ions Score: 36 **Expect:** 0.045
Matches (Bold Red): 11/190 fragment ions using 18 most intense peaks

#	Immon.	a	a*	a^0	b	b*	b^0	Seq.	y	y*	y^0	#
1	88.04	88.04		**70.03**	116.03		98.02	D				13
2	136.08	251.10		233.09	279.10		261.09	Y	1285.64	1268.62	1267.63	12
3	72.08	350.17		332.16	**378.17**		360.16	V	1122.58	1105.55	1104.57	11
4	60.04	437.20		419.19	465.20		447.19	S	**1023.51**	1006.48	1005.50	10
5	101.07	565.26	548.24	547.25	593.26	576.23	**575.25**	Q	936.48	919.45	918.47	9
6	120.08	712.33	695.30	694.32	**740.32**	723.30	722.31	F	**808.42**	791.39	790.41	8
7	102.05	841.37	824.35	823.36	869.37	852.34	851.36	E	**661.35**	644.32	643.34	7
8	30.03	898.39	881.37	880.38	926.39	909.36	908.38	G	**532.31**	515.28	514.30	6
9	60.04	985.43	968.40	967.42	1013.42	996.39	995.41	S	**475.29**	458.26	457.28	5
10	44.05	1056.46	1039.44	1038.45	1084.46	1067.43	1066.45	A	**388.26**	371.23		4
11	86.10	1169.55	1152.52	1151.54	1197.54	1180.52	1179.53	L	317.22	300.19		3
12	30.03	1226.57	1209.54	1208.56	1254.56	1237.54	1236.55	G	204.13	187.11		2
13	101.11							K	147.11	130.09		1

MS/MS Fragmentation of **VEPLRAELQEGAR**
Found in **gi|4557321**, apolipoprotein A-I isoform 1 preproprotein [Homo sapiens]

Match to Query 27: 1466.767495 from(1467.774771,1+)
From data file DATA.TXT

Click mouse within plot area to zoom in by factor of two about that point
Or, Plot from 0 to 1400 Da

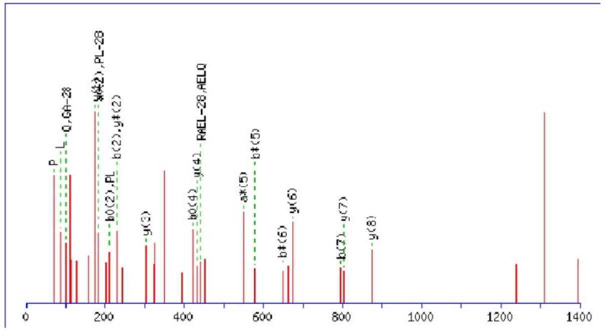

Monoisotopic mass of neutral peptide Mr(calc): 1466.78
Fixed modifications: Carbamidomethyl (C)
Ions Score: 33 **Expect:** 0.11
Matches (Bold Red): 25/204 fragment ions using 27 most intense peaks

#	Immon.	a	a*	a^0	b	b*	b^0	d	Seq.	v	y	y*	y^0	#
1	72.08	72.08			100.08				V					13
2	102.05	201.12		**183.11**	**229.12**		**211.11**		E	1294.69	1368.72	1351.70	1350.71	12
3	**70.07**	298.18		280.17	326.17		308.16		P	1197.63	1239.68	1222.65	1221.67	11
4	**86.10**	411.26		393.25	439.26		**421.24**		L	1084.55	1142.63	1125.60	1124.62	10
5	129.11	567.36	**550.33**	549.35	595.36	**578.33**	577.35	482.30	R	928.45	1029.54	1012.52	1011.53	9
6	44.05	638.40	621.37	620.39	666.39	**649.37**	648.38		A	857.41	**873.44**	856.42	855.43	8
7	102.05	767.44	750.41	749.43	**795.44**	778.41	777.43	709.44	E	728.37	**802.41**	785.38	784.39	7
8	**86.10**	880.53	863.50	862.51	908.52	891.49	890.51	838.48	L	615.28	**673.36**	656.34	655.35	6
9	**101.07**	1008.58	991.56	990.57	1036.58	1019.55	1018.57	951.56	Q	487.23	560.28	543.25	542.27	5
10	102.05	1137.63	1120.60	1119.62	1165.62	1148.59	1147.61	1079.62	E	358.18	**432.22**	415.19	414.21	4
11	30.03	1194.65	1177.62	1176.64	1222.64	1205.62	1204.63		G		**303.18**	286.15		3
12	44.05	1265.68	1248.66	1247.67	1293.68	1276.65	1275.67		A	230.12	246.16	**229.13**		2
13	129.11								R	74.02	**175.12**	158.09		1

Spot 2243 analyzed by MS/MS ion search

Supplementary Figure 4C

MS/MS Fragmentation of **THLAPYSDELRQR**
Found in **gi|4557321**, apolipoprotein A-I isoform 1 preproprotein [Homo sapiens]

Match to Query 28: 1584.779905 from(1585.787181,1+)
From data file DATA.TXT

Click mouse within plot area to zoom in by factor of two about that point
Or, Plot from 0 to 1500 Da

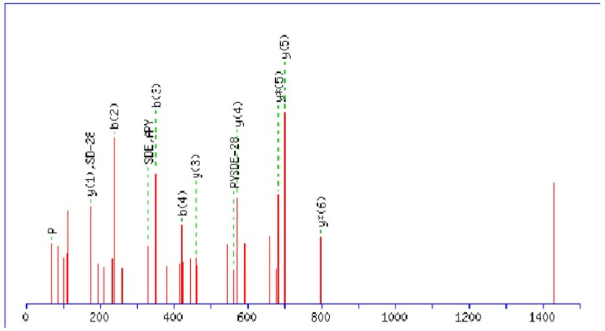

Monoisotopic mass of neutral peptide Mr(calc): 1584.80
Fixed modifications: Carbamidomethyl (C)
Ions Score: 24 **Expect:** 0.94
Matches (Bold Red): 14/186 fragment ions using 16 most intense peaks

#	Immon.	a	a*	a^0	b	b*	b^0	d	Seq.	v	y	y*	y^0	#
1	74.06	74.06		56.05	102.05		84.04		T					13
2	110.07	211.12		193.11	**239.11**		221.10		H	1402.71	1484.76	1467.73	1466.75	12
3	86.10	324.20		306.19	**352.20**		334.19		L	1289.62	1347.70	1330.67	1329.69	11
4	44.05	395.24		377.23	**423.24**		405.22		A	1218.59	1234.62	1217.59	1216.61	10
5	**70.07**	492.29		474.28	520.29		502.28		P	1121.53	1163.58	1146.55	1145.57	9
6	136.08	655.36		637.35	683.35		665.34		Y	958.47	1066.53	1049.50	1048.52	8
7	60.04	742.39		724.38	770.38		752.37		S	871.44	903.46	886.44	885.45	7
8	88.04	857.42		839.40	885.41		867.40		D	756.41	816.43	**799.41**	798.42	6
9	102.05	986.46		968.45	1014.45		996.44		E	627.37	**701.41**	**684.38**	683.39	5
10	86.10	1099.54		1081.53	1127.54		1109.53		L	514.28	**572.36**	555.34		4
11	129.11	1255.64	1238.62	1237.63	1283.64	1266.61	1265.63	1170.58	R	358.18	**459.28**	442.25		3
12	101.07	1383.70	1366.67	1365.69	1411.70	1394.67	1393.69	1326.68	Q	230.12	303.18	286.15		2
13	129.11								R	74.02	**175.12**	158.09		1

MS/MS Fragmentation of **DSGRDYVSQFEGSALGK**
Found in **gi|4557321**, apolipoprotein A-I isoform 1 preproprotein [Homo sapiens]

Match to Query 31: 1814.828542 from(1815.835818,1+)
From data file DATA.TXT

Click mouse within plot area to zoom in by factor of two about that point
Or, Plot from 100 to 1800 Da

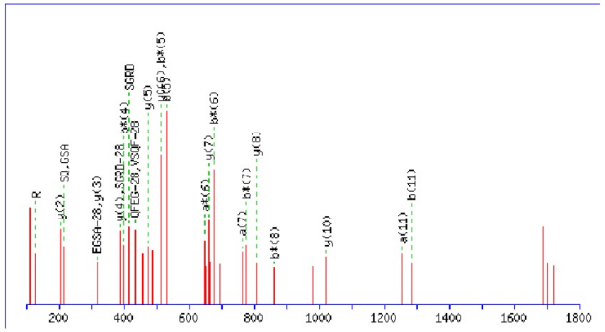

Monoisotopic mass of neutral peptide Mr(calc): 1814.84
Fixed modifications: Carbamidomethyl (C)
Ions Score: 52 **Expect:** 0.00087
Matches (Bold Red): 28/278 fragment ions using 26 most intense peaks

#	Immon.	a	a*	a^0	b	b*	b^0	d	Seq.	v	y	y*	y^0	#
1	88.04	88.04		70.03	116.03		98.02		D					17
2	60.04	175.07		157.06	203.07		185.06		S	1668.80	1700.82	1683.80	1682.81	16
3	30.03	232.09		214.08	260.09		242.08		G		1613.79	1596.77	1595.78	15
4	**129.11**	**388.19**	371.17	370.18	**416.19**	**399.16**	398.18	303.13	R	1455.68	1556.77	1539.74	1538.76	14
5	88.04	503.22	486.19	485.21	**531.22**	**514.19**	513.21	459.23	D		1400.67	1383.64	1382.66	13
6	136.08	666.28	**649.26**	648.27	694.28	**677.25**	676.27		Y		1285.64	1268.62	1267.63	12
7	72.08	**765.35**	748.33	747.34	793.35	**776.32**	775.34	751.34	V		1122.58	1105.55	1104.57	11
8	60.04	852.38	835.36	834.37	880.38	**863.35**	862.37	836.39	S		**1023.51**	1006.48	1005.50	10
9	101.07	980.44	963.42	962.43	1008.44	991.41	990.43	923.42	Q		936.48	919.45	918.47	9
10	120.08	1127.51	1110.49	1109.50	1155.51	1138.48	1137.50		F		**808.42**	791.39	790.41	8
11	102.05	**1256.55**	1239.53	1238.54	**1284.55**	1267.52	1266.54	1198.55	E		**661.35**	644.32	643.34	7
12	30.03	1313.58	1296.55	1295.57	1341.57	1324.54	1323.56		G		532.31	515.28	**514.30**	6
13	60.04	1400.61	1383.58	1382.60	1428.60	1411.58	1410.59	1384.61	S		**475.29**	458.26	457.28	5
14	44.05	1471.64	1454.62	1453.63	1499.64	1482.61	1481.63		A		**388.26**	371.23		4
15	86.10	1584.73	1567.70	1566.72	1612.72	1595.70	1594.71	1542.68	L		**317.22**	300.19		3
16	30.03	1641.75	1624.72	1623.74	1669.75	1652.72	1651.73		G		**204.13**	187.11		2
17	101.11								K		147.11	130.09		1

Spot 2243 analyzed by MS/MS ion search

Supplementary Figure 4D

```
Match to: gi|115298678 Score: 96
complement C3 preproprotein [Homo sapiens]
Found in search of DATA.TXT

Nominal mass (Mr): 188569; Calculated pI value: 6.02
NCBI BLAST search of gi|115298678 against nr
Unformatted sequence string for pasting into other applications

Taxonomy: Homo sapiens

Fixed modifications: Carbamidomethyl (C)
Variable modifications: Oxidation (M)
Cleavage by Trypsin: cuts C-term side of KR unless next residue is P
Sequence Coverage: 2%

Matched peptides shown in Bold Red

   1 MGPTSGPSLL LLLLTHLPLA LGSPMYSIIT PNILRLESEE TMVLEAHDAQ
  51 GDVPVTVTVH DFPGKKLVLS SEKTVLTPAT NHMGNVTFTI PANREFKSEK
 101 GRNKFVTVQA TFGTQVVEKV VLVSLQSGYL FIQTDKTIYT PGSTVLYRIF
 151 TVNHKLLPVG RTVMVNIENP EGIPVKQDSL SSQNQLGVLP LSWDIPELVN
 201 MGQWKIRAYY ENSPQQVFST EFEVKEYVLP SFEVIVEPTE KFYYIYNEKG
 251 LEVTITARFL YGKKVEGTAF VIFGIQDGEQ RISLPESLKR IPIEDGSGEV
 301 VLSRKVLLDG VQNPRAEDLV GKSLYVSATV ILHSGSDMVQ AERSGIPIVT
 351 SPYQIHFTKT PKYFKPGMPF DLMVFVTNPD GSPAYRVPVA VQGEDTVQSL
 401 TQGDGVAKLS INTHPSQKPL SITVRTKKQE LSEAEQATRT MQALPYSTVG
 451 NSNNYLHLSV LRTELRPGET LNVNFLLRMD RAHEAKIRYY TYLIMNKGRL
 501 LKAGRQVREP GQDLVVLPLS ITTDFIPSFR LVAYYTLIGA SGQREVVADS
 551 VWVDVKDSCV GSLVVKSGQS EDRQPVPGQQ MTLKIEGDHG ARVVLVAVDK
 601 GVFVLNKKNK LTQSKIWDVV EKADIGCTPG SGKDYAGVFS DAGLTFTSSS
 651 GQQTAQRAEL QCPQPAARRR RSVQLTEKRM DKVGKYPKEL RKCCEDGMRE
 701 NPMRFSCQRR TRFISLGEAC KKVFLDCCNY ITELRRQHAR ASHLGLARSN
 751 LDEDIIAEEN IVSRSEFPES WLWNVEDLKE PPKNGISTKL MNIFLKDSIT
 801 TWEILAVSMS DKKGICVADP FEVTVMQDFF IDLRLPYSVV RNEQVEIRAV
 851 LYNYRQNQEL KVRVELLHNP AFCSLATTKR RHQQTVTIPP KSSLSVPYVI
 901 VPLKTGLQEV EVKAAVYHHF ISDGVRKSLK VVPEGIRMNK TVAVRTLDPE
 951 RLGREGVQKE DIPPADLSDQ VPDTESETRI LLQGTPVAQM TEDAVDAERL
1001 KHLIVTPSGC GEQNMIGMTP TVIAVHYLDE TEQWEKFGLE KRQGALELIK
1051 KGYTQQLAFR QPSSAFAAFV KRAPSTWLTA YVVKVFSLAV NLIAIDSQVL
1101 CGAVKWLILE KQKPDGVFQE DAPVIHQEMI GGLRNNNEKD MALTAFVLIS
1151 LQEAKDICEE QVNSLPGSIT KAGDFLEANY MNLQRSYTVA IAGYALAQMG
1201 RLKGPLLNKF LTTAKDKNRW EDPGKQLYNV EATSYALLAL LQLKDFDFVP
1251 PVVRWLNEQR YYGGGYGSTQ ATFMVFQALA QYQKDAPDHQ ELNLDVSLQL
1301 PSRSSKITHR IHWESASLLR SEETKENEGF TVTAEGKGQG TLSVVTMYHA
1351 KAKDQLTCNK FDLKVTIKPA PETEKRPQDA KNTMILEICT RYRGDQDATM
1401 SILDISMMTG FAPDTDDLKQ LANGVDRYIS KYELDKAFSD RNTLIIYLDK
1451 VSHSEDDCLA FKVHQYFNVE LIQPGAVKVY AYYNLEESCT RFYHPEKEDG
1501 KLNKLCRDEL CRCAEENCFI QKSDDKVTLE ERLDKACEPG VDYVYKTRLV
1551 KVQLSNDFDE YIMAIEQTIK SGSDEVQVGQ QRTFISPIKC REALKLEEKK
1601 HYLMWGLSSD FWGEKPNLSY IIGKDTWVEH WPEEDECQDE ENQKQCQDLG
1651 AFTESMVVFG CPN
```

MS/MS Fragmentation of **FGLEKR**
Found in **gi|115298678**, complement C3 preproprotein [Homo sapiens]

Match to Query 1: 748.463501 from(749.470777,1+)
From data file DATA.TXT

Click mouse within plot area to zoom in by factor of two about that point
Or, Plot from 50 to 750 Da

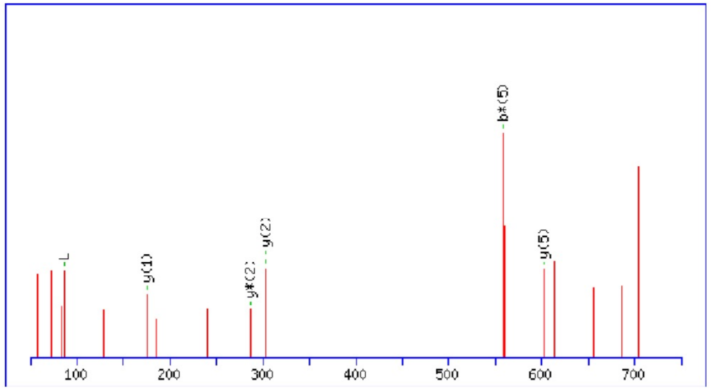

Monoisotopic mass of neutral peptide Mr(calc): 748.42
Fixed modifications: Carbamidomethyl (C)
Ions Score: 13 **Expect:** 19
Matches (Bold Red): 6/51 fragment ions using 15 most intense peaks

#	Immon.	a	a*	a^0	b	b*	b^0	Seq.	v	y	y*	y^0	#
1	120.08	120.08			148.08			F					6
2	30.03	177.10			205.10			G		**602.36**	585.34	584.35	5
3	**86.10**	290.19			318.18			L	487.26	545.34	528.31	527.33	4
4	102.05	419.23		401.22	447.22		429.21	E	358.22	432.26	415.23	414.25	3
5	101.11	547.32	530.30	529.31	575.32	**558.29**	557.31	K	230.12	**303.21**	**286.19**		2
6	129.11							R	74.02	**175.12**	158.09		1

Spot 1084 analyzed by MS/MS ion search

Supplementary Figure 4D

MS/MS Fragmentation of **RQGALELIK**
Found in **gi|115298678**, complement C3 preproprotein [Homo sapiens]

Match to Query 6: 1026.633425 from(1027.640701,1+)
From data file DATA.TXT

Click mouse within plot area to zoom in by factor of two about that point
Or, Plot from 50 to 1000 Da

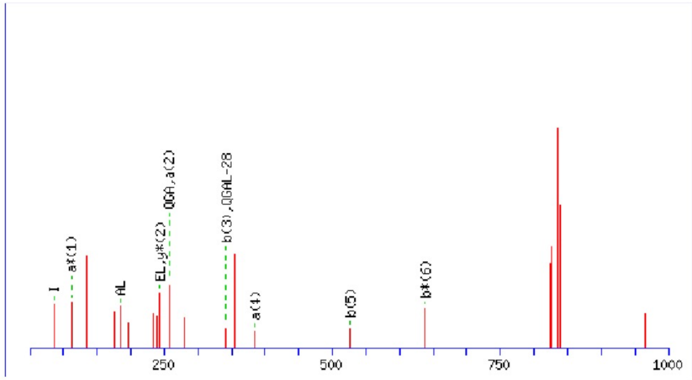

Monoisotopic mass of neutral peptide Mr(calc): 1026.62
Fixed modifications: Carbamidomethyl (C)
Ions Score: 5 **Expect:** 59
Matches (Bold Red): 15/116 fragment ions using 19 most intense peaks

#	Immon.	a	a*	a^0	b	b*	b^0	d	d'	Seq.	y	y*	y^0	#
1	129.11	129.11	**112.09**		157.11	140.08		44.05		R				9
2	101.07	**257.17**	240.15		285.17	268.14		200.15		Q	871.52	854.50	853.51	8
3	30.03	314.19	297.17		**342.19**	325.16				G	743.47	726.44	725.46	7
4	44.05	**385.23**	368.20		413.23	396.20				A	686.44	669.42	668.43	6
5	**86.10**	498.31	481.29		**526.31**	509.28		456.27		L	615.41	598.38	597.40	5
6	102.05	627.36	610.33	609.35	655.35	**638.33**	637.34	569.35		E	502.32	485.30	484.31	4
7	**86.10**	740.44	723.41	722.43	768.44	751.41	750.43	698.39		L	373.28	356.25		3
8	**86.10**	853.53	836.50	835.51	881.52	864.49	863.51	825.49	839.51	I	260.20	**243.17**		2
9	101.11									K	147.11	130.09		1

MS/MS Fragmentation of **GYTQQLAFR**
Found in **gi|115298678**, complement C3 preproprotein [Homo sapiens]

Match to Query 8: 1082.553217 from(1083.560493,1+)
From data file DATA.TXT

Click mouse within plot area to zoom in by factor of two about that point
Or, Plot from 50 to 1050 Da

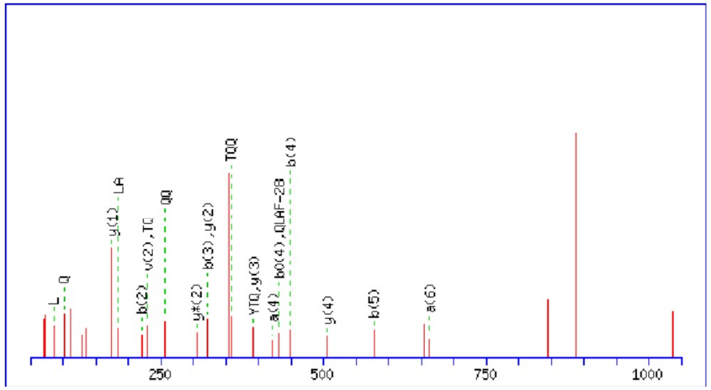

Monoisotopic mass of neutral peptide Mr(calc): 1082.55
Fixed modifications: Carbamidomethyl (C)
Ions Score: 27 **Expect:** 0.54
Matches (Bold Red): 22/112 fragment ions using 25 most intense peaks

#	Immon.	a	a*	a^0	b	b*	b^0	Seq.	v	y	y*	y^0	#
1	30.03	30.03			58.03			G					9
2	136.08	193.10			**221.09**			Y	918.48	1026.54	1009.51	1008.53	8
3	74.06	294.14		276.13	**322.14**		304.13	T	817.43	863.47	846.45	845.46	7
4	**101.07**	**422.20**	405.18	404.19	**450.20**	433.17	**432.19**	Q	689.37	762.43	745.40		6
5	**101.07**	550.26	533.24	532.25	**578.26**	561.23	560.25	Q	561.31	634.37	617.34		5
6	**86.10**	**663.35**	646.32	645.34	691.34	674.31	673.33	L	448.23	**506.31**	489.28		4
7	44.05	734.38	717.36	716.37	762.38	745.35	744.37	A	377.19	**393.22**	376.20		3
8	120.08	881.45	864.43	863.44	909.45	892.42	891.44	F	**230.12**	**322.19**	**305.16**		2
9	129.11							R	74.02	**175.12**	158.09		1

Spot 1084 analyzed by MS/MS ion search

Supplementary Figure 4D

MS/MS Fragmentation of **AGDFLEANYMNLQR**
Found in **gi|115298678**, complement C3 preproprotein [Homo sapiens]

Match to Query 13: 1656.765699 from(1657.772975,1+)
From data file DATA.TXT

Click mouse within plot area to zoom in by factor of two about that point
Or, Plot from 0 to 1600 Da

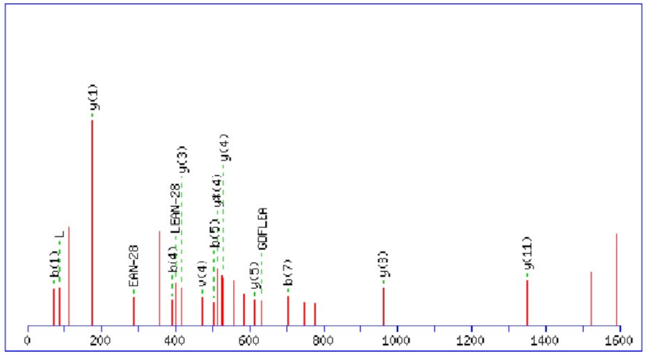

Monoisotopic mass of neutral peptide Mr(calc): 1656.76
Fixed modifications: Carbamidomethyl (C)
Variable modifications:
M10 : Oxidation (M)
Ions Score: 51 **Expect:** 0.0013
Matches (Bold Red): 18/204 fragment ions using 25 most intense peaks

#	Immon.	a	a*	a^0	b	b*	b^0	Seq.	v	y	y*	y^0	#
1	44.05	44.05			**72.04**			A					14
2	30.03	101.07			129.07			G		1522.73	1505.70	1504.72	13
3	88.04	216.10		198.09	244.09		226.08	D	1405.69	1465.71	1448.68	1447.70	12
4	120.08	363.17		345.16	**391.16**		373.15	F	1258.62	**1350.68**	1333.65	1332.67	11
5	**86.10**	476.25		458.24	**504.25**		486.23	L	1145.53	1203.61	1186.59	1185.60	10
6	102.05	605.29		587.28	**633.29**		615.28	E	1016.49	1090.53	1073.50	1072.52	9
7	44.05	676.33		658.32	**704.32**		686.31	A	945.45	**961.48**	944.46		8
8	87.06	790.37	773.35	772.36	818.37	801.34	800.36	N	831.41	890.45	873.42		7
9	136.08	953.44	936.41	935.43	981.43	964.40	963.42	Y	668.35	776.40	759.38		6
10	56.05	1036.47	1019.45	1018.46	1064.47	1047.44	1046.46	M	585.31	**613.34**	596.32		5
11	87.06	1150.52	1133.49	1132.51	1178.51	1161.48	1160.50	N	**471.27**	**530.30**	**513.28**		4
12	**86.10**	1263.60	1246.57	1245.59	1291.60	1274.57	1273.58	L	358.18	**416.26**	399.24		3
13	101.07	1391.66	1374.63	1373.65	1419.65	1402.63	1401.64	Q	230.12	303.18	286.15		2
14	129.11							R	74.02	**175.12**	158.09		1

Spot 1084 analyzed by MS/MS ion search

Supplementary Figure 4E

Match to: **gi|4504893** Score: **430**
kininogen-1 isoform 2 precursor [Homo sapiens]
Found in search of DATA.TXT

Nominal mass (M_r): **48936**; Calculated pI value: **6.29**
NCBI BLAST search of gi|4504893 against nr
Unformatted sequence string for pasting into other applications

Taxonomy: Homo sapiens

Fixed modifications: Carbamidomethyl (C)
Variable modifications: Oxidation (M)
Cleavage by Trypsin: cuts C-term side of KR unless next residue is P
Sequence Coverage: **20%**

Matched peptides shown in **Bold Red**

1 MKLITILFLC SRLLLSLTQE SQSEEIDCND KDLFKAVDAA LK**KYNSQNQS**
51 **NNQFVLYR**IT EATKTVGSDT FYSFKYEIKE GDCPVQSGKT WQDCEYKDAA
101 K**AATGECTAT VGKR**SSTKFS VATQTCQITP AEGPVVTAQY DCLGCVHPIS
151 TQSPDLEPIL RHGIQYFNNN TQHSSLFMLN EVKRAQR**QVV AGLNFR**ITYS
201 IVQTNCSKEN FLFLTPDCKS LWNGDTGECT DNAYIDIQLR IASFSQNCDI
251 YPGKDFVQPP TK**ICVGCPR**D IPTNSPELEE TLTHTITKLN AENNATFYFK
301 IDNVKKARVQ VVAGK**KYFID FVAR**ETTCSK ESNEELTESC ETKKLGQSLD
351 CNAEVYVVPW EKKIYPTVNC QPLGMISLMK **RPPGFSPFR**S SR**IGEIKEET**
401 **TSHLR**SCEYK GRPPK**AGAEP ASEREVS**

MS/MS Fragmentation of **ICVGCPR**
Found in **gi|4504893**, kininogen-1 isoform 2 precursor [Homo sapiens]

Match to Query 8: 860.387750 from(861.395026,1+)
From data file DATA.TXT

Click mouse within plot area to zoom in by factor of two about that point
Or, Plot from 50 to 850 Da

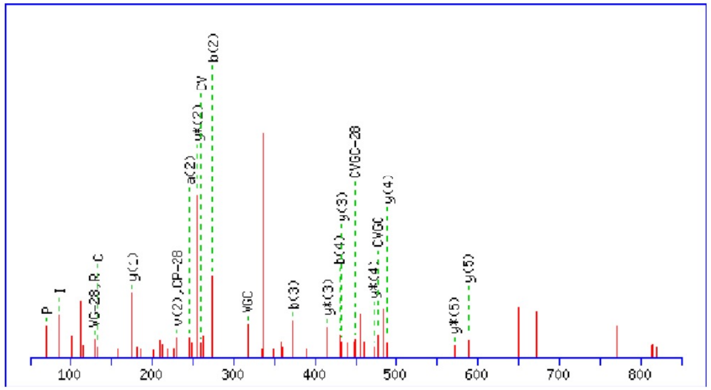

Monoisotopic mass of neutral peptide Mr(calc): 860.40
Fixed modifications: Carbamidomethyl (C)
Ions Score: 17 **Expect:** 7.9
Matches (Bold Red): 26/56 fragment ions using 40 most intense peaks

#	Immon.	a	b	Seq.	y	y	y*	#
1	**86.10**	**86.10**	114.09	I				7
2	**133.04**	**246.13**	**274.12**	C	643.30	748.32	731.30	6
3	72.08	345.20	**373.19**	V	544.23	**588.29**	**571.27**	5
4	30.03	402.22	**430.21**	G		**489.22**	**472.20**	4
5	**133.04**	562.25	590.24	C	327.18	**432.20**	**415.18**	3
6	**70.07**	659.30	687.30	P	**230.12**	272.17	**255.15**	2
7	**129.11**			R	74.02	**175.12**	158.09	1

Spot 528 analyzed by MS/MS ion search

MS/MS Fragmentation of **QVVAGLNFR**
Found in **gi|4504893**, kininogen-1 isoform 2 precursor [Homo sapiens]

Match to Query 15: 1002.547453 from(1003.554729,1+)
From data file DATA.TXT

Click mouse within plot area to zoom in by factor of two about that point
Or, Plot from 50 to 1000 Da

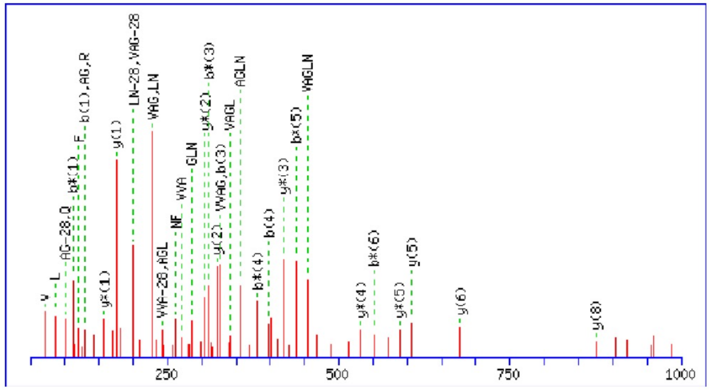

Monoisotopic mass of neutral peptide Mr(calc): 1002.56
Fixed modifications: Carbamidomethyl (C)
Ions Score: 37 **Expect:** 0.077
Matches (Bold Red): 43/105 fragment ions using 42 most intense peaks

#	Immon.	a	a*	b	b*	Seq.	v	y	y*	#
1	**101.07**	**101.07**	84.04	**129.07**	**112.04**	Q				9
2	**72.08**	**200.14**	183.11	**228.13**	211.11	V	831.45	**875.51**	858.48	8
3	**72.08**	299.21	282.18	**327.20**	**310.18**	V	732.38	776.44	759.41	7
4	44.05	370.24	353.22	**398.24**	**381.21**	A	661.34	**677.37**	660.35	6
5	30.03	427.27	410.24	**455.26**	**438.23**	G		**606.34**	**589.31**	5
6	**86.10**	540.35	523.32	568.35	**551.32**	L	491.24	549.31	**532.29**	4
7	87.06	654.39	637.37	682.39	665.36	N	377.19	436.23	**419.20**	3
8	**120.08**	801.46	784.44	829.46	812.43	F	230.12	**322.19**	305.16	2
9	**129.11**					R	74.02	**175.12**	**158.09**	1

Supplementary Figure 4E

MS/MS Fragmentation of **YFIDFVAR**
Found in **gi|4504893**, kininogen-1 isoform 2 precursor [Homo sapiens]

Match to Query 18: 1029.514608 from(1030.521884,1+)
From data file DATA.TXT

Click mouse within plot area to zoom in by factor of two about that point
Or, Plot from 50 to 1000 Da

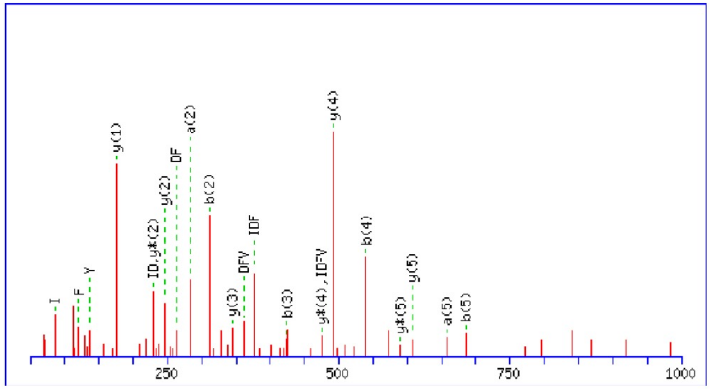

Monoisotopic mass of neutral peptide Mr(calc): 1029.53
Fixed modifications: Carbamidomethyl (C)
Ions Score: 34 **Expect:** 0.13
Matches (Bold Red): 25/84 fragment ions using 31 most intense peaks

#	Immon.	a	a^0	b	b^0	Seq.	y	y*	y^0	#
1	**136.08**	**136.08**		164.07		Y				8
2	**120.08**	**283.14**		**311.14**		F	867.47	850.45	849.46	7
3	**86.10**	396.23		**424.22**		I	720.40	703.38	702.39	6
4	88.04	511.26	493.24	**539.25**	521.24	D	**607.32**	**590.29**	589.31	5
5	**120.08**	**658.32**	640.31	**686.32**	668.31	F	**492.29**	**475.27**		4
6	72.08	757.39	739.38	785.39	767.38	V	**345.22**	328.20		3
7	44.05	828.43	810.42	856.42	838.41	A	**246.16**	**229.13**		2
8	129.11					R	**175.12**	158.09		1

Note: the printed table also has a "v" column between Seq. and y: 775.41 (row 2), 662.33 (row 3), 547.30 (row 4), 400.23 (row 5), 301.16 (row 6), 230.12 (row 7), 74.02 (row 8).

MS/MS Fragmentation of **RPPGFSPFR**
Found in **gi|4504893**, kininogen-1 isoform 2 precursor [Homo sapiens]

Match to Query 20: 1059.547165 from(1060.554441,1+)
From data file DATA.TXT

Click mouse within plot area to zoom in by factor of two about that point
Or, Plot from 50 to 1050 Da

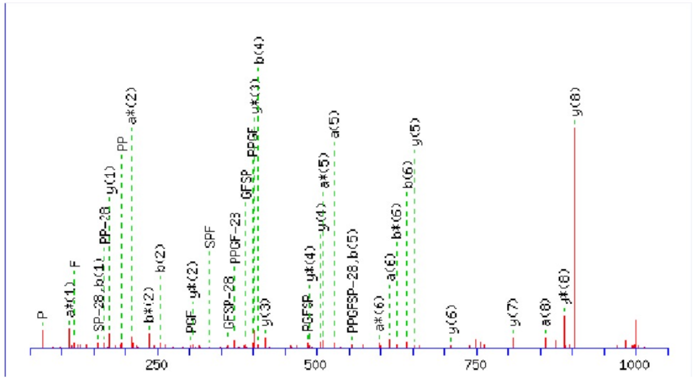

Monoisotopic mass of neutral peptide Mr(calc): 1059.56
Fixed modifications: Carbamidomethyl (C)
Ions Score: 48 **Expect:** 0.0068
Matches (Bold Red): 45/120 fragment ions using 56 most intense peaks

#	Immon.	a	a*	a^0	b	b*	b^0	d	Seq.	v	y	y*	y^0	#
1	129.11	129.11	**112.09**		**157.11**	140.08		44.05	R					9
2	**70.07**	226.17	**209.14**		**254.16**	**237.13**		200.15	P	862.42	**904.47**	**887.44**	886.46	8
3	**70.07**	323.22	306.19		351.21	334.19		297.20	P	765.37	**807.41**	790.39	789.40	7
4	30.03	380.24	363.21		**408.24**	391.21			G		**710.36**	693.34	692.35	6
5	**120.08**	**527.31**	**510.28**		**555.30**	538.28			F	561.28	**653.34**	636.31	635.33	5
6	60.04	**614.34**	**597.31**	596.33	**642.34**	**625.31**	624.33	598.35	S	474.25	**506.27**	**489.25**	488.26	4
7	**70.07**	711.39	694.37	693.38	739.39	722.36	721.38	685.38	P	377.19	**419.24**	**402.21**		3
8	**120.08**	**858.46**	841.44	840.45	886.46	869.43	868.45		F	230.12	322.19	**305.16**		2
9	129.11								R	74.02	**175.12**	158.09		1

Spot 528 analyzed by MS/MS ion search

MS/MS Fragmentation of **KYFIDFVAR**
Found in **gi|4504893**, kininogen-1 isoform 2 precursor [Homo sapiens]

Match to Query 25: 1157.611048 from(1158.618324,1+)
From data file DATA.TXT

Click mouse within plot area to zoom in by factor of two about that point
Or, Plot from 0 to 1100 Da

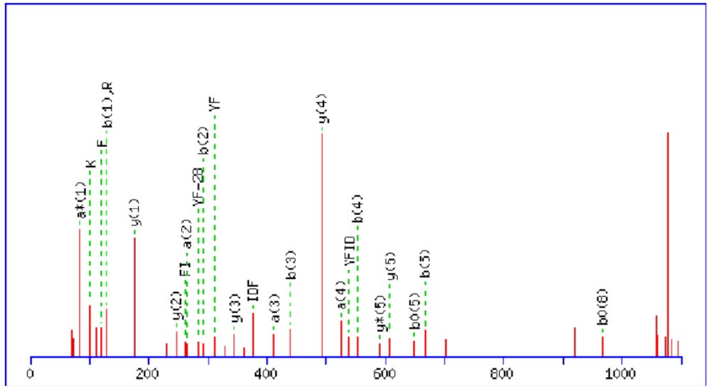

Monoisotopic mass of neutral peptide Mr(calc): 1157.62
Fixed modifications: Carbamidomethyl (C)
Ions Score: 43 **Expect:** 0.019
Matches (Bold Red): 28/115 fragment ions using 33 most intense peaks

#	Immon.	a	a*	a^0	b	b*	b^0	Seq.	v	y	y*	y^0	#
1	**101.11**	**101.11**	**84.08**		**129.10**	112.08		K					9
2	136.08	**264.17**	247.14		**292.17**	275.14		Y	922.48	1030.54	1013.51	1012.53	8
3	**120.08**	**411.24**	394.21		**439.23**	422.21		F	775.41	867.47	850.45	849.46	7
4	86.10	**524.32**	507.30		**552.32**	535.29		I	662.33	720.40	703.38	702.39	6
5	88.04	639.35	622.32	621.34	**667.34**	650.32	**649.33**	D	547.30	**607.32**	**590.29**	589.31	5
6	**120.08**	786.42	769.39	768.41	814.41	797.39	796.40	F	400.23	**492.29**	475.27		4
7	72.08	885.49	868.46	867.48	913.48	896.46	895.47	V	301.16	**345.22**	328.20		3
8	44.05	956.52	939.50	938.51	984.52	967.49	**966.51**	A	230.12	**246.16**	229.13		2
9	**129.11**							R	74.02	**175.12**	158.09		1

MS/MS Fragmentation of **AGAEPASEREVS**
Found in **gi|4504893**, kininogen-1 isoform 2 precursor [Homo sapiens]

Match to Query 26: 1201.548632 from(1202.555908,1+)
From data file DATA.TXT

Click mouse within plot area to zoom in by factor of two about that point
Or, Plot from 0 to 1200 Da

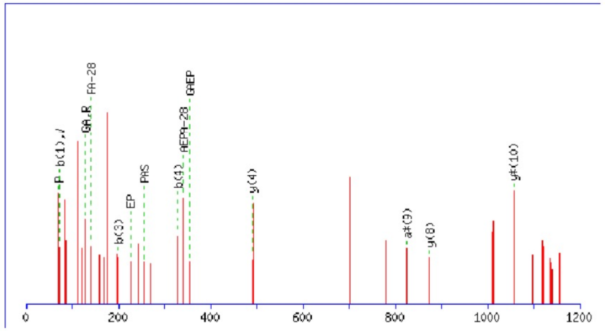

Monoisotopic mass of neutral peptide Mr(calc): 1201.56
Fixed modifications: Carbamidomethyl (C)
Ions Score: 11 **Expect:** 20
Matches (Bold Red): 17/168 fragment ions using 33 most intense peaks

#	Immon.	a	a*	a^0	b	b*	b^0	d	Seq.	v	y	y*	y^0	#
1	44.05	44.05			**72.04**				A					12
2	30.03	101.07			**129.07**				G		1131.53	1114.50	1113.52	11
3	44.05	172.11			**200.10**				A	1058.47	1074.51	**1057.48**	1056.50	10
4	102.05	301.15		283.14	**329.15**		311.13		E	929.43	1003.47	986.44	985.46	9
5	**70.07**	398.20		380.19	426.20		408.19		P	832.38	**874.43**	857.40	856.42	8
6	44.05	469.24		451.23	497.24		479.22		A	761.34	777.37	760.35	759.36	7
7	60.04	556.27		538.26	584.27		566.26		S	674.31	706.34	689.31	688.33	6
8	102.05	685.32		667.30	713.31		695.30		E	545.27	619.30	602.28	601.29	5
9	**129.11**	841.42	**824.39**	823.41	869.41	852.38	851.40	756.35	R	389.17	**490.26**	473.24	472.25	4
10	102.05	970.46	953.43	952.45	998.45	981.43	980.44	912.45	E		334.16		316.15	3
11	**72.08**	1069.53	1052.50	1051.52	1097.52	1080.50	1079.51	1055.51	V		205.12		187.11	2
12	60.04								S		106.05		88.04	1

Spot 528 analyzed by MS/MS ion search

Supplementary Figure 4E

MS/MS Fragmentation of **AATGECTATVGKR**
Found in **gi|4504893**, kininogen-1 isoform 2 precursor [Homo sapiens]

Match to Query 29: 1320.633407 from(1321.640683,1+)
From data file DATA.TXT

Click mouse within plot area to zoom in by factor of two about that point
Or, Plot from 0 to 1300 Da

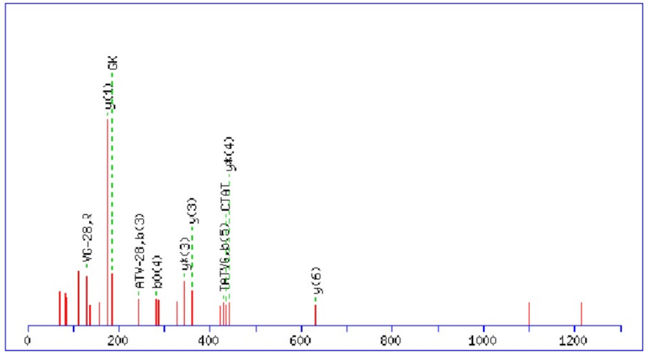

Monoisotopic mass of neutral peptide Mr(calc): 1320.65
Fixed modifications: Carbamidomethyl (C)
Ions Score: 21 **Expect:** 2.4
Matches (Bold Red): 15/187 fragment ions using 15 most intense peaks

#	Immon.	a	a*	a^0	b	b*	b^0	Seq.	v	y	y*	y^0	#
1	44.05	44.05			72.04			A					13
2	44.05	115.09			143.08			A	1234.58	1250.62	1233.59	1232.61	12
3	74.06	216.13		198.12	**244.13**		226.12	T	1133.54	1179.58	1162.55	1161.57	11
4	30.03	273.16		255.15	301.15		**283.14**	G		1078.53	1061.50	1060.52	10
5	102.05	402.20		384.19	**430.19**		412.18	E	947.47	1021.51	1004.48	1003.50	9
6	133.04	562.23		544.22	590.22		572.21	C	787.44	892.47	875.44	874.46	8
7	74.06	663.28		645.27	691.27		673.26	T	686.39	732.44	715.41	714.43	7
8	44.05	734.31		716.30	762.31		744.30	A	615.36	**631.39**	614.36	613.38	6
9	74.06	835.36		817.35	863.36		845.35	T	514.31	560.35	543.32	542.34	5
10	72.08	934.43		916.42	962.42		944.41	V	415.24	459.30	**442.28**		4
11	30.03	991.45		973.44	1019.45		1001.44	G		**360.24**	**343.21**		3
12	101.11	1119.55	1102.52	1101.54	1147.54	1130.51	1129.53	K	230.12	303.21	286.19		2
13	**129.11**							R	74.02	**175.12**	158.09		1

MS/MS Fragmentation of **IGEIKEETTSHLR**
Found in **gi|4504893**, kininogen-1 isoform 2 precursor [Homo sapiens]

Match to Query 33: 1511.789110 from(1512.796386,1+)
From data file DATA.TXT

Click mouse within plot area to zoom in by factor of two about that point
Or, Plot from 0 to 1500 Da

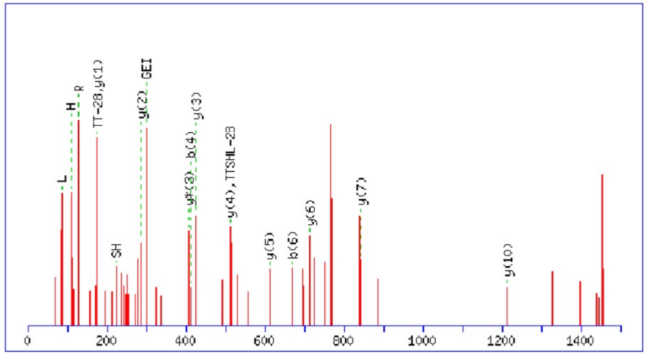

Monoisotopic mass of neutral peptide Mr(calc): 1511.79
Fixed modifications: Carbamidomethyl (C)
Ions Score: 54 **Expect:** 0.00085
Matches (Bold Red): 22/194 fragment ions using 29 most intense peaks

#	Immon.	a	a*	a^0	b	b*	b^0	Seq.	v	y	y*	y^0	#
1	**86.10**	**86.10**			114.09			I					13
2	30.03	143.12			171.11			G		1399.72	1382.69	1381.71	12
3	102.05	272.16		254.15	**300.16**		282.14	E	1268.66	1342.70	1325.67	1324.69	11
4	**86.10**	385.24		367.23	**413.24**		395.23	I	1155.58	**1213.65**	1196.63	1195.64	10
5	101.11	513.34	496.31	495.33	541.33	524.31	523.32	K	1027.48	1100.57	1083.54	1082.56	9
6	102.05	642.38	625.36	624.37	**670.38**	653.35	652.37	E	898.44	972.47	955.45	954.46	8
7	102.05	771.42	754.40	753.41	799.42	782.39	781.41	E	769.40	**843.43**	826.41	825.42	7
8	74.06	872.47	855.45	854.46	900.47	883.44	882.46	T	668.35	**714.39**	697.36	696.38	6
9	74.06	973.52	956.49	955.51	1001.51	984.49	983.50	T	567.30	**613.34**	596.32	595.33	5
10	60.04	1060.55	1043.53	1042.54	1088.55	1071.52	1070.54	S	480.27	**512.29**	495.27	494.28	4
11	**110.07**	1197.61	1180.58	1179.60	1225.61	1208.58	1207.60	H	343.21	**425.26**	**408.24**		3
12	**86.10**	1310.70	1293.67	1292.68	1338.69	1321.66	1320.68	L	230.12	**288.20**	271.18		2
13	**129.11**							R	74.02	**175.12**	158.09		1

Spot 528 analyzed by MS/MS ion search

Supplementary Figure 4E

MS/MS Fragmentation of **YNSQNQSNNQFVLYR**
Found in **gi|4504893**, kininogen-1 isoform 2 precursor [Homo sapiens]

Match to Query 37: 1873.839898 from(1874.847174,1+)
From data file DATA.TXT

Click mouse within plot area to zoom in by factor of two about that point
Or, Plot from 0 to 1600 Da

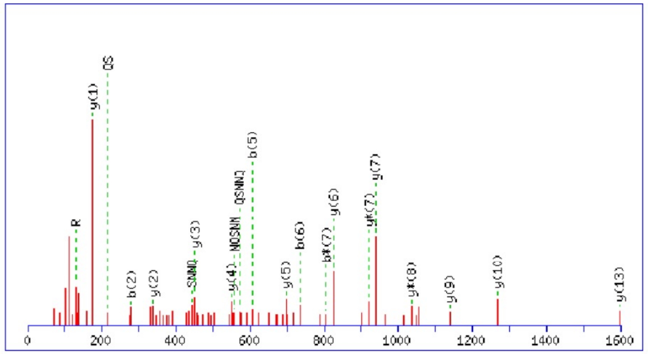

Monoisotopic mass of neutral peptide Mr(calc): 1873.87
Fixed modifications: Carbamidomethyl (C)
Ions Score: 95 **Expect:** 3.1e-008
Matches (Bold Red): 27/237 fragment ions using 23 most intense peaks

#	Immon.	a	a*	a^0	b	b*	b^0	Seq.	v	y	y*	y^0	#
1	136.08	136.08			164.07			Y					15
2	87.06	250.12	233.09		**278.11**	261.09		N	1652.78	1711.81	1694.79	1693.80	14
3	60.04	337.15	320.12	319.14	365.15	348.12	347.14	S	1565.75	**1597.77**	1580.75	1579.76	13
4	101.07	465.21	448.18	447.20	493.20	476.18	475.19	Q	1437.69	1510.74	1493.71	1492.73	12
5	87.06	579.25	562.23	561.24	**607.25**	590.22	589.24	N	1323.64	1382.68	1365.65	1364.67	11
6	101.07	707.31	690.28	689.30	**735.31**	718.28	717.30	Q	1195.59	**1268.64**	1251.61	1250.63	10
7	60.04	794.34	777.32	776.33	822.34	**805.31**	804.33	S	1108.55	**1140.58**	1123.55	1122.57	9
8	87.06	908.39	891.36	890.38	936.38	919.35	918.37	N	994.51	1053.55	**1036.52**		8
9	87.06	1022.43	1005.40	1004.42	1050.42	1033.40	1032.41	N	880.47	**939.50**	**922.48**		7
10	101.07	1150.49	1133.46	1132.48	1178.48	1161.46	1160.47	Q	752.41	**825.46**	808.44		6
11	120.08	1297.56	1280.53	1279.55	1325.55	1308.52	1307.54	F	605.34	**697.40**	680.38		5
12	72.08	1396.62	1379.60	1378.61	1424.62	1407.59	1406.61	V	506.27	**550.33**	533.31		4
13	86.10	1509.71	1492.68	1491.70	1537.70	1520.68	1519.69	L	393.19	**451.27**	434.24		3
14	136.08	1672.77	1655.74	1654.76	1700.77	1683.74	1682.76	Y	230.12	**338.18**	321.16		2
15	**129.11**							R	74.02	**175.12**	158.09		1

MS/MS Fragmentation of **KYNSQNQSNNQFVLYR**
Found in **gi|4504893**, kininogen-1 isoform 2 precursor [Homo sapiens]

Match to Query 38: 2001.937047 from(2002.944323,1+)
From data file DATA.TXT

Click mouse within plot area to zoom in by factor of two about that point
Or, Plot from 0 to 1900 Da

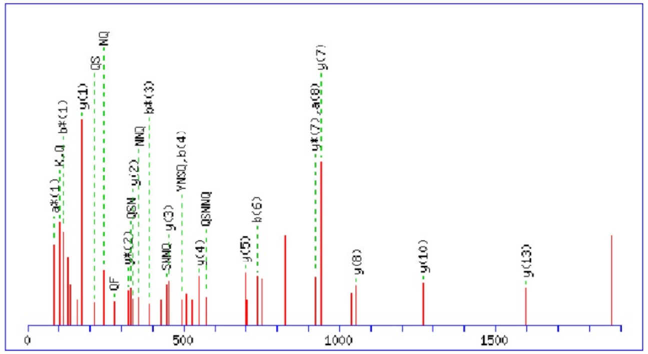

Monoisotopic mass of neutral peptide Mr(calc): 2001.97
Fixed modifications: Carbamidomethyl (C)
Ions Score: 70 **Expect:** 1.4e-005
Matches (Bold Red): 41/256 fragment ions using 31 most intense peaks

#	Immon.	a	a*	a^0	b	b*	b^0	Seq.	v	y	y*	y^0	#
1	**101.11**	**101.11**	**84.08**		129.10	**112.08**		K					16
2	136.08	264.17	247.14		292.17	275.14		Y	1766.82	1874.88	1857.85	1856.87	15
3	87.06	378.21	361.19		406.21	**389.18**		N	1652.78	1711.81	1694.79	1693.80	14
4	60.04	465.25	448.22	447.24	**493.24**	476.21	475.23	S	1565.75	**1597.77**	1580.75	1579.76	13
5	**101.07**	593.30	576.28	575.29	621.30	604.27	603.29	Q	1437.69	1510.74	1493.71	1492.73	12
6	87.06	707.35	690.32	689.34	**735.34**	718.32	717.33	N	1323.64	1382.68	1365.65	1364.67	11
7	**101.07**	835.41	818.38	817.40	863.40	846.37	845.39	Q	1195.59	**1268.64**	1251.61	1250.63	10
8	60.04	**922.44**	905.41	904.43	950.43	933.41	932.42	S	1108.55	1140.58	1123.55	1122.57	9
9	87.06	1036.48	1019.45	1018.47	1064.48	1047.45	1046.47	N	994.51	**1053.55**	1036.52		8
10	87.06	1150.52	1133.50	1132.51	1178.52	1161.49	1160.51	N	880.47	**939.50**	**922.48**		7
11	**101.07**	1278.58	1261.56	1260.57	1306.58	1289.55	1288.57	Q	752.41	825.46	808.44		6
12	120.08	1425.65	1408.62	1407.64	1453.65	1436.62	1435.63	F	605.34	**697.40**	680.38		5
13	72.08	1524.72	1507.69	1506.71	1552.71	1535.69	1534.70	V	506.27	**550.33**	533.31		4
14	86.10	1637.80	1620.78	1619.79	1665.80	1648.77	1647.79	L	393.19	**451.27**	434.24		3
15	136.08	1800.87	1783.84	1782.86	1828.86	1811.83	1810.85	Y	230.12	**338.18**	**321.16**		2
16	129.11							R	74.02	**175.12**	158.09		1

Spot 528 analyzed by MS/MS ion search

Supplementary Figure 4E

Match to: **gi|88853069** Score: **173**
vitronectin precursor [Homo sapiens]
Found in search of DATA.TXT

Nominal mass (M_r): **55069**; Calculated pI value: **5.55**
NCBI BLAST search of gi|88853069 against nr
Unformatted sequence string for pasting into other applications

Taxonomy: Homo sapiens

Fixed modifications: Carbamidomethyl (C)
Variable modifications: Oxidation (M)
Cleavage by Trypsin: cuts C-term side of KR unless next residue is P
Sequence Coverage: **14%**

Matched peptides shown in **Bold Red**

```
  1 MAPLRPLLIL ALLAWVALAD QESCKGRCTE GFNVDKKCQC DELCSYYQSC
 51 CTDYTAECKP QVTRGDVFTM PEDEYTVYDD GEEKNNATVH EQVGGPSLTS
101 DLQAQSKGNP EQTPVLKPEE EAPAPEVGAS KPEGIDSRPE TLHPGRPQPP
151 AEEELCSGKP FDAFTDLKNG SLFAFRGQYC YELDEKAVRP GYPKLIRDVW
201 GIEGPIDAAF TRINCQGKTY LFKGSQYWRF EDGVLDPDYP RNISDGFDGI
251 PDNVDAALAL PAHSYSGRER VYFFKGKQYW EYQFQHQPSQ EECEGSSLSA
301 VFEHFAMMQR DSWEDIFELL FWGRTSAGTR QPQFISRDWH GVPGQVDAAM
351 AGRIYISGMA PRPSLAKKQR FRHRNRKGYR SQRGHSRGRN QNSRRPSRAT
401 WLSLFSSEES NLGANNYDDY RMDWLVPATC EPIQSVFFFS GDKYYRVNLR
451 TRRVDTVDPP YPRSIAQYWL GCPAPGHL
```

MS/MS Fragmentation of **VYFFK**
Found in **gi|88853069**, vitronectin precursor [Homo sapiens]

Match to Query 1: 702.357470 from(703.364746,1+)
From data file DATA.TXT

Click mouse within plot area to zoom in by factor of two about that point
Or, Plot from 100 to 700 Da

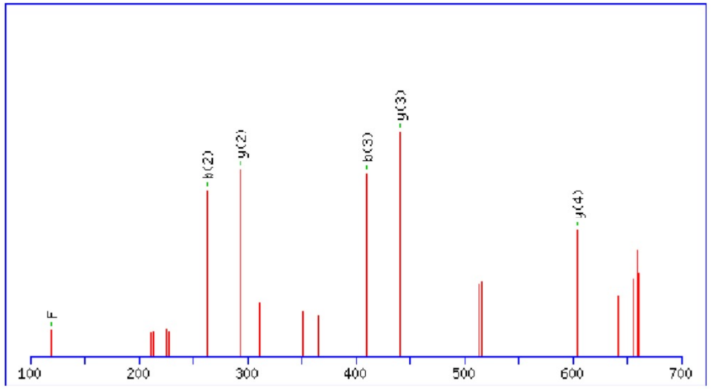

Monoisotopic mass of neutral peptide Mr(calc): 702.37
Fixed modifications: Carbamidomethyl (C)
Ions Score: 21 **Expect:** 4.8
Matches (Bold Red): 7/27 fragment ions using 8 most intense peaks

#	Immon.	a	b	Seq.	y	y*	#
1	72.08	72.08	100.08	V			5
2	136.08	235.14	**263.14**	Y	**604.31**	587.29	4
3	**120.08**	382.21	**410.21**	F	**441.25**	424.22	3
4	**120.08**	529.28	557.28	F	**294.18**	277.15	2
5	101.11			K	147.11	130.09	1

MS/MS Fragmentation of **GSQYWR**
Found in **gi|88853069**, vitronectin precursor [Homo sapiens]

Match to Query 2: 795.352507 from(796.359783,1+)
From data file DATA.TXT

Click mouse within plot area to zoom in by factor of two about that point
Or, Plot from 50 to 800 Da

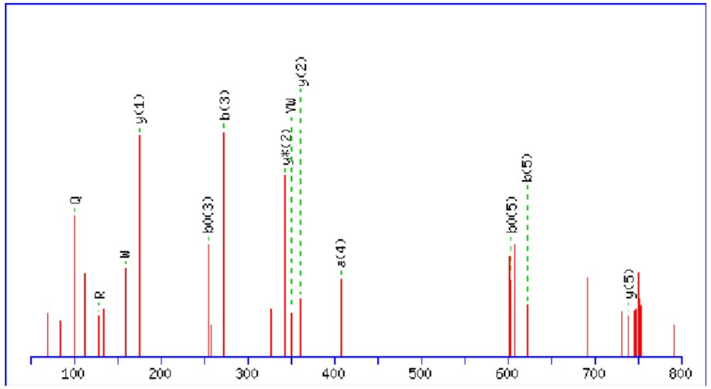

Monoisotopic mass of neutral peptide Mr(calc): 795.37
Fixed modifications: Carbamidomethyl (C)
Ions Score: 11 **Expect:** 19
Matches (Bold Red): 13/58 fragment ions using 29 most intense peaks

#	Immon.	a	a*	a^0	b	b*	b^0	Seq.	v	y	y*	y^0	#
1	30.03	30.03			58.03			G					6
2	60.04	117.07		99.06	145.06		127.05	S	707.33	**739.35**	722.33	721.34	5
3	**101.07**	245.12	228.10	227.11	**273.12**	256.09	**255.11**	Q	579.27	652.32	635.29		4
4	136.08	**408.19**	391.16	390.18	436.18	419.16	418.17	Y	416.20	524.26	507.24		3
5	**159.09**	594.27	577.24	576.26	**622.26**	605.24	**604.25**	W	230.12	**361.20**	**344.17**		2
6	**129.11**							R	74.02	**175.12**	158.09		1

Spot 528 analyzed by MS/MS ion search

Supplementary Figure 4E

MS/MS Fragmentation of **QPQFISR**
Found in **gi|88853069**, vitronectin precursor [Homo sapiens]

Match to Query 10: 874.452578 from(875.459854,1+)
From data file DATA.TXT

Click mouse within plot area to zoom in by factor of two about that point
Or, Plot from 50 to 850 Da

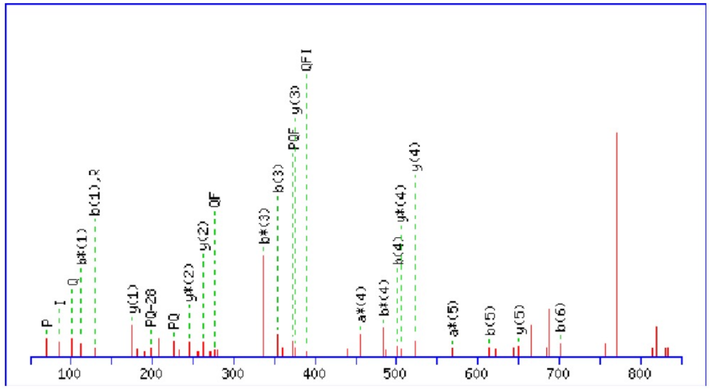

Monoisotopic mass of neutral peptide Mr(calc): 874.47
Fixed modifications: Carbamidomethyl (C)
Ions Score: 38 **Expect:** 0.077
Matches (Bold Red): 30/76 fragment ions using 42 most intense peaks

#	Immon.	a	a*	a^0	b	b*	b^0	Seq.	v	y	y*	y^0	#
1	**101.07**	**101.07**	84.04		**129.07**	**112.04**		Q					7
2	**70.07**	**198.12**	181.10		**226.12**	209.09		P	705.37	747.41	730.39	729.40	6
3	**101.07**	326.18	309.16		**354.18**	**337.15**		Q	577.31	**650.36**	633.34	632.35	5
4	120.08	473.25	**456.22**		**501.25**	**484.22**		F	430.24	**522.30**	**505.28**	504.29	4
5	**86.10**	586.33	**569.31**		**614.33**	597.30		I	317.16	**375.24**	358.21	357.22	3
6	60.04	673.37	656.34	655.36	**701.36**	684.34	683.35	S	230.12	**262.15**	**245.12**	244.14	2
7	**129.11**							R	74.02	**175.12**	158.09		1

MS/MS Fragmentation of **AVRPGYPK**
Found in **gi|88853069**, vitronectin precursor [Homo sapiens]

Match to Query 11: 886.491289 from(887.498565,1+)
From data file DATA.TXT

Click mouse within plot area to zoom in by factor of two about that point
Or, Plot from 50 to 850 Da

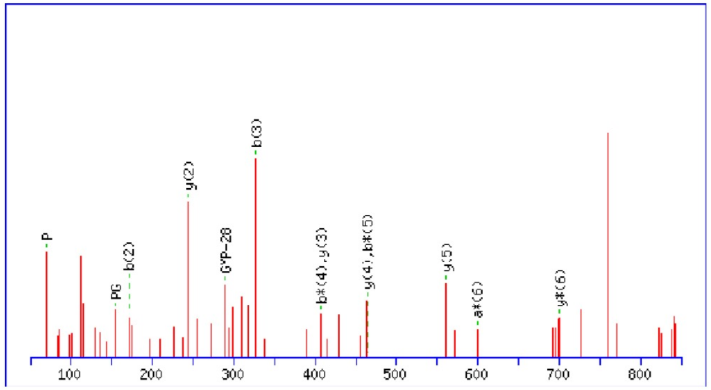

Monoisotopic mass of neutral peptide Mr(calc): 886.50
Fixed modifications: Carbamidomethyl (C)
Ions Score: 22 **Expect:** 3.2
Matches (Bold Red): 15/81 fragment ions using 21 most intense peaks

#	Immon.	a	a*	b	b*	d	Seq.	v	y	y*	#
1	44.05	44.05		72.04			A				8
2	72.08	143.12		**171.11**			V	772.41	816.47	799.45	7
3	129.11	299.22	282.19	**327.21**	310.19	214.15	R	616.31	717.40	**700.38**	6
4	**70.07**	396.27	379.25	424.27	**407.24**	370.26	P		**561.30**	544.28	5
5	30.03	453.29	436.27	481.29	**464.26**		G		**464.25**	447.22	4
6	136.08	616.36	**599.33**	644.35	627.32		Y		**407.23**	390.20	3
7	**70.07**	713.41	696.38	741.40	724.38	687.39	P		**244.17**	227.14	2
8	101.11						K		147.11	130.09	1

Spot 528 analyzed by MS/MS ion search

Supplementary Figure 4E

MS/MS Fragmentation of **FEDGVLDPDYPR**
Found in **gi|88853069**, vitronectin precursor [Homo sapiens]

Match to Query 30: 1421.635729 from(1422.643005,1+)
From data file DATA.TXT

Click mouse within plot area to zoom in by factor of two about that point
Or, Plot from 50 to 1050 Da

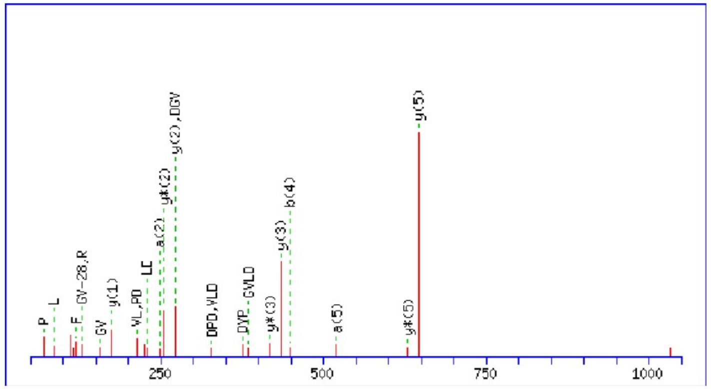

Monoisotopic mass of neutral peptide Mr(calc): 1421.65
Fixed modifications: Carbamidomethyl (C)
Ions Score: 33 **Expect:** 0.094
Matches (Bold Red): 29/164 fragment ions using 23 most intense peaks

#	Immon.	a	a^0	b	b^0	Seq.	v	y	y*	y^0	#
1	**120.08**	**120.08**		148.08		F					12
2	102.05	**249.12**	231.11	277.12	259.11	E	1201.55	1275.59	1258.56	1257.57	11
3	88.04	364.15	346.14	392.15	374.13	D	1086.52	1146.54	1129.52	1128.53	10
4	30.03	421.17	403.16	**449.17**	431.16	G		1031.52	1014.49	1013.51	9
5	72.08	**520.24**	502.23	548.24	530.22	V	930.43	974.49	957.47	956.48	8
6	**86.10**	633.32	615.31	661.32	643.31	L	817.35	875.43	858.40	857.42	7
7	88.04	748.35	730.34	776.35	758.34	D	702.32	762.34	745.32	744.33	6
8	**70.07**	845.40	827.39	873.40	855.39	P	605.27	**647.31**	**630.29**	629.30	5
9	88.04	960.43	942.42	988.43	970.42	D	490.24	550.26	533.24	532.25	4
10	136.08	1123.49	1105.48	1151.49	1133.48	Y	327.18	**435.24**	**418.21**		3
11	**70.07**	1220.55	1202.54	1248.54	1230.53	P	230.12	**272.17**	**255.15**		2
12	**129.11**					R	74.02	**175.12**	158.09		1

MS/MS Fragmentation of **IYISGMAPRPSLAK**
Found in **gi|88853069**, vitronectin precursor [Homo sapiens]

Match to Query 34: 1518.808592 from(1519.815868,1+)
From data file DATA.TXT

Click mouse within plot area to zoom in by factor of two about that point
Or, Plot from 0 to 1500 Da

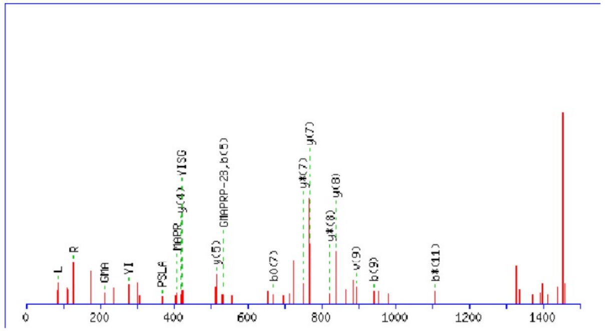

Monoisotopic mass of neutral peptide Mr(calc): 1518.82
Fixed modifications: Carbamidomethyl (C)
Variable modifications:
M6 : Oxidation (M)
Ions Score: 17 **Expect:** 4
Matches (Bold Red): 23/218 fragment ions using 42 most intense peaks

#	Immon.	a	a*	a^0	b	b*	b^0	d	Seq.	v	y	y*	y^0	#
1	**86.10**	**86.10**			114.09				I					14
2	136.08	249.16			**277.15**				Y	1234.69	1342.75	1325.72	1324.74	13
3	**86.10**	362.24			390.24				I	1121.61	1179.68	1162.66	1161.67	12
4	60.04	449.28		431.27	477.27		459.26		S	1034.57	1066.60	1049.57	1048.59	11
5	30.03	506.30		488.29	**534.29**		516.28		G		979.57	962.54	961.56	10
6	56.05	589.33		571.32	617.33		599.32		M	**894.52**	922.55	905.52	904.54	9
7	44.05	660.37		642.36	688.37		**670.36**		A	823.48	**839.51**	**822.48**	821.50	8
8	70.07	757.42		739.41	785.42		767.41		P	726.43	**768.47**	**751.45**	750.46	7
9	**129.11**	913.53	896.50	895.51	**941.52**	924.49	923.51	828.46	R	570.32	671.42	654.39	653.41	6
10	70.07	1010.58	993.55	992.57	1038.57	1021.55	1020.56	984.56	P		**515.32**	498.29	497.31	5
11	60.04	1097.61	1080.58	1079.60	1125.61	**1108.58**	1107.59	1081.62	S		**418.27**	401.24	400.26	4
12	**86.10**	1210.69	1193.67	1192.68	1238.69	1221.66	1220.68	1168.65	L		331.23	314.21		3
13	44.05	1281.73	1264.70	1263.72	1309.73	1292.70	1291.72		A		218.15	201.12		2
14	101.11								K		147.11	130.09		1

Spot 528 analyzed by MS/MS ion search

Supplementary Figure 4E

MS/MS Fragmentation of **DWHGVPGQVDAAMAGR**
Found in **gi|88853069**, vitronectin precursor [Homo sapiens]

Match to Query 36: 1681.730017 from(1682.737293,1+)
From data file DATA.TXT

Click mouse within plot area to zoom in by factor of two about that point
Or, Plot from 100 to 1700 Da

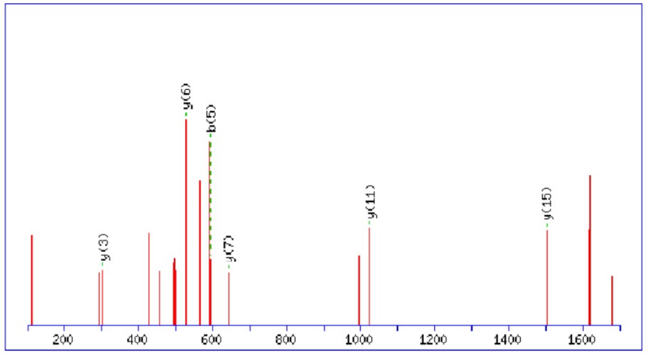

Monoisotopic mass of neutral peptide Mr(calc): 1681.76
Fixed modifications: Carbamidomethyl (C)
Variable modifications:
M13 : Oxidation (M)
Ions Score: 31 **Expect:** 0.077
Matches (Bold Red): 6/273 fragment ions using 13 most intense peaks

#	Immon.	a	a*	a^0	b	b*	b^0	Seq.	v	y	y*	y^0	#
1	88.04	88.04		70.03	116.03		98.02	D					16
2	159.09	274.12		256.11	302.11		284.10	W	1372.67	**1503.75**	1486.72	1485.73	15
3	110.07	411.18		393.17	439.17		421.16	H	1235.61	1317.67	1300.64	1299.66	14
4	30.03	468.20		450.19	496.19		478.18	G		1180.61	1163.58	1162.60	13
5	72.08	567.27		549.26	**595.26**		577.25	V	1079.52	1123.59	1106.56	1105.57	12
6	70.07	664.32		646.31	692.32		674.30	P	982.47	**1024.52**	1007.49	1006.51	11
7	30.03	721.34		703.33	749.34		731.33	G		927.46	910.44	909.45	10
8	101.07	849.40	832.37	831.39	877.40	860.37	859.38	Q	797.39	870.44	853.42	852.43	9
9	72.08	948.47	931.44	930.46	976.46	959.44	958.45	V	698.32	742.38	725.36	724.37	8
10	88.04	1063.50	1046.47	1045.48	1091.49	1074.46	1073.48	D	583.29	**643.32**	626.29	625.31	7
11	44.05	1134.53	1117.51	1116.52	1162.53	1145.50	1144.52	A	512.26	**528.29**	511.26		6
12	44.05	1205.57	1188.54	1187.56	1233.56	1216.54	1215.55	A	441.22	457.25	440.23		5
13	56.05	1288.61	1271.58	1270.60	1316.60	1299.58	1298.59	M	358.18	386.21	369.19		4
14	44.05	1359.64	1342.62	1341.63	1387.64	1370.61	1369.63	A	287.15	**303.18**	286.15		3
15	30.03	1416.67	1399.64	1398.65	1444.66	1427.63	1426.65	G		232.14	215.11		2
16	129.11							R	74.02	175.12	158.09		1

Spot 528 analyzed by MS/MS ion search

Supplementary Figure 4F

Match to: **gi|115298678** Score: **347**
complement C3 preproprotein [Homo sapiens]
Found in search of DATA.TXT

Nominal mass (M_r): **188569**; Calculated pI value: **6.02**
NCBI BLAST search of gi|115298678 against nr
Unformatted sequence string for pasting into other applications

Taxonomy: Homo sapiens

Fixed modifications: Carbamidomethyl (C)
Variable modifications: Oxidation (M)
Cleavage by Trypsin: cuts C-term side of KR unless next residue is P
Sequence Coverage: **6%**

Matched peptides shown in **Bold Red**

```
   1 MGPTSGPSLL LLLLTHLPLA LGSPMYSIIT PNILRLESEE TMVLEAHDAQ
  51 GDVPVTVTVH DFPGKKLVLS SEKTVLTPAT NHMGNVTFTI PANREFKSEK
 101 GRNKFVTVQA TFGTQVVEKV VLVSLQSGYL FIQTDKTIYT PGSTVLYRIF
 151 TVNHKLLPVG RTVMVNIENP EGIPVKQDSL SSQNQLGVLP LSWDIPELVN
 201 MGQWKIRAYY ENSPQQVFST EFEVKEYVLP SFEVIVEPTE KFYYIYNEKG
 251 LEVTITARFL YGKKVEGTAF VIFGIQDGEQ RISLPESLKR IPIEDGSGEV
 301 VLSRKVLLDG VQNPRAEDLV GKSLYVSATV ILHSGSDMVQ AERSGIPIVT
 351 SPYQIHFTKT PKYFKPGMPF DLMVFVTNPD GSPAYRVPVA VQGEDTVQSL
 401 TQGDGVAKLS INTHPSQKPL SITVRTKKQE LSEAEQATRT MQALPYSTVG
 451 NSNNYLHLSV LRTELRPGET LNVNFLLRMD RAHEAKIRYY TYLIMNKGRL
 501 LKAGRQVREP GQDLVVLPLS ITTDFIPSFR LVAYYTLIGA SGQREVVADS
 551 VWVDVKDSCV GSLVVKSGQS EDRQPVPGQQ MTLKIEGDHG ARVVLVAVDK
 601 GVFVLNKKNK LTQSKIWDVV EKADIGCTPG SGKDYAGVFS DAGLTFTSSS
 651 GQQTAQRAEL QCPQPAARRR RSVQLTEKRM DKVGKYPKEL RKCCEDGMRE
 701 NPMRFSCQRR TRFISLGEAC KKVFLDCCNY ITELRRQHAR ASHLGLARSN
 751 LDEDIIAEEN IVSRSEFPES WLWNVEDLKE PPKNGISTKL MNIFLKDSIT
 801 TWEILAVSMS DKKGICVADP FEVTVMQDFF IDLRLPYSVV RNEQVEIRAV
 851 LYNYRQNQEL KVRVELLHNP AFCSLATTKR RHQQTVTIPP KSSLSVPYVI
 901 VPLKTGLQEV EVKAAVYHHF ISDGVRKSLK VVPEGIRMNK TVAVRTLDPE
 951 RLGREGVQKE DIPPADLSDQ VPDTESETRI LLQGTPVAQM TEDAVDAERL
1001 KHLIVTPSGC GEQNMIGMTP TVIAVHYLDE TEQWEKFGLE KRQGALELIK
1051 KGYTQQLAFR QPSSAFAAFV KRAPSTWLTA YVVKVFSLAV NLIAIDSQVL
1101 CGAVKWLILE KQKPDGVFQE DAPVIHQEMI GGLRNNNEKD MALTAFVLIS
1151 LQEAKDICEE QVNSLPGSIT KAGDFLEANY MNLQRSYTVA IAGYALAQMG
1201 RLKGPLLNKF LTTAKDKNRW EDPGKQLYNV EATSYALLAL LQLKDFDFVP
1251 PVVRWLNEQR YYGGGYGSTQ ATFMVFQALA QYQKDAPDHQ ELNLDVSLQL
1301 PSRSSKITHR IHWESASLLR SEETKENEGF TVTAEGKGQG TLSVVTMYHA
1351 KAKDQLTCNK FDLKVTIKPA PETEKRPQDA KNTMILEICT RYRGDQDATM
1401 SILDISMMTG FAPDTDDLKQ LANGVDRYIS KYELDKAFSD RNTLIIYLDK
1451 VSHSEDDCLA FKVHQYFNVE LIQPGAVKVY AYYNLEESCT RFYHPEKEDG
1501 KLNKLCRDEL CRCAEENCFI QKSDDKVTLE ERLDKACEPG VDYVYKTRLV
1551 KVQLSNDFDE YIMAIEQTIK SGSDEVQVGQ QRTFISPIKC REALKLEEKK
1601 HYLMWGLSSD FWGEKPNLSY IIGKDTWVEH WPEEDECQDE ENQKQCQDLG
1651 AFTESMVVFG CPN
```

MS/MS Fragmentation of **TFISPIK**
Found in **gi|115298678**, complement C3 preproprotein [Homo sapiens]

Match to Query 1: 804.489643 from(805.496919,1+)
From data file DATA.TXT

Click mouse within plot area to zoom in by factor of two about that point
Or, Plot from 50 to 800 Da

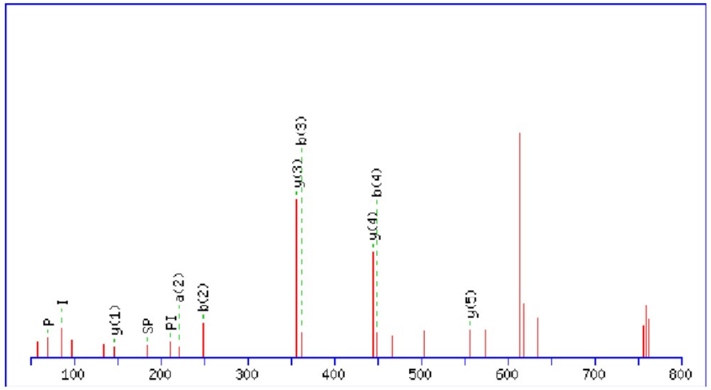

Monoisotopic mass of neutral peptide Mr(calc): 804.47
Fixed modifications: Carbamidomethyl (C)
Ions Score: 20 **Expect:** 3.3
Matches (Bold Red): 13/66 fragment ions using 24 most intense peaks

#	Immon.	a	a^0	b	b^0	Seq.	y	y*	y^0	#
1	74.06	74.06	56.05	102.05	84.04	T				7
2	120.08	**221.13**	203.12	**249.12**	231.11	F	704.43	687.41	686.42	6
3	**86.10**	334.21	316.20	**362.21**	344.20	I	**557.37**	540.34	539.36	5
4	60.04	421.24	403.23	**449.24**	431.23	S	**444.28**	427.26	426.27	4
5	**70.07**	518.30	500.29	546.29	528.28	P	**357.25**	340.22		3
6	**86.10**	631.38	613.37	659.38	641.37	I	260.20	243.17		2
7	101.11					K	**147.11**	130.09		1

Spot 2412 analyzed by MS/MS ion search

Supplementary Figure 4F

MS/MS Fragmentation of **LCRDELCR**
Found in **gi|115298678**, complement C3 preproprotein [Homo sapiens]

Match to Query 6: 1120.521986 from(1121.529262,1+)
From data file DATA.TXT

Click mouse within plot area to zoom in by factor of two about that point
Or, Plot from 0 to 1100 Da

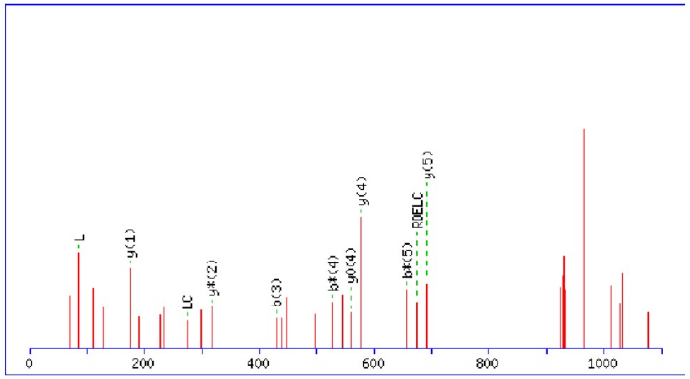

Monoisotopic mass of neutral peptide Mr(calc): 1120.51
Fixed modifications: Carbamidomethyl (C)
Ions Score: 12 **Expect:** 20
Matches (Bold Red): 17/98 fragment ions using 26 most intense peaks

#	Immon.	a	a*	a^0	b	b*	b^0	d	Seq.	v	y	y*	y^0	#
1	**86.10**	**86.10**			114.09				L					8
2	133.04	246.13			**274.12**				C	903.41	1008.43	991.41	990.42	7
3	129.11	402.23	385.20		**430.22**	413.20		317.16	R	747.31	848.40	831.38	830.39	6
4	88.04	517.26	500.23	499.24	545.25	**528.22**	527.24	473.27	D	632.28	**692.30**	675.28	**674.29**	5
5	102.05	646.30	629.27	628.29	**674.29**	**657.27**	656.28	588.29	E	503.24	**577.28**	560.25	**559.27**	4
6	**86.10**	759.38	742.36	741.37	787.38	770.35	769.37	717.33	L	390.16	448.23	431.21		3
7	133.04	919.41	902.39	901.40	947.41	930.38	929.40	830.42	C	230.12	335.15	**318.12**		2
8	129.11								R	74.02	**175.12**	158.09		1

MS/MS Fragmentation of **SDDKVTLEER**
Found in **gi|115298678**, complement C3 preproprotein [Homo sapiens]

Match to Query 9: 1190.596350 from(1191.603626,1+)
From data file DATA.TXT

Click mouse within plot area to zoom in by factor of two about that point
Or, Plot from 0 to 1200 Da

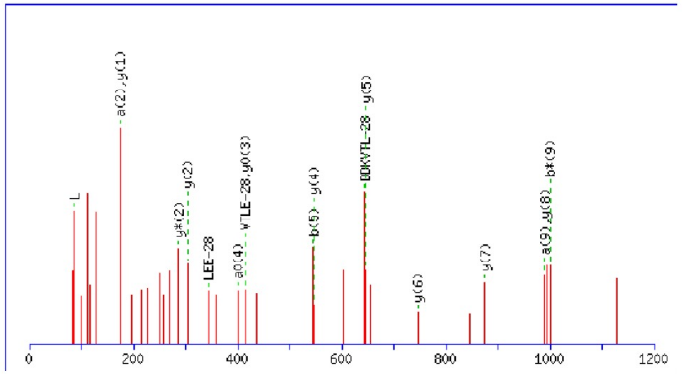

Monoisotopic mass of neutral peptide Mr(calc): 1190.58
Fixed modifications: Carbamidomethyl (C)
Ions Score: 48 **Expect:** 0.0056
Matches (Bold Red): 19/142 fragment ions using 25 most intense peaks

#	Immon.	a	a*	a^0	b	b*	b^0	Seq.	v	y	y*	y^0	#
1	60.04	60.04		42.03	88.04		70.03	S					10
2	88.04	**175.07**		157.06	203.07		185.06	D	1044.53	1104.55	1087.53	1086.54	9
3	88.04	290.10		272.09	318.09		300.08	D	929.51	**989.53**	972.50	971.52	8
4	101.11	418.19	401.17	**400.18**	446.19	429.16	428.18	K	801.41	**874.50**	857.47	856.49	7
5	72.08	517.26	500.24	499.25	**545.26**	528.23	527.25	V	702.34	**746.40**	729.38	728.39	6
6	74.06	618.31	601.28	600.30	646.30	629.28	628.29	T	601.29	**647.34**	630.31	629.33	5
7	**86.10**	731.39	714.37	713.38	759.39	742.36	741.38	L	488.21	**546.29**	529.26	528.28	4
8	102.05	860.44	843.41	842.43	888.43	871.40	870.42	E	359.17	433.20	416.18	**415.19**	3
9	102.05	**989.48**	972.45	971.47	1017.47	**1000.45**	999.46	E	230.12	**304.16**	**287.13**	286.15	2
10	129.11							R	74.02	**175.12**	158.09		1

Spot 2412 analyzed by MS/MS ion search

Supplementary Figure 4F

MS/MS Fragmentation of **IHWESASLLR**
Found in **gi|115298678**, complement C3 preproprotein [Homo sapiens]

Match to Query 10: 1210.664500 from(1211.671776,1+)
From data file DATA.TXT

Click mouse within plot area to zoom in by factor of two about that point
Or, Plot from 50 to 1050 Da

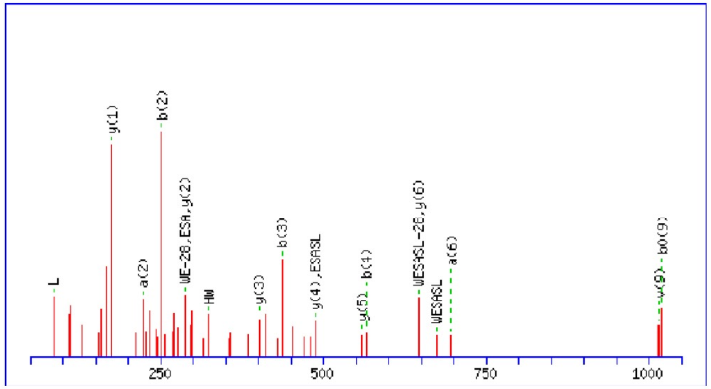

Monoisotopic mass of neutral peptide Mr(calc): 1210.65
Fixed modifications: Carbamidomethyl (C)
Ions Score: 46 **Expect:** 0.0055
Matches (Bold Red): 23/123 fragment ions using 21 most intense peaks

#	Immon.	a	a^0	b	b^0	Seq.	v	y	y*	y^0	#
1	**86.10**	**86.10**		114.09		I					10
2	110.07	**223.16**		**251.15**		H	**1016.52**	1098.57	1081.54	1080.56	9
3	159.09	409.23		**437.23**		W	830.44	961.51	944.48	943.50	8
4	102.05	538.28	520.27	**566.27**	548.26	E	701.39	775.43	758.40	757.42	7
5	60.04	625.31	607.30	653.30	635.29	S	614.36	**646.39**	629.36	628.38	6
6	44.05	**696.35**	678.34	724.34	706.33	A	543.32	**559.36**	542.33	541.35	5
7	60.04	783.38	765.37	811.37	793.36	S	456.29	**488.32**	471.29	470.31	4
8	**86.10**	896.46	878.45	924.46	906.45	L	343.21	**401.29**	384.26		3
9	**86.10**	1009.55	991.54	1037.54	**1019.53**	L	230.12	**288.20**	271.18		2
10	129.11					R	74.02	**175.12**	158.09		1

MS/MS Fragmentation of **NTMILEICTR**
Found in **gi|115298678**, complement C3 preproprotein [Homo sapiens]

Match to Query 15: 1265.623234 from(1266.630510,1+)
From data file DATA.TXT

Click mouse within plot area to zoom in by factor of two about that point
Or, Plot from 0 to 1300 Da

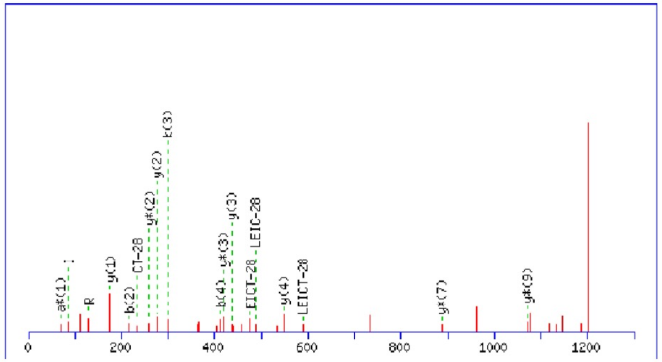

Monoisotopic mass of neutral peptide Mr(calc): 1265.61
Fixed modifications: Carbamidomethyl (C)
Variable modifications:
M3 : Oxidation (M)
Ions Score: 28 **Expect:** 0.43
Matches (Bold Red): 20/144 fragment ions using 30 most intense peaks

#	Immon.	a	a*	a^0	b	b*	b^0	Seq.	v	y	y*	y^0	#
1	87.06	87.06	**70.03**		115.05	98.02		N					10
2	74.06	188.10	171.08	170.09	**216.10**	199.07	198.09	T	1042.53	1088.58	**1071.55**	1070.57	9
3	56.05	271.14	254.11	253.13	**299.13**	282.11	281.12	M	959.50	987.53	970.50	969.52	8
4	**86.10**	384.22	367.20	366.21	**412.22**	395.19	394.21	I	846.41	904.49	**887.47**	886.48	7
5	**86.10**	497.31	480.28	479.30	525.30	508.28	507.29	L	733.33	791.41	774.38	773.40	6
6	102.05	626.35	609.32	608.34	654.35	637.32	636.34	E	604.29	678.32	661.30	660.31	5
7	**86.10**	739.43	722.41	721.42	767.43	750.40	749.42	I	491.20	**549.28**	532.25	531.27	4
8	133.04	899.47	882.44	881.45	927.46	910.43	909.45	C	331.17	**436.20**	**419.17**	418.19	3
9	74.06	1000.51	983.49	982.50	1028.51	1011.48	1010.50	T	230.12	**276.17**	**259.14**	258.16	2
10	**129.11**							R	74.02	**175.12**	158.09		1

Spot 2412 analyzed by MS/MS ion search

Supplementary Figure 4F

MS/MS Fragmentation of **SGSDEVQVGQQR**
Found in **gi|115298678**, complement C3 preproprotein [Homo sapiens]

Match to Query 17: 1288.625563 from(1289.632839,1+)
From data file DATA.TXT

Click mouse within plot area to zoom in by factor of two about that point
Or, Plot from 0 to 1300 Da

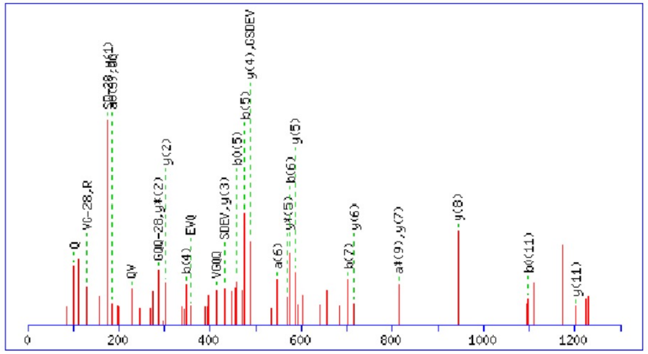

Monoisotopic mass of neutral peptide Mr(calc): 1288.60
Fixed modifications: Carbamidomethyl (C)
Ions Score: 63 **Expect:** 0.00014
Matches (Bold Red): 35/173 fragment ions using 38 most intense peaks

#	Immon.	a	a*	a^0	b	b*	b^0	Seq.	v	y	y*	y^0	#
1	60.04	60.04		42.03	88.04		70.03	S					12
2	30.03	117.07		99.06	145.06		127.05	G		**1202.58**	1185.55	1184.57	11
3	60.04	204.10		**186.09**	232.09		214.08	S	1113.53	1145.55	1128.53	1127.54	10
4	88.04	319.12		301.11	**347.12**		329.11	D	998.50	1058.52	1041.50	1040.51	9
5	102.05	448.17		430.16	**476.16**		**458.15**	E	869.46	**943.50**	926.47	925.48	8
6	72.08	**547.24**		529.23	**575.23**		557.22	V	770.39	**814.45**	797.43		7
7	**101.07**	675.29	658.27	657.28	**703.29**	686.26	685.28	Q	642.33	**715.38**	698.36		6
8	72.08	774.36	757.34	756.35	802.36	785.33	784.35	V	543.26	**587.33**	**570.30**		5
9	30.03	831.38	**814.36**	813.37	859.38	842.35	841.37	G		**488.26**	471.23		4
10	**101.07**	959.44	942.42	941.43	987.44	970.41	969.43	Q	358.18	**431.24**	414.21		3
11	**101.07**	1087.50	1070.47	1069.49	1115.50	1098.47	**1097.49**	Q	230.12	**303.18**	**286.15**		2
12	**129.11**							R	74.02	**175.12**	158.09		1

MS/MS Fragmentation of **CAEENCFIQK**
Found in **gi|115298678**, complement C3 preproprotein [Homo sapiens]

Match to Query 18: 1297.550670 from(1298.557946,1+)
From data file DATA.TXT

Click mouse within plot area to zoom in by factor of two about that point
Or, Plot from 0 to 1300 Da

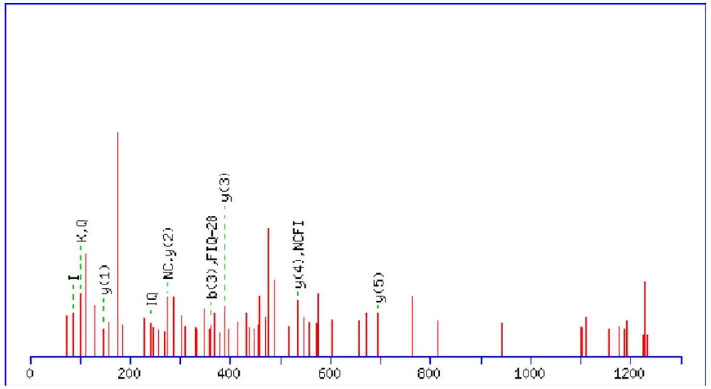

Monoisotopic mass of neutral peptide Mr(calc): 1297.54
Fixed modifications: Carbamidomethyl (C)
Ions Score: 4 **Expect:** 59
Matches (Bold Red): 13/117 fragment ions using 53 most intense peaks

#	Immon.	a	a*	a^0	b	b*	b^0	Seq.	y	y*	y^0	#
1	133.04	133.04			161.04			C				10
2	44.05	204.08			232.08			A	1138.52	1121.49	1120.51	9
3	102.05	333.12		315.11	**361.12**		343.11	E	1067.48	1050.46	1049.47	8
4	102.05	462.17		444.15	490.16		472.15	E	938.44	921.41	920.43	7
5	87.06	576.21	559.18	558.20	604.20	587.18	586.19	N	809.40	792.37		6
6	133.04	736.24	719.21	718.23	764.23	747.21	746.22	C	**695.35**	678.33		5
7	120.08	883.31	866.28	865.30	911.30	894.28	893.29	F	**535.32**	518.30		4
8	**86.10**	996.39	979.36	978.38	1024.39	1007.36	1006.38	I	**388.26**	371.23		3
9	**101.07**	1124.45	1107.42	1106.44	1152.44	1135.42	1134.43	Q	**275.17**	258.14		2
10	**101.11**							K	**147.11**	130.09		1

Spot 2412 analyzed by MS/MS ion search

Supplementary Figure 4F

MS/MS Fragmentation of **GQGTLSVVTMYHAK**
Found in **gi|115298678**, complement C3 preproprotein [Homo sapiens]

Match to Query 19: 1506.772564 from(1507.779840,1+)
From data file DATA.TXT

Click mouse within plot area to zoom in by factor of two about that point
Or, Plot from 100 to 1500 Da

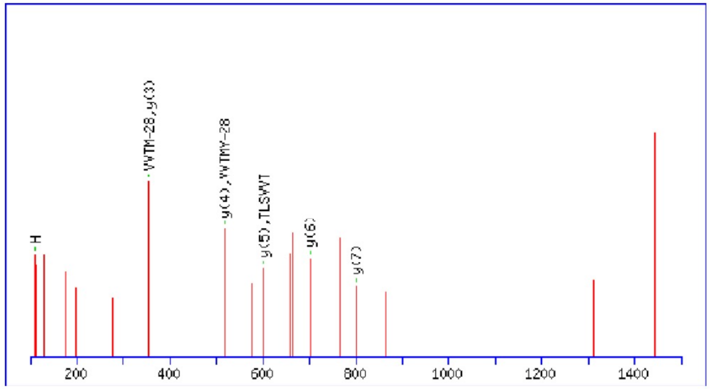

Monoisotopic mass of neutral peptide Mr(calc): 1506.75
Fixed modifications: Carbamidomethyl (C)
Variable modifications:
M10 : Oxidation (M)
Ions Score: 25 **Expect:** 0.72
Matches (**Bold Red**): 9/214 fragment ions using 18 most intense peaks

#	Immon.	a	a*	a^0	b	b*	b^0	Seq.	y	y*	y^0	#
1	30.03	30.03			58.03			G				14
2	101.07	158.09	141.07		186.09	169.06		Q	1386.74	1369.71	1368.73	13
3	30.03	215.11	198.09		243.11	226.08		G	1258.68	1241.65	1240.67	12
4	74.06	316.16	299.14	298.15	344.16	327.13	326.15	T	1201.66	1184.63	1183.65	11
5	86.10	429.25	412.22	411.24	457.24	440.21	439.23	L	1100.61	1083.58	1082.60	10
6	60.04	516.28	499.25	498.27	544.27	527.25	526.26	S	987.53	970.50	969.52	9
7	72.08	615.35	598.32	597.34	643.34	626.31	625.33	V	900.49	883.47	882.48	8
8	72.08	714.41	697.39	696.40	742.41	725.38	724.40	V	**801.43**	784.40	783.41	7
9	74.06	815.46	798.44	797.45	843.46	826.43	825.45	T	**702.36**	685.33	684.35	6
10	56.05	898.50	881.47	880.49	926.49	909.47	908.48	M	**601.31**	584.28		5
11	136.08	1061.56	1044.54	1043.55	1089.56	1072.53	1071.55	Y	**518.27**	501.25		4
12	**110.07**	1198.62	1181.59	1180.61	1226.62	1209.59	1208.61	H	**355.21**	338.18		3
13	44.05	1269.66	1252.63	1251.65	1297.65	1280.63	1279.64	A	218.15	201.12		2
14	101.11							K	147.11	130.09		1

MS/MS Fragmentation of **VYAYYNLEESCTR**
Found in **gi|115298678**, complement C3 preproprotein [Homo sapiens]

Match to Query 22: 1666.767349 from(1667.774625,1+)
From data file DATA.TXT

Click mouse within plot area to zoom in by factor of two about that point
Or, Plot from 0 to 1600 Da

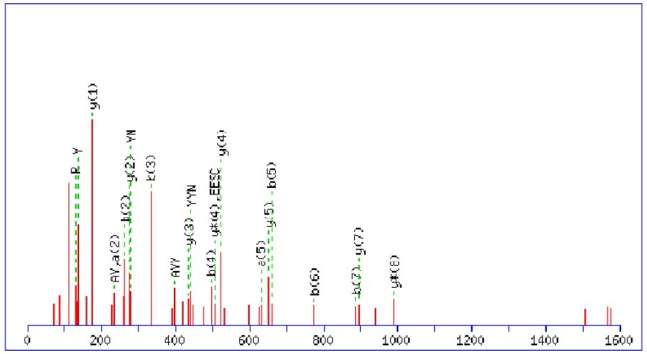

Monoisotopic mass of neutral peptide Mr(calc): 1666.73
Fixed modifications: Carbamidomethyl (C)
Ions Score: 66 **Expect:** 4.7e-005
Matches (**Bold Red**): 27/177 fragment ions using 26 most intense peaks

#	Immon.	a	a*	a^0	b	b*	b^0	Seq.	v	y	y*	y^0	#
1	72.08	72.08			100.08			V					13
2	**136.08**	**235.14**			**263.14**			Y	1460.61	1568.67	1551.64	1550.66	12
3	44.05	306.18			**334.18**			A	1389.57	1405.61	1388.58	1387.59	11
4	**136.08**	469.24			**497.24**			Y	1226.51	1334.57	1317.54	1316.56	10
5	**136.08**	**632.31**			**660.30**			Y	1063.45	1171.50	1154.48	1153.49	9
6	87.06	746.35	729.32		**774.35**	757.32		N	949.40	1008.44	**991.41**	990.43	8
7	86.10	859.43	842.41		**887.43**	870.40		L	836.32	**894.40**	877.37	876.39	7
8	102.05	988.48	971.45	970.47	1016.47	999.45	998.46	E	707.28	781.31	764.29	763.30	6
9	102.05	1117.52	1100.49	1099.51	1145.51	1128.49	1127.50	E	578.24	**652.27**	635.25	634.26	5
10	60.04	1204.55	1187.53	1186.54	1232.55	1215.52	1214.54	S	491.20	**523.23**	**506.20**	505.22	4
11	133.04	1364.58	1347.56	1346.57	1392.58	1375.55	1374.57	C	331.17	**436.20**	419.17	418.19	3
12	74.06	1465.63	1448.60	1447.62	1493.63	1476.60	1475.61	T	230.12	**276.17**	259.14	258.16	2
13	**129.11**							R	74.02	**175.12**	158.09		1

Spot 2412 analyzed by MS/MS ion search

Supplementary Figure 4F

MS/MS Fragmentation of **VTIKPAPETEKRPQDAK**
Found in **gi|115298678**, complement C3 preproprotein [Homo sapiens]

Match to Query 24: 1907.070280 from(1908.077556,1+)
From data file DATA.TXT

Click mouse within plot area to zoom in by factor of two about that point
Or, Plot from 0 to 1800 Da

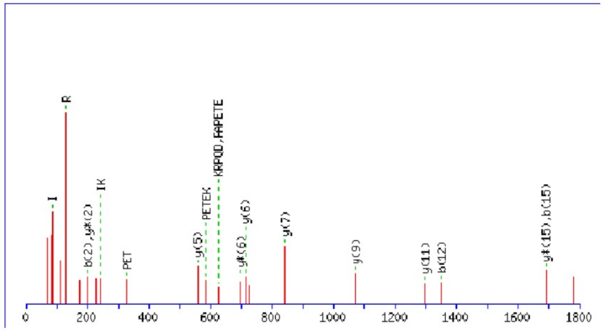

Monoisotopic mass of neutral peptide Mr(calc): 1907.05
Fixed modifications: Carbamidomethyl (C)
Ions Score: 41 **Expect:** 0.0069
Matches (Bold Red): 18/278 fragment ions using 16 most intense peaks

#	Immon.	a	a*	a^0	b	b*	b^0	d	Seq.	v	y	y*	y^0	#
1	72.08	72.08			100.08				V					17
2	74.06	173.13		155.12	**201.12**		183.11		T	1762.94	1808.99	1791.96	1790.98	16
3	**86.10**	286.21		268.20	314.21		296.20		I	1649.86	1707.94	**1690.91**	1689.93	15
4	101.11	414.31	397.28	396.30	442.30	425.28	424.29		K	1521.77	1594.85	1577.83	1576.84	14
5	70.07	511.36	494.33	493.35	539.36	522.33	521.34		P	1424.71	1466.76	1449.73	1448.75	13
6	44.05	582.40	565.37	564.39	610.39	593.37	592.38		A	1353.68	1369.71	1352.68	1351.70	12
7	70.07	679.45	662.42	661.44	707.45	690.42	689.43		P	1256.62	**1298.67**	1281.64	1280.66	11
8	102.05	808.49	791.47	790.48	836.49	819.46	818.48		E	1127.58	1201.62	1184.59	1183.61	10
9	74.06	909.54	892.51	891.53	937.54	920.51	919.52		T	1026.53	**1072.57**	1055.55	1054.56	9
10	102.05	1038.58	1021.56	1020.57	1066.58	1049.55	1048.57		E	897.49	971.53	954.50	953.52	8
11	101.11	1166.68	1149.65	1148.67	1194.67	1177.65	1176.66		K	769.40	**842.48**	825.46	824.47	7
12	**129.11**	1322.78	1305.75	1304.77	**1350.77**	1333.75	1332.76	1237.72	R	613.29	**714.39**	**697.36**	696.38	6
13	70.07	1419.83	1402.81	1401.82	1447.83	1430.80	1429.82	1393.82	P		**558.29**	541.26	540.28	5
14	101.07	1547.89	1530.86	1529.88	1575.89	1558.86	1557.87	1490.87	Q		461.24	444.21	443.22	4
15	88.04	1662.92	1645.89	1644.91	**1690.91**	1673.89	1672.90	1618.93	D		333.18	316.15	315.17	3
16	44.05	1733.95	1716.93	1715.94	1761.95	1744.92	1743.94		A		218.15	**201.12**		2
17	101.11								K		147.11	130.09		1

Spot 2412 analyzed by MS/MS ion search

Supplementary Figure 4G

Match to: **gi|66932947** Score: **218**
alpha-2-macroglobulin isoform a precursor [Homo sapiens]
Found in search of DATA.TXT

Nominal mass (M_r): **164614**; Calculated pI value: **6.00**
NCBI BLAST search of gi|66932947 against nr
Unformatted sequence string for pasting into other applications

Taxonomy: Homo sapiens

Fixed modifications: Carbamidomethyl (C)
Variable modifications: Oxidation (M)
Cleavage by Trypsin: cuts C-term side of KR unless next residue is P
Sequence Coverage: **3%**

Matched peptides shown in **Bold Red**

1 MGKNKLLHPS LVLLLLVLLP TDASVSGKPQ YMVLVPSLLH TETTEKGCVL
51 LSYLNETVTV SASLESVRGN RSLFTDLEAE NDVLHCVAFA VPKSSSNEEV
101 MFLTVQVKGP TQEFKKRTTV MVKNEDSLVF VQTDKSIYKP GQTVKFRVVS
151 MDENFHPLNE LIPLVYIQDP KGNRIAQWQS FQLEGGLKQF SFPLSSEPFQ
201 GSYKVVVQKK SGGRTEHPFT VEEFVLPKFE VQVTVPKIIT ILEEEMNVSV
251 CGLYTYGKPV PGHVTVSICR KYSDASDCHG EDSQAFCEKF SGQLNSHGCF
301 YQQVKTKVFQ LKRKEYEMKL HTEAQIQEEG TVVELTGRQS SEITRTITKL
351 SFVKVDSHFR QGIPFFGQVR LVDGKGVPIP NKVIFIRGNE ANYYSNATTD
401 EHGLVQFSIN TTNVMGTSLT VRVNYKDRSP CYGYQWVSEE HEEAHHTAYL
451 VFSPSKSFVH LEPMSHELPC GHTQTVQAHY ILNGGTLLGL KKLSFYYLIM
501 AKGGIVRTGT HGLLVKQEDM KGHFSISIPV KSDIAPVARL LIYAVLPTGD
551 VIGDSAKYDV ENCLANKVDL SFSPSQSLPA SHAHLRVTAA PQSVCALRAV
601 DQSVLLMKPD AELSASSVYN LLPEKDLTGF PGPLNDQDDE DCINRHNVYI
651 NGITYTPVSS TNEKDMYSFL EDMGLKAFTN SKIRKPKMCP QLQQYEMHGP
701 EGLRVGFYES DVMGRGHARL VHVEEPHTET VRKYFPETWI WDLVVVNSAG
751 VAEVGVTVPD TITEWKAGAF CLSEDAGLGI SSTASLRAFQ PFFVELTMPY
801 SVIRGEAFTL KATVLNYLPK CIRVSVQLEA SPAFLAVPVE KEQAPHCICA
851 NGRQTVSWAV TPKSLGNVNF TVSAEALESQ ELCGTEVPSV PEHGRKDTVI
901 KPLLVEPEGL EKETTFNSLL CPSGGEVSEE LSLKLPPNVV EESARASVSV
951 LGDILGSAMQ NTQNLLQMPY GCGEQNMVLF APNIYVLDYL NETQQLTPEI
1001 K**SKAIGYLNT GYQR**QLNYK**H YDGSYSTFGE R**YGRNQGNTW LTAFVLK**TFA**
1051 **QAR**AYIFIDE AHITQALIWL SQRQKDNGCF RSSGSLLNNA IKGGVEDEVT
1101 LSAYITIALL EIPLTVTHPV VRNALFCLES AWKTAQEGDH GSHVYTKALL
1151 AYAFALAGNQ DKRKEVLKSL NEEAVKKDNS VHWERPQKPK APVGHFYEPQ
1201 APSAEVEMTS YVLLAYLTAQ PAPTSEDLTS ATNIVKWITK QQNAQGGFSS
1251 TQDTVVALHA LSK**YGAATFT R**TGKAAQVTI QSSGTFSSK**F QVDNNNR**LLL
1301 QQVSLPELPG EYSMKVTGEG CVYLQTSLKY NILPEKEEFP FALGVQTLPQ
1351 TCDEPKAHTS FQISLSVSYT GSRSASNMAI VDVKMVSGFI PLKPTVKMLE
1401 RSNHVSRTEV SSNHVLIYLD KVSNQTLSLF FTVLQDVPVR DLKPAIVKVY
1451 DYYETDEFAI AEYNAPCSKD LGNA

MS/MS Fragmentation of **TFAQAR**
Found in **gi|66932947**, alpha-2-macroglobulin isoform a precursor [Homo sapiens]

Match to Query 1: 692.374711 from(693.381987,1+)
From data file DATA.TXT

Click mouse within plot area to zoom in by factor of two about that point
Or, Plot from 50 to 700 Da

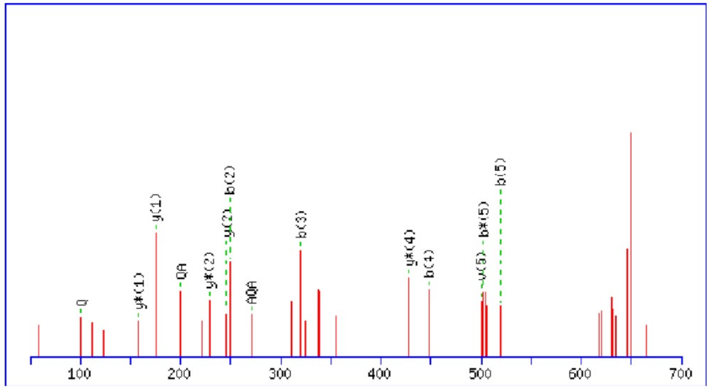

Monoisotopic mass of neutral peptide Mr(calc): 692.36
Fixed modifications: Carbamidomethyl (C)
Ions Score: 20 **Expect:** 4.2
Matches (Bold Red): 15/57 fragment ions using 28 most intense peaks

#	Immon.	a	a*	a^0	b	b*	b^0	Seq.	v	y	y*	#
1	74.06	74.06		56.05	102.05		84.04	T				6
2	120.08	221.13		203.12	**249.12**		231.11	F	**500.26**	592.32	575.29	5
3	44.05	292.17		274.16	**320.16**		302.15	A	429.22	445.25	**428.23**	4
4	**101.07**	420.22	403.20	402.21	**448.22**	431.19	430.21	Q	301.16	374.21	357.19	3
5	44.05	491.26	474.23	473.25	**519.26**	**502.23**	501.25	A	230.12	**246.16**	**229.13**	2
6	129.11							R	74.02	**175.12**	**158.09**	1

Spot 2618 analyzed by MS/MS ion search

Supplementary Figure 4G

MS/MS Fragmentation of **YGAATFTR**
Found in **gi|66932947**, alpha-2-macroglobulin isoform a precursor [Homo sapiens]

Match to Query 6: 885.460118 from(886.467394,1+)
From data file DATA.TXT

Click mouse within plot area to zoom in by factor of two about that point
Or, Plot from 50 to 850 Da

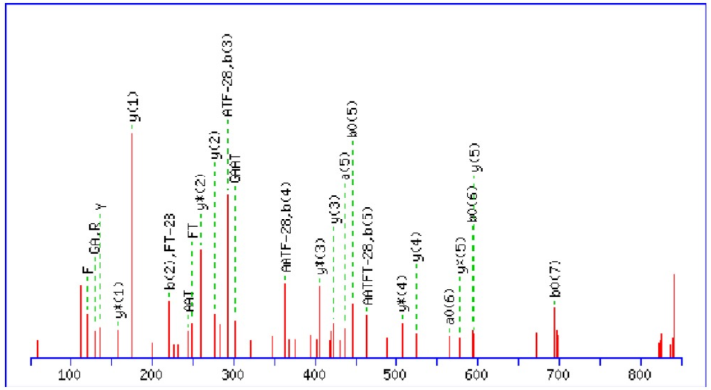

Monoisotopic mass of neutral peptide Mr(calc): 885.43
Fixed modifications: Carbamidomethyl (C)
Ions Score: 36 **Expect:** 0.11
Matches (Bold Red): 33/84 fragment ions using 37 most intense peaks

#	Immon.	a	a^0	b	b^0	Seq.	v	y	y*	y^0	#
1	**136.08**	**136.08**		164.07		Y					8
2	30.03	193.10		**221.09**		G		723.38	706.35	705.37	7
3	44.05	264.13		**292.13**		A	650.33	666.36	649.33	648.35	6
4	44.05	335.17		**363.17**		A	579.29	**595.32**	**578.29**	577.31	5
5	74.06	**436.22**	418.21	**464.21**	**446.20**	T	478.24	**524.28**	**507.26**	506.27	4
6	**120.08**	583.29	**565.28**	611.28	**593.27**	F	331.17	**423.24**	**406.21**	405.22	3
7	74.06	684.34	666.32	712.33	**694.32**	T	230.12	**276.17**	**259.14**	258.16	2
8	**129.11**					R	74.02	**175.12**	**158.09**		1

MS/MS Fragmentation of **FQVDNNNR**
Found in **gi|66932947**, alpha-2-macroglobulin isoform a precursor [Homo sapiens]

Match to Query 10: 1005.486945 from(1006.494221,1+)
From data file DATA.TXT

Click mouse within plot area to zoom in by factor of two about that point
Or, Plot from 100 to 1000 Da

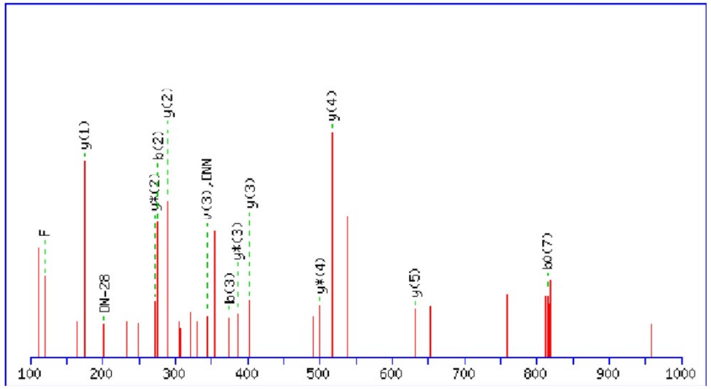

Monoisotopic mass of neutral peptide Mr(calc): 1005.46
Fixed modifications: Carbamidomethyl (C)
Ions Score: 24 **Expect:** 1.5
Matches (Bold Red): 16/96 fragment ions using 29 most intense peaks

#	Immon.	a	a*	a^0	b	b*	b^0	Seq.	v	y	y*	y^0	#
1	**120.08**	**120.08**			148.08			F					8
2	101.07	248.14	231.11		**276.13**	259.11		Q	786.35	859.40	842.38	841.39	7
3	72.08	347.21	330.18		**375.20**	358.18		V	687.28	731.34	714.32	713.33	6
4	88.04	462.23	445.21	444.22	490.23	473.20	472.22	D	572.25	**632.27**	615.25	614.26	5
5	87.06	576.28	559.25	558.27	604.27	587.25	586.26	N	458.21	**517.25**	**500.22**		4
6	87.06	690.32	673.29	672.31	718.32	701.29	700.30	N	**344.17**	**403.20**	**386.18**		3
7	87.06	804.36	787.34	786.35	832.36	815.33	**814.35**	N	230.12	**289.16**	**272.14**		2
8	129.11							R	74.02	**175.12**	158.09		1

Spot 2618 analyzed by MS/MS ion search

Supplementary Figure 4G

MS/MS Fragmentation of **AIGYLNTGYQR**
Found in **gi|66932947**, alpha-2-macroglobulin isoform a precursor [Homo sapiens]

Match to Query 15: 1254.672169 from(1255.679445,1+)
From data file DATA.TXT

Click mouse within plot area to zoom in by factor of two about that point
Or, Plot from 0 to 1200 Da

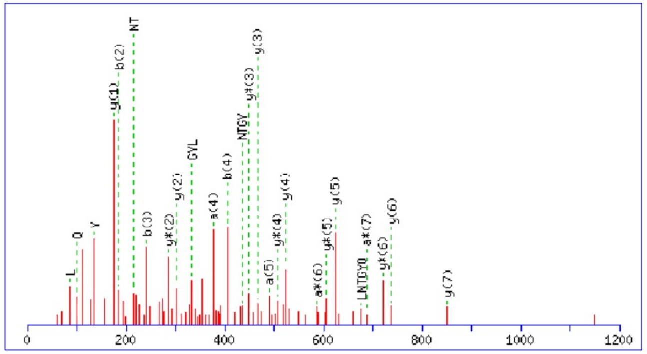

Monoisotopic mass of neutral peptide Mr(calc): 1254.64
Fixed modifications: Carbamidomethyl (C)
Ions Score: 61 **Expect:** 0.00023
Matches (Bold Red): 29/143 fragment ions using 30 most intense peaks

#	Immon.	a	a*	a^0	b	b*	b^0	Seq.	v	y	y*	y^0	#
1	44.05	44.05			72.04			A					11
2	**86.10**	157.13			**185.13**			I	1126.53	1184.61	1167.58	1166.60	10
3	30.03	214.15			**242.15**			G		1071.52	1054.50	1053.51	9
4	**136.08**	**377.22**			**405.21**			Y	906.44	1014.50	997.47	996.49	8
5	**86.10**	**490.30**			518.30			L	793.36	**851.44**	834.41	833.43	7
6	87.06	604.35	**587.32**		632.34	615.31		N	679.32	**738.35**	**721.33**	720.34	6
7	74.06	705.39	**688.37**	687.38	733.39	716.36	715.38	T	578.27	**624.31**	**607.28**	606.30	5
8	30.03	762.41	745.39	744.40	790.41	773.38	772.40	G		**523.26**	**506.24**		4
9	**136.08**	925.48	908.45	907.47	953.47	936.45	935.46	Y	358.18	**466.24**	**449.21**		3
10	**101.07**	1053.54	1036.51	1035.53	1081.53	1064.50	1063.52	Q	230.12	**303.18**	**286.15**		2
11	129.11							R	74.02	**175.12**	158.09		1

MS/MS Fragmentation of **HYDGSYSTFGER**
Found in **gi|66932947**, alpha-2-macroglobulin isoform a precursor [Homo sapiens]

Match to Query 20: 1417.633436 from(1418.640712,1+)
From data file DATA.TXT

Click mouse within plot area to zoom in by factor of two about that point
Or, Plot from 100 to 1300 Da

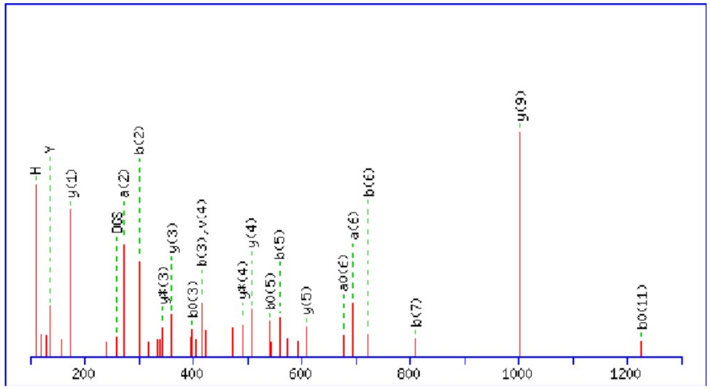

Monoisotopic mass of neutral peptide Mr(calc): 1417.59
Fixed modifications: Carbamidomethyl (C)
Ions Score: 67 **Expect:** 4.3e-005
Matches (Bold Red): 24/164 fragment ions using 21 most intense peaks

#	Immon.	a	a^0	b	b^0	Seq.	v	y	y*	y^0	#
1	**110.07**	**110.07**		138.07		H					12
2	**136.08**	**273.13**		**301.13**		Y	1173.48	1281.54	1264.51	1263.53	11
3	88.04	388.16	370.15	**416.16**	**398.15**	D	1058.45	1118.47	1101.45	1100.46	10
4	30.03	445.18	427.17	473.18	455.17	G		**1003.45**	986.42	985.44	9
5	60.04	532.22	514.20	**560.21**	**542.20**	S	914.40	946.43	929.40	928.42	8
6	**136.08**	**695.28**	**677.27**	**723.27**	705.26	Y	751.34	859.39	842.37	841.38	7
7	60.04	782.31	764.30	**810.31**	792.29	S	664.30	696.33	679.30	678.32	6
8	74.06	883.36	865.35	911.35	893.34	T	563.26	**609.30**	592.27	591.29	5
9	120.08	1030.43	1012.42	1058.42	1040.41	F	**416.19**	**508.25**	**491.22**	490.24	4
10	30.03	1087.45	1069.44	1115.44	1097.43	G		**361.18**	**344.16**	343.17	3
11	102.05	1216.49	1198.48	1244.49	**1226.47**	E	230.12	304.16	287.13	286.15	2
12	129.11					R	74.02	**175.12**	158.09		1

Spot 2618 analyzed by MS/MS ion search

Supplementary Figure 4G

MS/MS Fragmentation of **SKAIGYLNTGYQR**
Found in **gi|66932947**, alpha-2-macroglobulin isoform a precursor [Homo sapiens]

Match to Query 24: 1469.810753 from(1470.818029,1+)
From data file DATA.TXT

Click mouse within plot area to zoom in by factor of two about that point
Or, Plot from 0 to 1500 Da

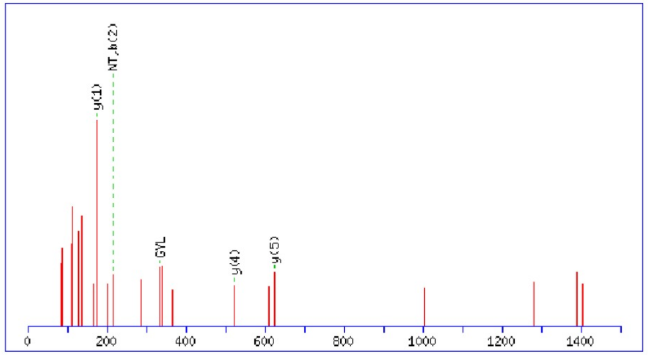

Monoisotopic mass of neutral peptide Mr(calc): 1469.76
Fixed modifications: Carbamidomethyl (C)
Ions Score: 11 **Expect:** 18
Matches (Bold Red): 7/205 fragment ions using 9 most intense peaks

#	Immon.	a	a*	a^0	b	b*	b^0	Seq.	v	y	y*	y^0	#
1	60.04	60.04		42.03	88.04		70.03	S					13
2	101.11	188.14	171.11	170.13	**216.13**	199.11	198.12	K	1310.65	1383.74	1366.71	1365.73	12
3	44.05	259.18	242.15	241.17	287.17	270.14	269.16	A	1239.61	1255.64	1238.62	1237.63	11
4	86.10	372.26	355.23	354.25	400.26	383.23	382.24	I	1126.53	1184.61	1167.58	1166.60	10
5	30.03	429.28	412.26	411.27	457.28	440.25	439.27	G		1071.52	1054.50	1053.51	9
6	136.08	592.35	575.32	574.33	620.34	603.31	602.33	Y	906.44	1014.50	997.47	996.49	8
7	86.10	705.43	688.40	687.42	733.42	716.40	715.41	L	793.36	851.44	834.41	833.43	7
8	87.06	819.47	802.45	801.46	847.47	830.44	829.46	N	679.32	738.35	721.33	720.34	6
9	74.06	920.52	903.49	902.51	948.51	931.49	930.50	T	578.27	**624.31**	607.28	606.30	5
10	30.03	977.54	960.51	959.53	1005.54	988.51	987.53	G		**523.26**	506.24		4
11	136.08	1140.60	1123.58	1122.59	1168.60	1151.57	1150.59	Y	358.18	466.24	449.21		3
12	101.07	1268.66	1251.64	1250.65	1296.66	1279.63	1278.65	Q	230.12	303.18	286.15		2
13	129.11							R	74.02	**175.12**	158.09		1

Spot 2618 analyzed by MS/MS ion search

Supplementary Figure 4H

Match to: **gi|4507953** Score: **73**
14-3-3 protein zeta/delta [Homo sapiens]
Found in search of DATA.TXT

Nominal mass (M_r): **27899**; Calculated pI value: **4.73**
NCBI BLAST search of gi|4507953 against nr
Unformatted sequence string for pasting into other applications

Taxonomy: Homo sapiens

Fixed modifications: Carbamidomethyl (C)
Variable modifications: Oxidation (M)
Cleavage by Trypsin: cuts C-term side of KR unless next residue is P
Sequence Coverage: **14%**

Matched peptides shown in **Bold Red**

```
  1 MDKNELVQKA KLAEQAERYD DMAACMKSVT EQGAELSNEE RNLLSVAYKN
 51 VVGARRSSWR VVSSIEQKTE GAEKKQQMAR EYREKIETEL RDICNDVLSL
101 LEKFLIPNAS QAESKVFYLK MKGDYYRYLA EVAAGDDKKG IVDQSQQAYQ
151 EAFEISKKEM QPTHPIRLGL ALNFSVFYYE ILNSPEKACS LAKTAFDEAI
201 AELDTLSEES YKDSTLIMQL LRDNLTLWTS DTQGDEAEAG EGGEN
```

MS/MS Fragmentation of **MKGDYYR**
Found in **gi|4507953**, 14-3-3 protein zeta/delta [Homo sapiens]

Match to Query 2: 947.402017 from(948.409293,1+)
From data file DATA.TXT

Click mouse within plot area to zoom in by factor of two about that point
Or, Plot from 50 to 950 Da

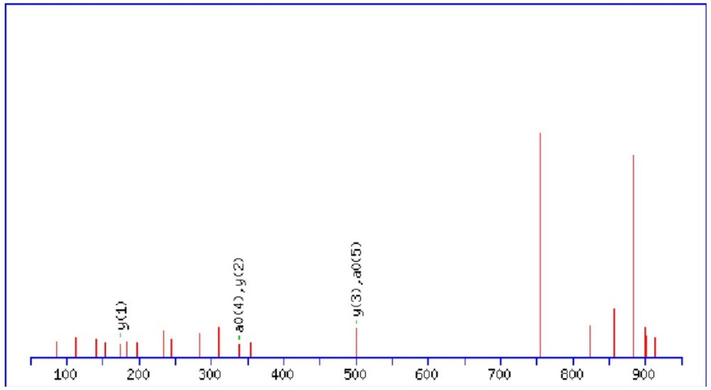

Monoisotopic mass of neutral peptide Mr(calc): 947.42
Fixed modifications: Carbamidomethyl (C)
Variable modifications:
M1 : Oxidation (M)
Ions Score: 0 **Expect:** 2.4e+002
Matches (Bold Red): 5/75 fragment ions using 21 most intense peaks

#	Immon.	a	a*	a^0	b	b*	b^0	Seq.	v	y	y*	y^0	#
1	56.05	56.05			84.04			M					7
2	101.11	184.14	167.12		212.14	195.11		K	728.30	801.39	784.36	783.38	6
3	30.03	241.17	224.14		269.16	252.13		G		673.29	656.27	655.28	5
4	88.04	356.19	339.17	**338.18**	384.19	367.16	366.18	D	556.25	616.27	599.25	598.26	4
5	136.08	519.26	502.23	**501.25**	547.25	530.22	529.24	Y	393.19	**501.25**	484.22		3
6	136.08	682.32	665.29	664.31	710.31	693.29	692.30	Y	230.12	**338.18**	321.16		2
7	129.11							R	74.02	**175.12**	158.09		1

Spot 2251 analyzed by MS/MS ion search

MS/MS Fragmentation of **NLLSVAYKNVVGAR**
Found in **gi|4507953**, 14-3-3 protein zeta/delta [Homo sapiens]

Match to Query 6: 1502.841218 from(1503.848494,1+)
From data file DATA.TXT

Click mouse within plot area to zoom in by factor of two about that point
Or, Plot from 0 to 1400 Da

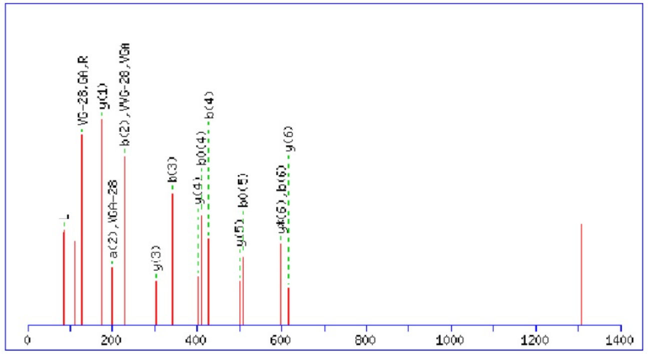

Monoisotopic mass of neutral peptide Mr(calc): 1502.86
Fixed modifications: Carbamidomethyl (C)
Ions Score: 52 **Expect:** 0.0011
Matches (Bold Red): 21/217 fragment ions using 15 most intense peaks

#	Immon.	a	a*	a^0	b	b*	b^0	Seq.	v	y	y*	y^0	#
1	87.06	87.06	70.03		115.05	98.02		N					14
2	**86.10**	**200.14**	183.11		**228.13**	211.11		L	1331.74	1389.82	1372.79	1371.81	13
3	**86.10**	313.22	296.20		**341.22**	324.19		L	1218.66	1276.74	1259.71	1258.73	12
4	60.04	400.26	383.23	382.24	**428.25**	411.22	**410.24**	S	1131.63	1163.65	1146.63	1145.64	11
5	72.08	499.32	482.30	481.31	527.32	510.29	**509.31**	V	1032.56	1076.62	1059.59		10
6	44.05	570.36	553.33	552.35	**598.36**	581.33	580.35	A	961.52	977.55	960.53		9
7	136.08	733.42	716.40	715.41	761.42	744.39	743.41	Y	798.46	906.52	889.49		8
8	101.11	861.52	844.49	843.51	889.51	872.49	871.50	K	670.36	743.45	726.43		7
9	87.06	975.56	958.54	957.55	1003.56	986.53	985.55	N	556.32	**615.36**	**598.33**		6
10	72.08	1074.63	1057.60	1056.62	1102.63	1085.60	1084.61	V	457.25	**501.31**	484.29		5
11	72.08	1173.70	1156.67	1155.69	1201.69	1184.67	1183.68	V	358.18	**402.25**	385.22		4
12	30.03	1230.72	1213.69	1212.71	1258.72	1241.69	1240.70	G		**303.18**	286.15		3
13	44.05	1301.76	1284.73	1283.75	1329.75	1312.73	1311.74	A	230.12	246.16	229.13		2
14	**129.11**							R	74.02	**175.12**	158.09		1

Supplementary Figure 4H

MS/MS Fragmentation of **SVTEQGAELSNEER**
Found in **gi|4507953**, 14-3-3 protein zeta/delta [Homo sapiens]

Match to Query 7: 1547.678392 from(1548.685668,1+)
From data file DATA.TXT

Click mouse within plot area to zoom in by factor of two about that point
Or, Plot from 0 to 1400 Da

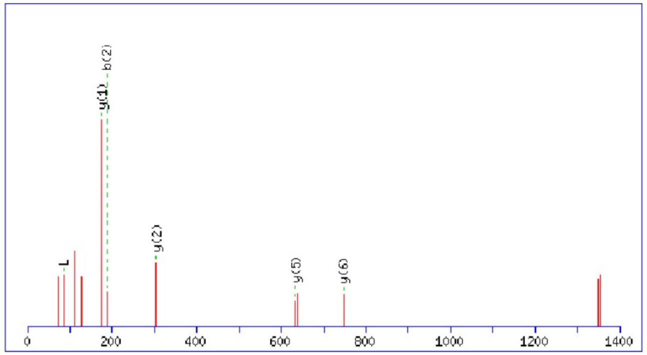

Monoisotopic mass of neutral peptide Mr(calc): 1547.71
Fixed modifications: Carbamidomethyl (C)
Ions Score: 21 **Expect:** 1.3
Matches (Bold Red): 6/227 fragment ions using 10 most intense peaks

#	Immon.	a	a*	a^0	b	b*	b^0	Seq.	v	y	y*	y^0	#
1	60.04	60.04		42.03	88.04		70.03	S					14
2	72.08	159.11		141.10	**187.11**		169.10	V	1417.62	1461.68	1444.66	1443.67	13
3	74.06	260.16		242.15	288.16		270.14	T	1316.57	1362.61	1345.59	1344.60	12
4	102.05	389.20		371.19	417.20		399.19	E	1187.53	1261.57	1244.54	1243.55	11
5	101.07	517.26	500.24	499.25	545.26	528.23	527.25	Q	1059.47	1132.52	1115.50	1114.51	10
6	30.03	574.28	557.26	556.27	602.28	585.25	584.27	G		1004.46	987.44	986.45	9
7	44.05	645.32	628.29	627.31	673.32	656.29	655.30	A	931.41	947.44	930.42	929.43	8
8	102.05	774.36	757.34	756.35	802.36	785.33	784.35	E	802.37	876.41	859.38	858.40	7
9	**86.10**	887.45	870.42	869.44	915.44	898.42	897.43	L	689.28	**747.36**	730.34	729.35	6
10	60.04	974.48	957.45	956.47	1002.47	985.45	984.46	S	602.25	**634.28**	617.25	616.27	5
11	87.06	1088.52	1071.50	1070.51	1116.52	1099.49	1098.51	N	488.21	547.25	530.22	529.24	4
12	102.05	1217.56	1200.54	1199.55	1245.56	1228.53	1227.55	E	359.17	433.20	416.18	415.19	3
13	102.05	1346.61	1329.58	1328.60	1374.60	1357.58	1356.59	E	230.12	**304.16**	287.13	286.15	2
14	129.11							R	74.02	**175.12**	158.09		1

Spot 2251 analyzed by MS/MS ion search

Supplementary Figure 4I

Match to: **gi|67190748** Score: **418**
complement C4-A isoform 1 preproprotein [Homo sapiens]
Found in search of DATA.TXT

Nominal mass (M_r): **194261**; Calculated pI value: **6.65**
NCBI BLAST search of gi|67190748 against nr
Unformatted sequence string for pasting into other applications

Taxonomy: Homo sapiens

Fixed modifications: Carbamidomethyl (C)
Variable modifications: Oxidation (M)
Cleavage by Trypsin: cuts C-term side of KR unless next residue is P
Sequence Coverage: **6%**

Matched peptides shown in **Bold Red**

1 MRLLWGLIWA SSFFTLSLQK PRLLLFSPSV VHLGVPLSVG VQLQDVPRGQ
51 VVKGSVFLRN PSRNNVPCSP KVDFTLSSER DFALLSLQVP LKDAKSCGLH
101 QLLRGPEVQL VAHSPWLKDS LSRTTNIQGI NLLFSSRRGH LFLQTDQPIY
151 NPGQRVRYRV FALDQKMRPS TDTITVMVEN SHGLRVRKKE VYMPSSIFQD
201 DFVIPDISEP GTWKISARFS DGLESNSSTQ FEVKKYVLPN FEVKITPGKP
251 YILTVPGHLD EMQLDIQARY IYGKPVQGVA YVRFGLLDED GKKTFFRGLE
301 SQTKLVNGQS HISLSKAEFQ DALEKLNMGI TDLQGLRLYV AAAIIESPGG
351 EMEEAELTSW YFVSSPFSLD LSKTKRHLVP GAPFLLQALV REMSGSPASG
401 IPVKVSATVS SPGSVPEVQD IQQNTDGSGQ VSIPIIIPQT ISELQLSVSA
451 GSPHPAIARL TVAAPPSGGP GFLSIERPDS RPPRVGDTLN LNLRAVGSGA
501 TFSHYYYMIL SRGQIVFMNR EPKRTLTSVS VFVDHHLAPS FYFVAFYYHG
551 DHPVANSLRV DVQAGACEGK LELSVDGAKQ YRNGESVKLH LETDSLALVA
601 LGALDTALYA AGSKSHKPLN MGKVFEAMNS YDLGCGPGGG DSALQVFQAA
651 GLAFSDGDQW TLSRKRLSCP KEKTTRKKRN VNFQKAINEK **LGQYASPTAK**
701 R**CCQDGVTR**L PMMRSCEQRA AR**VQQPDCR**E PFLSCCQFAE SLRKKSR**DKG**
751 **QAGLQR**ALEI LQEEDLIDED DIPVR**SFFPE** **NWLWR**VETVD RFQILTLWLP
801 DSLTTWEIHG LSLSK**TKGLC** **VATPVQLRVF** **REFHLHLRLP** **MSVR**RFEQLE
851 LRPVLYNYLD KNLTVSVHVS PVEGLCLAGG GGLAQQVLVP AGSARPVAFS
901 VVPTAAAAVS LKVVARGSFE FPVGDAVSKV LQIEKEGAIH REELVYELNP
951 LDHRGRTLEI PGNSDPNMIP DGDFNSYVRV TASDPLDTLG SEGALSPGGV
1001 ASLLRLPRGC GEQTMIYLAP TLAASRYLDK TEQWSTLPPE TKDHAVDLIQ
1051 KGYMR**IQQFR** **KADGSYAAWL** **SR**DSSTWLTA FVLKVLSLAQ EQVGGSPEKL
1101 QETSNWLLSQ QQADGSFQDP CPVLDRSMQG GLVGNDETVA LTAFVTIALH
1151 HGLAVFQDEG AEPLKQRVEA SISKANSFLG EKASAGLLGA HAAAITAYAL
1201 TLTKAPVDLL GVAHNNLMAM AQETGDNLYW GSVTGSQSNA VSPTPAPRNP
1251 SDPMPQAPAL WIETTAYALL HLLLHEGKAE MADQASAWLT R**QGSFQGGFR**
1301 STQDTVIALD ALSAYWIASH TTEERGLNVT LSSTGRNGFK **SHALQLNNR**Q
1351 IRGLEEELQF SLGSKINVKV GGNSKGTLKV LRTYNVLDMK NTTCQDLQIE
1401 VTVKGHVEYT MEANEDYEDY EYDELPAKDD PDAPLQPVTP LQLFEGRRNR
1451 RRREAPKVVE EQESRVHYTV CIWRNGKVGL SGMAIADVTL LSGFHALRAD
1501 LEKLTSLSDR YVSHFETEGP HVLLYFDSVP TSRECVGFEA VQEVPVGLVQ
1551 PASATLYDYY NPERRCSVFY GAPSKSRLLA TLCSAEVCQC AEGKCPRQRR
1601 ALERGLQDED GYRMKFACYY PRVEYGFQVK VLREDSRAAF RLFETKITQV
1651 LHFTKDVKAA ANQMRNFLVR ASCRLRLEPG KEYLIMGLDG ATYDLEGHPQ
1701 YLLDSNSWIE EMPSERLCRS TRQRAACAQL NDFLQEYGTQ GCQV

MS/MS Fragmentation of **IQQFR**
Found in **gi|67190748**, complement C4-A isoform 1 preproprotein [Homo sapiens]

Match to Query 1: 690.414656 from(691.421932,1+)
From data file DATA.TXT

Click mouse within plot area to zoom in by factor of two about that point
Or, Plot from 50 to 700 Da

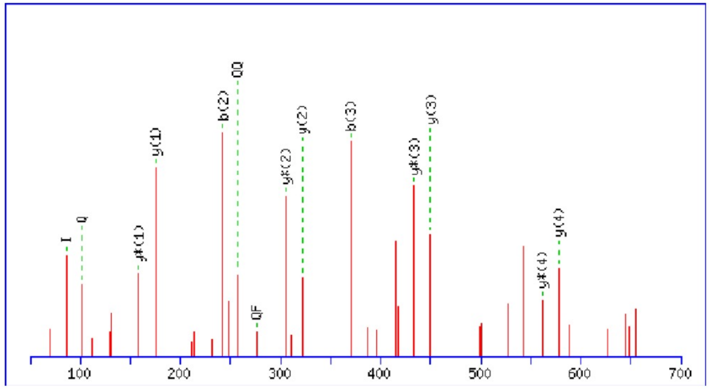

Monoisotopic mass of neutral peptide Mr(calc): 690.38
Fixed modifications: Carbamidomethyl (C)
Ions Score: 32 **Expect:** 0.28
Matches (Bold Red): 16/37 fragment ions using 18 most intense peaks

#	Immon.	a	a*	b	b*	Seq.	y	y	y*	#
1	**86.10**	**86.10**		114.09		I				5
2	**101.07**	214.16	197.13	**242.15**	225.12	Q	505.25	**578.30**	**561.28**	4
3	**101.07**	342.21	325.19	**370.21**	353.18	Q	377.19	**450.25**	**433.22**	3
4	120.08	489.28	472.26	517.28	500.25	F	230.12	**322.19**	**305.16**	2
5	129.11					R	74.02	**175.12**	**158.09**	1

Spot C3 analyzed by MS/MS ion search

Supplementary Figure 4I

MS/MS Fragmentation of **LPMSVR**
Found in **gi|67190748**, complement C4-A isoform 1 preproprotein [Homo sapiens]

Match to Query 3: 717.406316 from(718.413592,1+)
From data file DATA.TXT

Click mouse within plot area to zoom in by factor of two about that point
Or, Plot from 50 to 700 Da

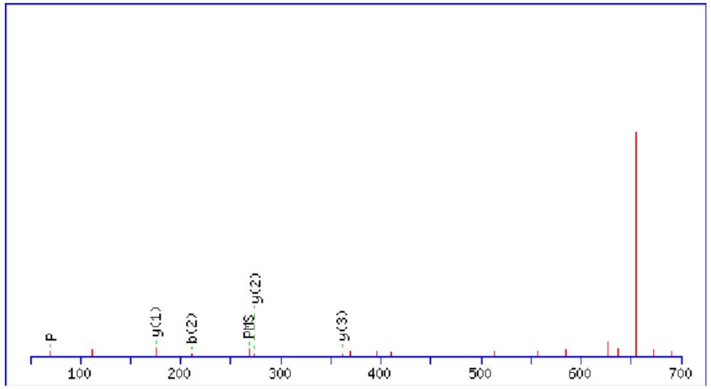

Monoisotopic mass of neutral peptide Mr(calc): 717.38
Fixed modifications: Carbamidomethyl (C)
Variable modifications:
M3 : Oxidation (M)
Ions Score: 14 **Expect:** 18
Matches (Bold Red): 6/50 fragment ions using 18 most intense peaks

#	Immon.	a	a^0	b	b^0	Seq.	v	y	y*	y^0	#
1	86.10	86.10		114.09		L					6
2	**70.07**	183.15		**211.14**		P	499.26	541.31	524.28	523.30	5
3	56.05	266.19		294.18		M	416.23	444.26	427.23	426.25	4
4	60.04	353.22	335.21	381.21	363.20	S	329.19	**361.22**	344.19	343.21	3
5	72.08	452.29	434.28	480.28	462.27	V	230.12	**274.19**	257.16		2
6	129.11					R	74.02	**175.12**	158.09		1

MS/MS Fragmentation of **VQQPDCR**
Found in **gi|67190748**, complement C4-A isoform 1 preproprotein [Homo sapiens]

Match to Query 11: 901.396076 from(902.403352,1+)
From data file DATA.TXT

Click mouse within plot area to zoom in by factor of two about that point
Or, Plot from 100 to 900 Da

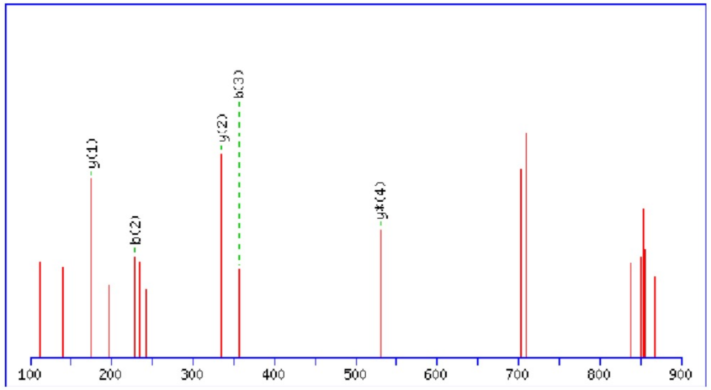

Monoisotopic mass of neutral peptide Mr(calc): 901.41
Fixed modifications: Carbamidomethyl (C)
Ions Score: 10 **Expect:** 33
Matches (Bold Red): 5/75 fragment ions using 11 most intense peaks

#	Immon.	a	a*	a^0	b	b*	b^0	Seq.	v	y	y*	y^0	#
1	72.08	72.08			100.08			V					7
2	101.07	200.14	183.11		**228.13**	211.11		Q	730.29	803.35	786.32	785.34	6
3	101.07	328.20	311.17		**356.19**	339.17		Q	602.24	675.29	658.26	657.28	5
4	70.07	425.25	408.22		453.25	436.22		P	505.18	547.23	**530.20**	529.22	4
5	88.04	540.28	523.25	522.27	568.27	551.25	550.26	D	390.16	450.18	433.15	432.17	3
6	133.04	700.31	683.28	682.30	728.30	711.28	710.29	C	230.12	**335.15**	318.12		2
7	129.11							R	74.02	**175.12**	158.09		1

Spot C3 analyzed by MS/MS ion search

Supplementary Figure 4I

MS/MS Fragmentation of **EFHLHLR**
Found in **gi|67190748**, complement C4-A isoform 1 preproprotein [Homo sapiens]

Match to Query 12: 950.486094 from(951.493370,1+)
From data file DATA.TXT

Click mouse within plot area to zoom in by factor of two about that point
Or, Plot from 50 to 950 Da

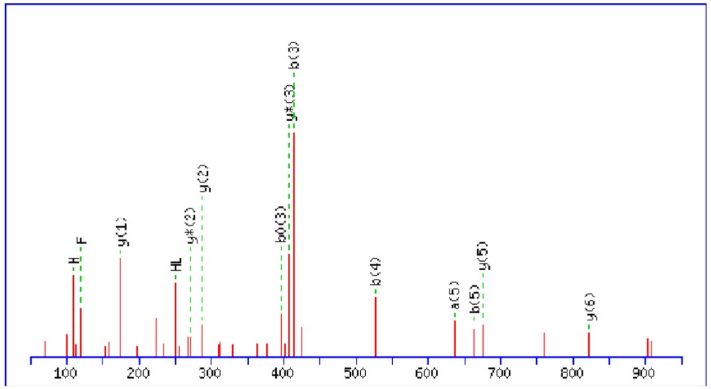

Monoisotopic mass of neutral peptide Mr(calc): 950.51
Fixed modifications: Carbamidomethyl (C)
Ions Score: 35 **Expect:** 0.11
Matches (Bold Red): 17/69 fragment ions using 18 most intense peaks

#	Immon.	a	a^0	b	b^0	Seq.	v	y	y*	#
1	102.05	102.05	84.04	130.05	112.04	E				7
2	**120.08**	249.12	231.11	277.12	259.11	F	730.41	**822.47**	805.45	6
3	**110.07**	386.18	368.17	**414.18**	**396.17**	H	593.35	**675.40**	658.38	5
4	86.10	499.27	481.26	**527.26**	509.25	L	480.27	538.35	521.32	4
5	**110.07**	**636.33**	618.31	**664.32**	646.31	H	343.21	425.26	**408.24**	3
6	86.10	749.41	731.40	777.40	759.39	L	230.12	**288.20**	**271.18**	2
7	129.11					R	74.02	**175.12**	158.09	1

MS/MS Fragmentation of **DKGQAGLQR**
Found in **gi|67190748**, complement C4-A isoform 1 preproprotein [Homo sapiens]

Match to Query 14: 971.492143 from(972.499419,1+)
From data file DATA.TXT

Click mouse within plot area to zoom in by factor of two about that point
Or, Plot from 50 to 900 Da

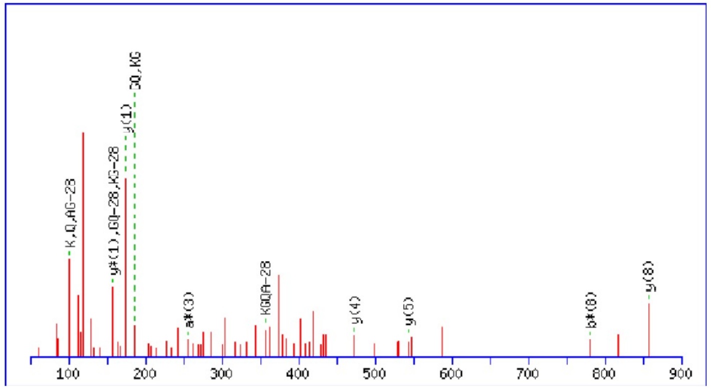

Monoisotopic mass of neutral peptide Mr(calc): 971.51
Fixed modifications: Carbamidomethyl (C)
Ions Score: 12 **Expect:** 26
Matches (Bold Red): 16/119 fragment ions using 26 most intense peaks

#	Immon.	a	a*	a^0	b	b*	b^0	Seq.	v	y	y*	#
1	88.04	88.04		70.03	116.03		98.02	D				9
2	**101.11**	216.13	199.11	198.12	244.13	227.10	226.12	K	784.41	**857.50**	840.47	8
3	30.03	273.16	**256.13**	255.15	301.15	284.12	283.14	G		729.40	712.37	7
4	**101.07**	401.21	384.19	383.20	429.21	412.18	411.20	Q	599.33	672.38	655.35	6
5	44.05	472.25	455.22	454.24	500.25	483.22	482.24	A	528.29	**544.32**	527.29	5
6	30.03	529.27	512.25	511.26	557.27	540.24	539.26	G		**473.28**	456.26	4
7	86.10	642.36	625.33	624.35	670.35	653.33	652.34	L	358.18	416.26	399.24	3
8	**101.07**	770.42	753.39	752.40	798.41	**781.38**	780.40	Q	230.12	303.18	286.15	2
9	129.11							R	74.02	**175.12**	**158.09**	1

Spot C3 analyzed by MS/MS ion search

Supplementary Figure 4I

MS/MS Fragmentation of **QGSFQGGFR**
Found in **gi|67190748**, complement C4-A isoform 1 preproprotein [Homo sapiens]

Match to Query 16: 982.439281 from(983.446557,1+)
From data file DATA.TXT

Click mouse within plot area to zoom in by factor of two about that point
Or, Plot from 50 to 1000 Da

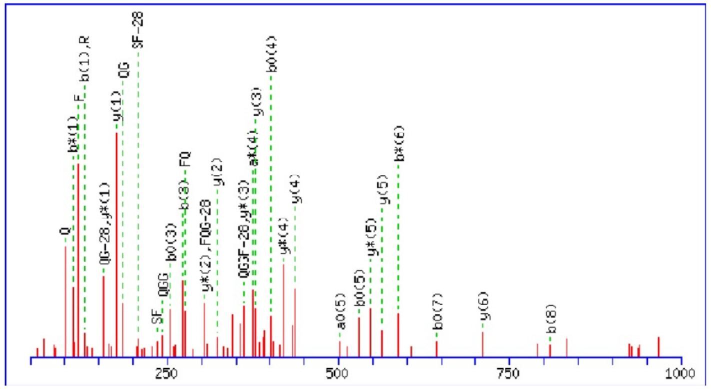

Monoisotopic mass of neutral peptide Mr(calc): 982.46
Fixed modifications: Carbamidomethyl (C)
Ions Score: 49 **Expect:** 0.0031
Matches (Bold Red): 39/118 fragment ions using 41 most intense peaks

#	Immon.	a	a*	a⁰	b	b*	b⁰	Seq.	v	y	y*	y⁰	#
1	**101.07**	**101.07**	84.04		**129.07**	**112.04**		Q					9
2	30.03	**158.09**	141.07		**186.09**	169.06		G		855.41	838.38	837.40	8
3	60.04	245.12	228.10	227.11	**273.12**	256.09	**255.11**	S	766.36	798.39	781.36	780.38	7
4	**120.08**	392.19	**375.17**	374.18	420.19	403.16	**402.18**	F	619.29	**711.36**	694.33		6
5	**101.07**	520.25	503.22	**502.24**	548.25	531.22	**530.24**	Q	491.24	**564.29**	**547.26**		5
6	30.03	577.27	560.25	559.26	605.27	**588.24**	587.26	G		**436.23**	**419.20**		4
7	30.03	634.29	617.27	616.28	662.29	645.26	**644.28**	G		**379.21**	**362.18**		3
8	**120.08**	781.36	764.34	763.35	**809.36**	792.33	791.35	F	230.12	**322.19**	**305.16**		2
9	**129.11**							R	74.02	**175.12**	**158.09**		1

MS/MS Fragmentation of **CCQDGVTR**
Found in **gi|67190748**, complement C4-A isoform 1 preproprotein [Homo sapiens]

Match to Query 18: 994.376936 from(995.384212,1+)
From data file DATA.TXT

Click mouse within plot area to zoom in by factor of two about that point
Or, Plot from 50 to 1000 Da

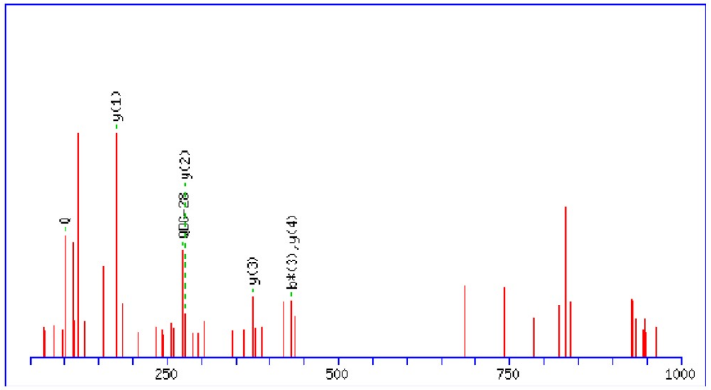

Monoisotopic mass of neutral peptide Mr(calc): 994.40
Fixed modifications: Carbamidomethyl (C)
Ions Score: 15 **Expect:** 3.8
Matches (Bold Red): 7/96 fragment ions using 14 most intense peaks

#	Immon.	a	a*	a⁰	b	b*	b⁰	Seq.	v	y	y*	y⁰	#
1	133.04	133.04			161.04			C					8
2	133.04	293.07			321.07			C	730.35	835.37	818.35	817.36	7
3	**101.07**	421.13	404.11		449.13	**432.10**		Q	602.29	675.34	658.32	657.33	6
4	88.04	536.16	519.13	518.15	564.15	547.13	546.14	D	487.26	547.28	530.26	529.27	5
5	30.03	593.18	576.15	575.17	621.18	604.15	603.16	G		**432.26**	415.23	414.25	4
6	72.08	692.25	675.22	674.24	720.24	703.22	702.23	V	331.17	**375.24**	358.21	357.22	3
7	74.06	793.30	776.27	775.29	821.29	804.27	803.28	T	230.12	**276.17**	259.14	258.16	2
8	129.11							R	74.02	**175.12**	158.09		1

Spot C3 analyzed by MS/MS ion search

Supplementary Figure 4I

MS/MS Fragmentation of **LGQYASPTAK**
Found in **gi|67190748**, complement C4-A isoform 1 preproprotein [Homo sapiens]

Match to Query 21: 1034.508013 from(1035.515289,1+)
From data file DATA.TXT

Click mouse within plot area to zoom in by factor of two about that point
Or, Plot from 50 to 1000 Da

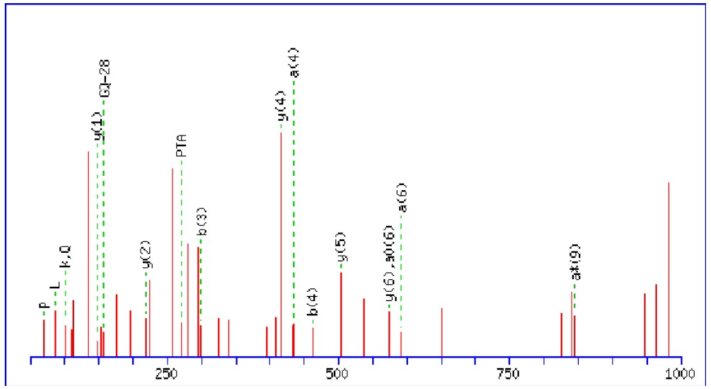

Monoisotopic mass of neutral peptide Mr(calc): 1034.54
Fixed modifications: Carbamidomethyl (C)
Ions Score: 17 **Expect:** 6.8
Matches (Bold Red): 18/127 fragment ions using 37 most intense peaks

#	Immon.	a	a*	a^0	b	b*	b^0	Seq.	y	y*	y^0	#
1	**86.10**	**86.10**			114.09			L				10
2	30.03	143.12			171.11			G	922.46	905.44	904.45	9
3	**101.07**	271.18	254.15		**299.17**	282.14		Q	865.44	848.41	847.43	8
4	136.08	**434.24**	417.21		**462.23**	445.21		Y	737.38	720.36	719.37	7
5	44.05	505.28	488.25		533.27	516.25		A	**574.32**	557.29	556.31	6
6	60.04	**592.31**	575.28	**574.30**	620.30	603.28	602.29	S	**503.28**	486.26	485.27	5
7	**70.07**	689.36	672.34	671.35	717.36	700.33	699.35	P	**416.25**	399.22	398.24	4
8	74.06	790.41	773.38	772.40	818.40	801.38	800.39	T	319.20	302.17	301.19	3
9	44.05	861.45	**844.42**	843.44	889.44	872.41	871.43	A	**218.15**	201.12		2
10	**101.11**							K	**147.11**	130.09		1

MS/MS Fragmentation of **SHALQLNNR**
Found in **gi|67190748**, complement C4-A isoform 1 preproprotein [Homo sapiens]

Match to Query 23: 1051.524256 from(1052.531532,1+)
From data file DATA.TXT

Click mouse within plot area to zoom in by factor of two about that point
Or, Plot from 50 to 1050 Da

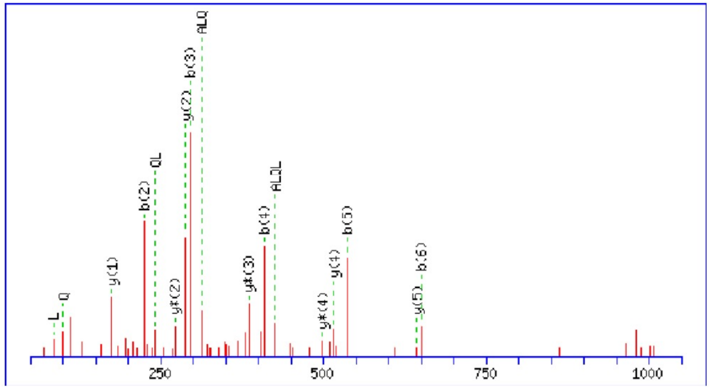

Monoisotopic mass of neutral peptide Mr(calc): 1051.55
Fixed modifications: Carbamidomethyl (C)
Ions Score: 40 **Expect:** 0.029
Matches (Bold Red): 19/113 fragment ions using 24 most intense peaks

#	Immon.	a	a*	a^0	b	b*	b^0	Seq.	v	y	y*	#
1	60.04	60.04		42.03	88.04		70.03	S				9
2	110.07	197.10		179.09	**225.10**		207.09	H	883.47	965.53	948.50	8
3	44.05	268.14		250.13	**296.14**		278.12	A	812.44	828.47	811.44	7
4	**86.10**	381.22		363.21	**409.22**		391.21	L	699.35	757.43	740.40	6
5	**101.07**	509.28	492.26	491.27	**537.28**	520.25	519.27	Q	571.29	**644.35**	627.32	5
6	**86.10**	622.37	605.34	604.36	**650.36**	633.34	632.35	L	458.21	**516.29**	**499.26**	4
7	87.06	736.41	719.38	718.40	764.40	747.38	746.39	N	344.17	403.20	**386.18**	3
8	87.06	850.45	833.43	832.44	878.45	861.42	860.44	N	230.12	**289.16**	**272.14**	2
9	129.11							R	74.02	**175.12**	158.09	1

Spot C3 analyzed by MS/MS ion search

Supplementary Figure 4I

MS/MS Fragmentation of **ADGSYAAWLSR**
Found in **gi|67190748**, complement C4-A isoform 1 preproprotein [Homo sapiens]

Match to Query 29: 1195.529314 from(1196.536590,1+)
From data file DATA.TXT

Click mouse within plot area to zoom in by factor of two about that point
Or, Plot from 0 to 1200 Da

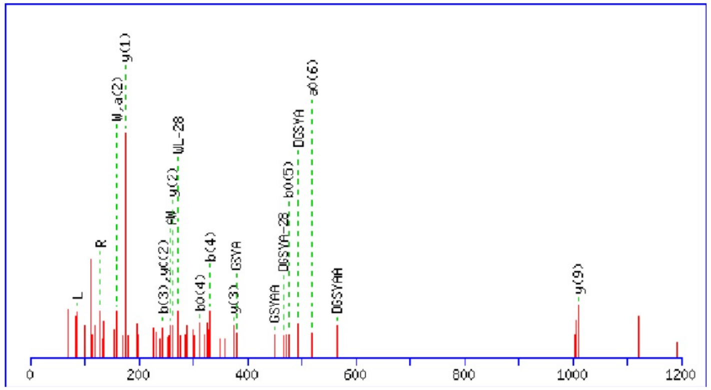

Monoisotopic mass of neutral peptide Mr(calc): 1195.56
Fixed modifications: Carbamidomethyl (C)
Ions Score: 36 **Expect:** 0.059
Matches (Bold Red): 24/147 fragment ions using 29 most intense peaks

#	Immon.	a	a^0	b	b^0	Seq.	v	y	y*	y^0	#
1	44.05	44.05		72.04		A					11
2	88.04	**159.08**	141.07	187.07	169.06	D	1065.51	1125.53	1108.51	1107.52	10
3	30.03	216.10	198.09	**244.09**	226.08	G		**1010.51**	993.48	992.49	9
4	60.04	303.13	285.12	**331.12**	**313.11**	S	921.46	953.48	936.46	935.47	8
5	136.08	**466.19**	448.18	**494.19**	**476.18**	Y	758.39	866.45	849.43	848.44	7
6	44.05	537.23	**519.22**	**565.23**	547.21	A	687.36	703.39	686.36	685.38	6
7	44.05	608.27	590.26	636.26	618.25	A	616.32	632.35	615.32	614.34	5
8	**159.09**	794.35	776.34	822.34	804.33	W	430.24	561.31	544.29	543.30	4
9	**86.10**	907.43	889.42	935.43	917.42	L	317.16	**375.24**	358.21	357.22	3
10	60.04	994.46	976.45	1022.46	1004.45	S	230.12	**262.15**	245.12	**244.14**	2
11	**129.11**					R	74.02	**175.12**	158.09		1

MS/MS Fragmentation of **GLCVATPVQLR**
Found in **gi|67190748**, complement C4-A isoform 1 preproprotein [Homo sapiens]

Match to Query 31: 1212.634741 from(1213.642017,1+)
From data file DATA.TXT

Click mouse within plot area to zoom in by factor of two about that point
Or, Plot from 0 to 1200 Da

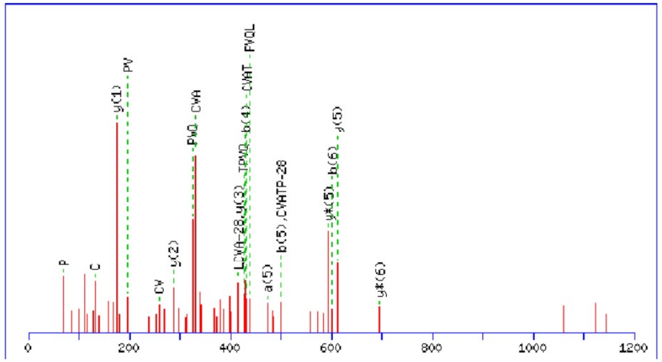

Monoisotopic mass of neutral peptide Mr(calc): 1212.66
Fixed modifications: Carbamidomethyl (C)
Ions Score: 34 **Expect:** 0.094
Matches (Bold Red): 22/141 fragment ions using 24 most intense peaks

#	Immon.	a	a*	a^0	b	b*	b^0	Seq.	v	y	y*	y^0	#
1	30.03	30.03			58.03			G					11
2	86.10	143.12			171.11			L	1098.57	1156.65	1139.62	1138.64	10
3	**133.04**	303.15			**331.14**			C	938.54	1043.57	1026.54	1025.56	9
4	72.08	402.22			**430.21**			V	839.47	883.54	866.51	865.53	8
5	44.05	**473.25**			**501.25**			A	768.44	784.47	767.44	766.46	7
6	74.06	574.30		556.29	**602.30**		584.29	T	667.39	713.43	**696.40**	695.42	6
7	**70.07**	671.35		653.34	699.35		681.34	P	570.34	**612.38**	**595.36**		5
8	72.08	770.42		752.41	798.42		780.41	V	471.27	515.33	498.30		4
9	101.07	898.48	881.45	880.47	926.48	909.45	908.47	Q	343.21	**416.26**	399.24		3
10	86.10	1011.57	994.54	993.55	1039.56	1022.53	1021.55	L	230.12	**288.20**	271.18		2
11	129.11							R	74.02	**175.12**	158.09		1

Spot C3 analyzed by MS/MS ion search

Supplementary Figure 4I

MS/MS Fragmentation of **KADGSYAAWLSR**
Found in **gi|67190748**, complement C4-A isoform 1 preproprotein [Homo sapiens]

Match to Query 32: 1323.627593 from(1324.634869,1+)
From data file DATA.TXT

Click mouse within plot area to zoom in by factor of two about that point
Or, Plot from 0 to 1300 Da

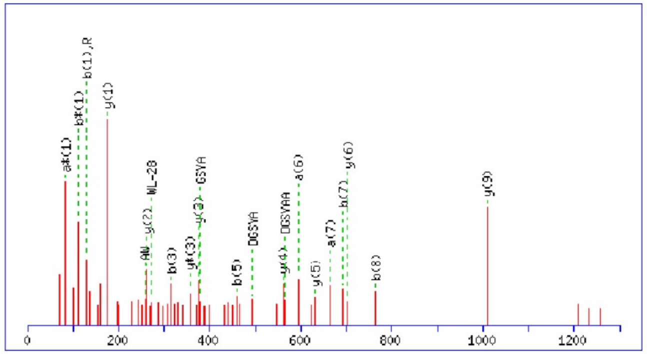

Monoisotopic mass of neutral peptide Mr(calc): 1323.66
Fixed modifications: Carbamidomethyl (C)
Ions Score: 67 **Expect:** 5.1e-005
Matches (Bold Red): 25/188 fragment ions using 25 most intense peaks

#	Immon.	a	a*	a^0	b	b*	b^0	Seq.	v	y	y*	y^0	#
1	101.11	101.11	**84.08**		**129.10**	**112.08**		K					12
2	44.05	172.14	155.12		200.14	183.11		A	1180.54	1196.57	1179.54	1178.56	11
3	88.04	287.17	270.14	269.16	**315.17**	298.14	297.16	D	1065.51	1125.53	1108.51	1107.52	10
4	30.03	344.19	327.17	326.18	372.19	355.16	354.18	G		**1010.51**	993.48	992.49	9
5	60.04	431.22	414.20	413.21	**459.22**	442.19	441.21	S	921.46	953.48	936.46	935.47	8
6	136.08	**594.29**	577.26	576.28	622.28	605.26	604.27	Y	758.39	866.45	849.43	848.44	7
7	44.05	**665.33**	648.30	647.31	**693.32**	676.29	675.31	A	687.36	**703.39**	686.36	685.38	6
8	44.05	736.36	719.34	718.35	**764.36**	747.33	746.35	A	616.32	**632.35**	615.32	614.34	5
9	159.09	922.44	905.42	904.43	950.44	933.41	932.43	W	430.24	**561.31**	544.29	543.30	4
10	86.10	1035.53	1018.50	1017.52	1063.52	1046.49	1045.51	L	317.16	**375.24**	**358.21**	357.22	3
11	60.04	1122.56	1105.53	1104.55	1150.55	1133.53	1132.54	S	230.12	**262.15**	245.12	244.14	2
12	**129.11**							R	74.02	**175.12**	158.09		1

MS/MS Fragmentation of **VFREFHLHLR**
Found in **gi|67190748**, complement C4-A isoform 1 preproprotein [Homo sapiens]

Match to Query 36: 1352.713276 from(1353.720552,1+)
From data file DATA.TXT

Click mouse within plot area to zoom in by factor of two about that point
Or, Plot from 0 to 1200 Da

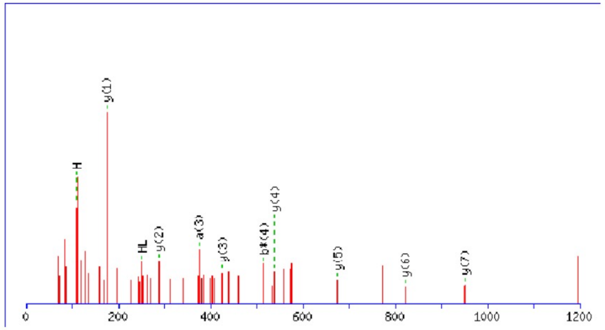

Monoisotopic mass of neutral peptide Mr(calc): 1352.75
Fixed modifications: Carbamidomethyl (C)
Ions Score: 34 **Expect:** 0.12
Matches (Bold Red): 14/131 fragment ions using 24 most intense peaks

#	Immon.	a	a*	a^0	b	b*	b^0	d	Seq.	v	y	y*	y^0	#
1	72.08	72.08			100.08				V					10
2	120.08	219.15			247.14				F	1162.62	1254.69	1237.66	1236.67	9
3	129.11	**375.25**	358.22		403.25	386.22		290.19	R	1006.52	1107.62	1090.59	1089.61	8
4	102.05	504.29	487.27	486.28	532.29	**515.26**	514.28	446.29	E	877.48	**951.52**	934.49	933.51	7
5	120.08	651.36	634.33	633.35	679.36	662.33	661.35		F	730.41	**822.47**	805.45		6
6	**110.07**	788.42	771.39	770.41	816.42	799.39	798.40		H	593.35	**675.40**	658.38		5
7	86.10	901.50	884.48	883.49	929.50	912.47	911.49	859.46	L	480.27	**538.35**	521.32		4
8	**110.07**	1038.56	1021.54	1020.55	1066.56	1049.53	1048.55		H	343.21	**425.26**	408.24		3
9	86.10	1151.65	1134.62	1133.64	1179.64	1162.62	1161.63	1109.60	L	230.12	**288.20**	271.18		2
10	129.11								R	74.02	**175.12**	158.09		1

Spot C3 analyzed by MS/MS ion search

Supplementary Figure 4I

MS/MS Fragmentation of **SFFPENWLWR**
Found in **gi|67190748**, complement C4-A isoform 1 preproprotein [Homo sapiens]

Match to Query 39: 1380.634939 from(1381.642215,1+)
From data file DATA.TXT

Click mouse within plot area to zoom in by factor of two about that point
Or, Plot from 0 to 1400 Da

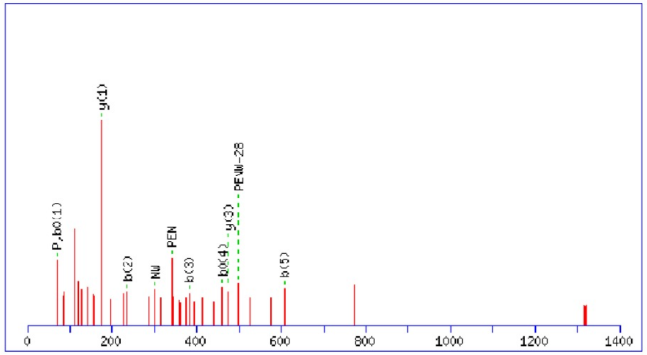

Monoisotopic mass of neutral peptide Mr(calc): 1380.66
Fixed modifications: Carbamidomethyl (C)
Ions Score: 17 **Expect:** 4.3
Matches (Bold Red): 11/127 fragment ions using 16 most intense peaks

#	Immon.	a	a*	a0	b	b*	b0	Seq.	v	y	y*	y0	#
1	60.04	60.04		42.03	88.04		**70.03**	S					10
2	120.08	207.11		189.10	**235.11**		217.10	F	1202.57	1294.64	1277.61	1276.63	9
3	120.08	354.18		336.17	**382.18**		364.17	F	1055.51	1147.57	1130.54	1129.56	8
4	**70.07**	451.23		433.22	479.23		**461.22**	P	958.45	1000.50	983.47	982.49	7
5	102.05	580.28		562.27	**608.27**		590.26	E	829.41	903.45	886.42	885.44	6
6	87.06	694.32	677.29	676.31	722.31	705.29	704.30	N	715.37	774.40	757.38		5
7	159.09	880.40	863.37	862.39	908.39	891.37	890.38	W	529.29	660.36	643.34		4
8	86.10	993.48	976.46	975.47	1021.48	1004.45	1003.47	L	416.20	**474.28**	457.26		3
9	159.09	1179.56	1162.54	1161.55	1207.56	1190.53	1189.55	W	230.12	361.20	344.17		2
10	129.11							R	74.02	**175.12**	158.09		1

MS/MS Fragmentation of **TKGLCVATPVQLR**
Found in **gi|67190748**, complement C4-A isoform 1 preproprotein [Homo sapiens]

Match to Query 42: 1441.776642 from(1442.783918,1+)
From data file DATA.TXT

Click mouse within plot area to zoom in by factor of two about that point
Or, Plot from 0 to 1400 Da

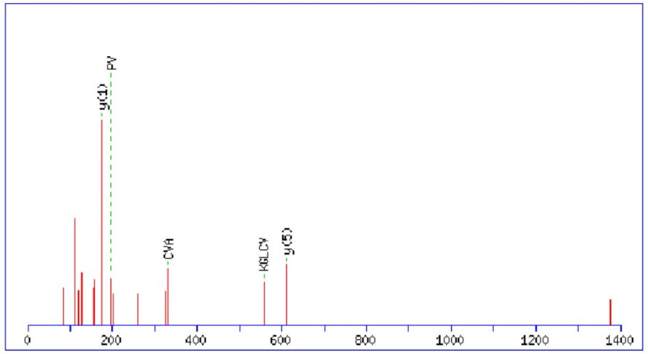

Monoisotopic mass of neutral peptide Mr(calc): 1441.81
Fixed modifications: Carbamidomethyl (C)
Ions Score: 12 **Expect:** 16
Matches (Bold Red): 6/208 fragment ions using 6 most intense peaks

#	Immon.	a	a*	a0	b	b*	b0	Seq.	v	y	y*	y0	#
1	74.06	74.06		56.05	102.05		84.04	T					13
2	101.11	202.16	185.13	184.14	230.15	213.12	212.14	K	1268.68	1341.77	1324.74	1323.76	12
3	30.03	259.18	242.15	241.17	287.17	270.14	269.16	G		1213.67	1196.65	1195.66	11
4	86.10	372.26	355.23	354.25	400.26	383.23	382.24	L	1098.57	1156.65	1139.62	1138.64	10
5	133.04	532.29	515.26	514.28	560.29	543.26	542.28	C	938.54	1043.57	1026.54	1025.56	9
6	72.08	631.36	614.33	613.35	659.35	642.33	641.34	V	839.47	883.54	866.51	865.53	8
7	44.05	702.40	685.37	684.39	730.39	713.37	712.38	A	768.44	784.47	767.44	766.46	7
8	74.06	803.44	786.42	785.43	831.44	814.41	813.43	T	667.39	713.43	696.40	695.42	6
9	70.07	900.50	883.47	882.49	928.49	911.47	910.48	P	570.34	**612.38**	595.36		5
10	72.08	999.57	982.54	981.55	1027.56	1010.53	1009.55	V	471.27	515.33	498.30		4
11	101.07	1127.62	1110.60	1109.61	1155.62	1138.59	1137.61	Q	343.21	416.26	399.24		3
12	86.10	1240.71	1223.68	1222.70	1268.70	1251.68	1250.69	L	230.12	288.20	271.18		2
13	129.11							R	74.02	**175.12**	158.09		1

Spot C3 analyzed by MS/MS ion search

Supplementary Figure 4J

Match to: **gi|31542984** Score: **454**
inter-alpha-trypsin inhibitor heavy chain H4 isoform 1 precursor [Homo sapiens]
Found in search of DATA.TXT

Nominal mass (M_r): **103521**; Calculated pI value: **6.51**
NCBI BLAST search of gi|31542984 against nr
Unformatted sequence string for pasting into other applications

Taxonomy: Homo sapiens

Fixed modifications: Carbamidomethyl (C)
Variable modifications: Oxidation (M)
Cleavage by Trypsin: cuts C-term side of KR unless next residue is P
Sequence Coverage: **16%**

Matched peptides shown in **Bold Red**

1 MKPPRPVRTC SKVLVLLSLL AIHQTTTAEK NGIDIYSLTV DSRVSSR**FAH**
51 **TVVTSR**VVNR ANTVQEATFQ MELPKKAFIT NFSMIIDGMT YPGIIKEKAE
101 AQAQYSAAVA KGKSAGLVKA TGRNMEQFQV SVSVAPNAKI TFELVYEELL
151 KR**RLGVYELL LKVRPQQLVK** HLQMDIHIFE PQGISFLETE STFMTNQLVD
201 ALTTWQNKTK AHIRFKPTLS QQQK**SPEQQE TVLDGNLIIR** YDVDRAISGG
251 SIQIENGYFV HYFAPEGLTT MPKNVVFVID KSGSMSGRKI QQTREALIK**I**
301 **LDDLSPRDQF NLIVFSTEAT QWRPSLVPAS AENVNK**ARSF AAGIQALGGT
351 NINDAMLMAV QLLDSSNQEE RLPEGSVSLI ILLTDGDPTV GETNPR**SIQN**
401 **NVR**EAVSGRY SLFCLGFGFD VSYAFLEK**LA LDNGGLAR**RI HEDSDSALQL
451 QDFYQEVANP LLTAVTFEYP SNAVEEVTQN NFRLLFKGSE MVVAGK**LQDR**
501 **GPDVLTATVS GK**LPTQNITF QTESSVAEQE AEFQSPK**YIF HNFMER**LWAY
551 LTIQQLLEQT VSASDADQQA LRNQALNLSL AYSFVTPLTS MVVTKPDDQE
601 QSQVAEKPME GESRNRNVHS GSTFFKYYLQ GAKIPKPEAS FSPRRGWNRQ
651 AGAAGSR**MNF RPGVLSSR**QL GLPGPPDVPD HAAYHPFRRL AILPASAPPA
701 TSNPDPAVSR VMNMKIEETT MTTQTPAPIQ APSAILPLPG QSVER**LCVDP**
751 **R**HRQGPVNLL SDPEQGVEVT GQYEREKAGF SWIEVTFKNP LVWVHASPEH
801 VVVTRNRRSS AYKWKETLFS VMPGLKMTMD KTGLLLLSDP DKVTIGLLFW
851 DGRGEGLRLL LRDTDRFSSH VGGTLGQFYQ EVLWGSPAAS DDGRRTLR**VQ**
901 **GNDHSATR**ER RLDYQEGPPG VEISCWSVEL

MS/MS Fragmentation of **LCVDPR**
Found in **gi|31542984**, inter-alpha-trypsin inhibitor heavy chain H4 isoform 1 precursor [Homo sapiens]

Match to Query 1: 758.396099 from(759.403375,1+)
From data file DATA.TXT

Click mouse within plot area to zoom in by factor of two about that point
Or, Plot from 50 to 750 Da

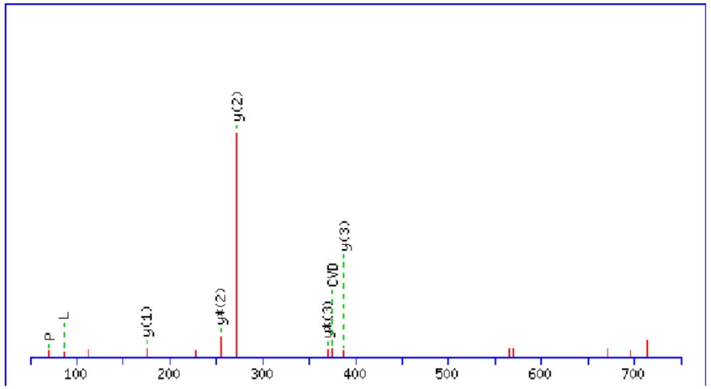

Monoisotopic mass of neutral peptide Mr(calc): 758.37
Fixed modifications: Carbamidomethyl (C)
Ions Score: 18 **Expect:** 9.9
Matches (Bold Red): 9/50 fragment ions using 15 most intense peaks

#	Immon.	a	a^0	b	b^0	Seq.	v	y	y*	y^0	#
1	**86.10**	**86.10**		114.09		L					6
2	133.04	246.13		274.12		C	541.27	646.30	629.27	628.29	5
3	72.08	345.20		373.19		V	442.20	486.27	469.24	468.26	4
4	88.04	460.22	442.21	488.22	470.21	D	327.18	**387.20**	**370.17**	369.19	3
5	**70.07**	557.28	539.26	585.27	567.26	P	230.12	**272.17**	**255.15**		2
6	129.11					R	74.02	**175.12**	158.09		1

Spot C2 analyzed by MS/MS ion search

Supplementary Figure 4J

MS/MS Fragmentation of **SIQNNVR**
Found in **gi|31542984**, inter-alpha-trypsin inhibitor heavy chain H4 isoform 1 precursor [Homo sapiens]

Match to Query 3: 829.455017 from(830.462293,1+)
From data file DATA.TXT

Click mouse within plot area to zoom in by factor of two about that point
Or, Plot from 50 to 800 Da

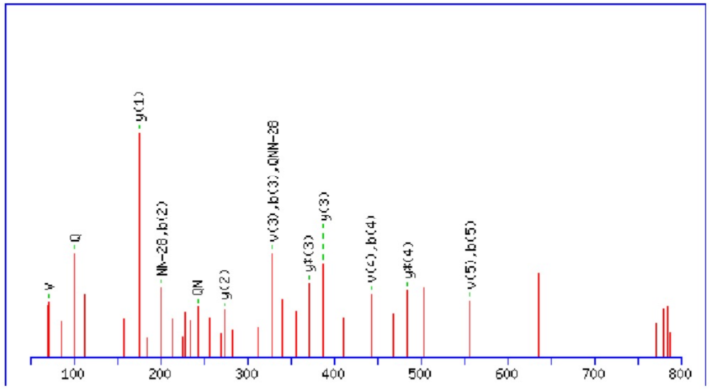

Monoisotopic mass of neutral peptide Mr(calc): 829.44
Fixed modifications: Carbamidomethyl (C)
Ions Score: 25 Expect: 1.8
Matches (**Bold Red**): 17/77 fragment ions using 19 most intense peaks

#	Immon.	a	a*	a^0	b	b*	b^0	Seq.	v	y	y*	#
1	60.04	60.04		42.03	88.04		70.03	S				7
2	86.10	173.13		155.12	**201.12**		183.11	I	685.34	743.42	726.39	6
3	**101.07**	301.19	284.16	283.18	**329.18**	312.16	311.17	Q	**557.28**	630.33	613.31	5
4	87.06	415.23	398.20	397.22	**443.22**	426.20	425.21	N	**443.24**	502.27	**485.25**	4
5	87.06	529.27	512.25	511.26	**557.27**	540.24	539.26	N	**329.19**	**388.23**	**371.20**	3
6	**72.08**	628.34	611.31	610.33	656.34	639.31	638.33	V	230.12	**274.19**	257.16	2
7	129.11							R	74.02	**175.12**	158.09	1

MS/MS Fragmentation of **ILDDLSPR**
Found in **gi|31542984**, inter-alpha-trypsin inhibitor heavy chain H4 isoform 1 precursor [Homo sapiens]

Match to Query 7: 927.505138 from(928.512414,1+)
From data file DATA.TXT

Click mouse within plot area to zoom in by factor of two about that point
Or, Plot from 50 to 900 Da

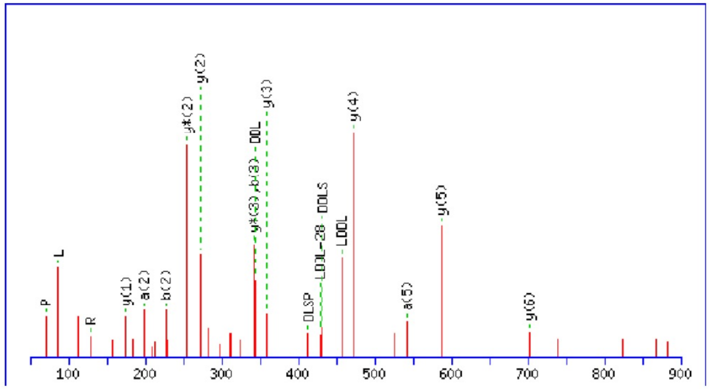

Monoisotopic mass of neutral peptide Mr(calc): 927.50
Fixed modifications: Carbamidomethyl (C)
Ions Score: 54 Expect: 0.0014
Matches (**Bold Red**): 26/88 fragment ions using 25 most intense peaks

#	Immon.	a	a^0	b	b^0	Seq.	v	y	y*	y^0	#
1	**86.10**	**86.10**		114.09		I					8
2	**86.10**	**199.18**		**227.18**		L	757.35	815.43	798.40	797.42	7
3	88.04	314.21	296.20	**342.20**	324.19	D	642.32	**702.34**	685.32	684.33	6
4	88.04	**429.23**	411.22	**457.23**	439.22	D	527.29	**587.31**	570.29	569.30	5
5	**86.10**	**542.32**	524.31	570.31	552.30	L	414.21	**472.29**	455.26	454.28	4
6	60.04	629.35	611.34	657.35	639.33	S	327.18	**359.20**	**342.18**	341.19	3
7	**70.07**	726.40	708.39	754.40	736.39	P	230.12	**272.17**	**255.15**		2
8	**129.11**					R	74.02	**175.12**	158.09		1

Spot C2 analyzed by MS/MS ion search

Supplementary Figure 4J

MS/MS Fragmentation of **VRPQQLVK**
Found in **gi|31542984**, inter-alpha-trypsin inhibitor heavy chain H4 isoform 1 precursor [Homo sapiens]

Match to Query 9: 966.596628 from(967.603904,1+)
From data file DATA.TXT

Click mouse within plot area to zoom in by factor of two about that point
Or, Plot from 50 to 1000 Da

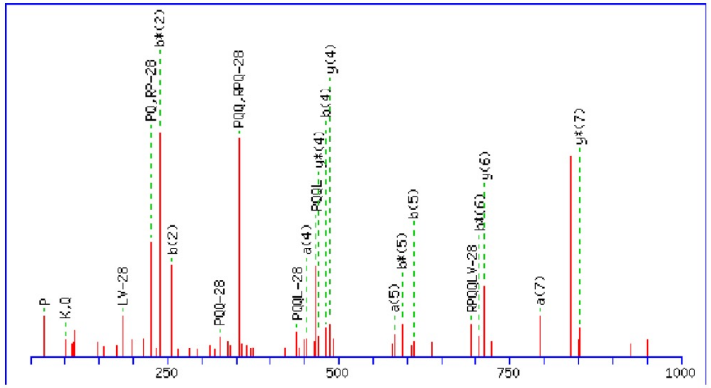

Monoisotopic mass of neutral peptide Mr(calc): 966.60
Fixed modifications: Carbamidomethyl (C)
Ions Score: 31 **Expect:** 0.11
Matches (Bold Red): 27/84 fragment ions using 27 most intense peaks

#	Immon.	a	a*	b	b*	d	Seq.	v	y	y*	#
1	72.08	72.08		100.08			V				8
2	129.11	228.18	211.16	**256.18**	**239.15**	143.12	R	767.44	868.54	**851.51**	7
3	**70.07**	325.23	308.21	353.23	336.20	299.22	P		**712.44**	695.41	6
4	**101.07**	**453.29**	436.27	**481.29**	464.26	396.27	Q		615.38	598.36	5
5	**101.07**	**581.35**	564.33	**609.35**	**592.32**	524.33	Q		**487.32**	**470.30**	4
6	86.10	**694.44**	677.41	722.43	**705.40**	652.39	L		359.27	342.24	3
7	72.08	**793.50**	776.48	821.50	804.47	779.49	V		246.18	229.15	2
8	**101.11**						K		147.11	130.09	1

MS/MS Fragmentation of **LALDNGGLAR**
Found in **gi|31542984**, inter-alpha-trypsin inhibitor heavy chain H4 isoform 1 precursor [Homo sapiens]

Match to Query 11: 998.547689 from(999.554965,1+)
From data file DATA.TXT

Click mouse within plot area to zoom in by factor of two about that point
Or, Plot from 50 to 1000 Da

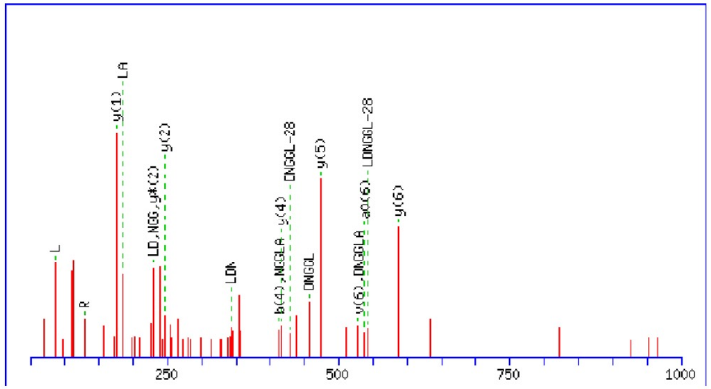

Monoisotopic mass of neutral peptide Mr(calc): 998.55
Fixed modifications: Carbamidomethyl (C)
Ions Score: 24 **Expect:** 1.4
Matches (Bold Red): 28/133 fragment ions using 33 most intense peaks

#	Immon.	a	a*	a^0	b	b*	b^0	Seq.	v	y	y*	y^0	#
1	**86.10**	**86.10**			114.09			L					10
2	44.05	157.13			**185.13**			A	870.44	886.47	869.45	868.46	9
3	**86.10**	270.22			298.21			L	757.36	815.44	798.41	797.43	8
4	88.04	385.24		367.23	**413.24**		395.23	D	642.33	702.35	685.33	684.34	7
5	87.06	499.29	482.26	481.28	527.28	510.26	509.27	N	**528.29**	**587.33**	570.30		6
6	30.03	556.31	539.28	**538.30**	584.30	567.28	566.29	G		**473.28**	456.26		5
7	30.03	613.33	596.30	595.32	641.33	624.30	623.31	G		**416.26**	399.24		4
8	**86.10**	726.41	709.39	708.40	754.41	737.38	736.40	L	301.16	359.24	342.21		3
9	44.05	797.45	780.43	779.44	825.45	808.42	807.44	A	230.12	**246.16**	**229.13**		2
10	**129.11**							R	74.02	**175.12**	158.09		1

Spot C2 analyzed by MS/MS ion search

Supplementary Figure 4J

MS/MS Fragmentation of **FAHTVVTSR**
Found in **gi|31542984**, inter-alpha-trypsin inhibitor heavy chain H4 isoform 1 precursor [Homo sapiens]

Match to Query 13: 1016.539526 from(1017.546802,1+)
From data file DATA.TXT

Click mouse within plot area to zoom in by factor of two about that point
Or, Plot from 50 to 1000 Da

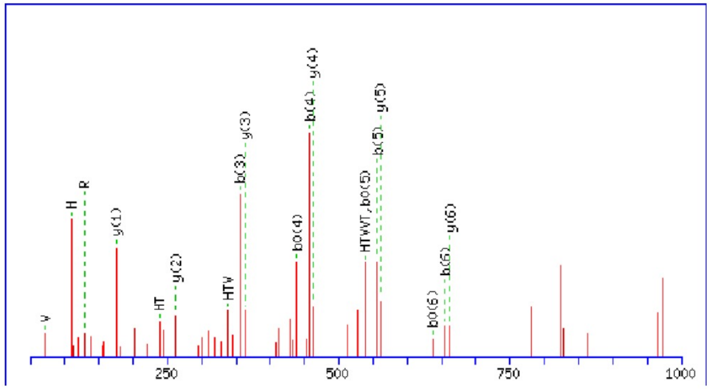

Monoisotopic mass of neutral peptide Mr(calc): 1016.54
Fixed modifications: Carbamidomethyl (C)
Ions Score: 49 **Expect:** 0.0056
Matches (Bold Red): 20/108 fragment ions using 23 most intense peaks

#	Immon.	a	a^0	b	b^0	Seq.	v	y	y*	y^0	#
1	120.08	120.08		148.08		F					9
2	44.05	191.12		219.11		A	854.45	870.48	853.45	852.47	8
3	**110.07**	328.18		**356.17**		H	717.39	799.44	782.42	781.43	7
4	74.06	429.22	411.21	**457.22**	**439.21**	T	616.34	**662.38**	645.36	644.37	6
5	**72.08**	528.29	510.28	**556.29**	**538.28**	V	517.27	**561.34**	544.31	543.32	5
6	**72.08**	627.36	609.35	**655.36**	**637.35**	V	418.20	**462.27**	445.24	444.26	4
7	74.06	728.41	710.40	756.40	738.39	T	317.16	**363.20**	346.17	345.19	3
8	60.04	815.44	797.43	843.44	825.43	S	230.12	**262.15**	245.12	244.14	2
9	**129.11**					R	74.02	**175.12**	158.09		1

MS/MS Fragmentation of **VQGNDHSATR**
Found in **gi|31542984**, inter-alpha-trypsin inhibitor heavy chain H4 isoform 1 precursor [Homo sapiens]

Match to Query 17: 1083.517530 from(1084.524806,1+)
From data file DATA.TXT

Click mouse within plot area to zoom in by factor of two about that point
Or, Plot from 50 to 1050 Da

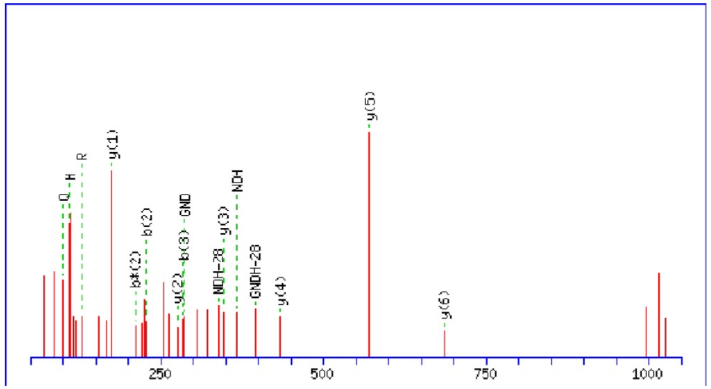

Monoisotopic mass of neutral peptide Mr(calc): 1083.51
Fixed modifications: Carbamidomethyl (C)
Ions Score: 36 **Expect:** 0.069
Matches (Bold Red): 16/141 fragment ions using 30 most intense peaks

#	Immon.	a	a*	a^0	b	b*	b^0	Seq.	v	y	y*	y^0	#
1	72.08	72.08			100.08			V					10
2	**101.07**	200.14	183.11		**228.13**	**211.11**		Q	912.39	985.44	968.42	967.43	9
3	30.03	257.16	240.13		**285.16**	268.13		G		857.39	840.36	839.38	8
4	87.06	371.20	354.18		399.20	382.17		N	741.33	800.36	783.34	782.35	7
5	88.04	486.23	469.20	468.22	514.23	497.20	496.22	D	626.30	**686.32**	669.30	668.31	6
6	**110.07**	623.29	606.26	605.28	651.28	634.26	633.27	H	489.24	**571.29**	554.27	553.28	5
7	60.04	710.32	693.30	692.31	738.32	721.29	720.31	S	402.21	**434.24**	417.21	416.23	4
8	44.05	781.36	764.33	763.35	809.35	792.33	791.34	A	331.17	**347.20**	330.18	329.19	3
9	74.06	882.41	865.38	864.40	910.40	893.37	892.39	T	230.12	**276.17**	259.14	258.16	2
10	**129.11**							R	74.02	**175.12**	158.09		1

Spot C2 analyzed by MS/MS ion search

Supplementary Figure 4J

MS/MS Fragmentation of **RLGVYELLLK**
Found in **gi|31542984**, inter-alpha-trypsin inhibitor heavy chain H4 isoform 1 precursor [Homo sapiens]

Match to Query 18: 1202.730249 from(1203.737525,1+)
From data file DATA.TXT

Click mouse within plot area to zoom in by factor of two about that point
Or, Plot from 0 to 1100 Da

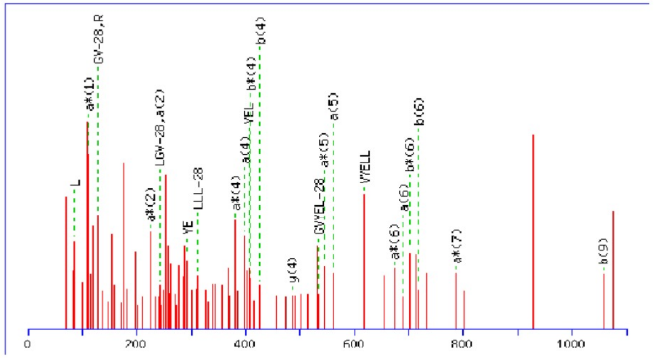

Monoisotopic mass of neutral peptide Mr(calc): 1202.74
Fixed modifications: Carbamidomethyl (C)
Ions Score: 17 **Expect:** 2.8
Matches (Bold Red): 30/132 fragment ions using 48 most intense peaks

#	Immon.	a	a*	a⁰	b	b*	b⁰	d	Seq.	y	y*	y⁰	#
1	**129.11**	**129.11**	**112.09**		157.11	140.08		44.05	R				10
2	**86.10**	**242.20**	**225.17**		270.19	253.17		200.15	L	1047.64	1030.62	1029.63	9
3	30.03	299.22	282.19		327.21	310.19			G	934.56	917.53	916.55	8
4	72.08	**398.29**	**381.26**		**426.28**	**409.26**		384.27	V	877.54	860.51	859.53	7
5	136.08	**561.35**	**544.32**		589.35	572.32			Y	778.47	761.44	760.46	6
6	102.05	**690.39**	**673.37**	672.38	**718.39**	**701.36**	700.38	632.39	E	615.41	598.38	597.40	5
7	**86.10**	803.48	**786.45**	785.47	831.47	814.45	813.46	761.43	L	**486.36**	469.34		4
8	**86.10**	916.56	899.53	898.55	944.56	927.53	926.55	874.51	L	373.28	356.25		3
9	**86.10**	1029.65	1012.62	1011.63	**1057.64**	1040.61	1039.63	987.60	L	260.20	243.17		2
10	101.11								K	147.11	130.09		1

MS/MS Fragmentation of **YIFHNFMER**
Found in **gi|31542984**, inter-alpha-trypsin inhibitor heavy chain H4 isoform 1 precursor [Homo sapiens]

Match to Query 26: 1271.574297 from(1272.581573,1+)
From data file DATA.TXT

Click mouse within plot area to zoom in by factor of two about that point
Or, Plot from 0 to 1300 Da

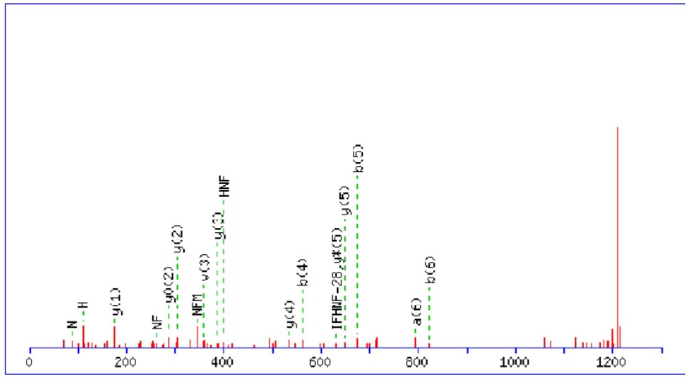

Monoisotopic mass of neutral peptide Mr(calc): 1271.58
Fixed modifications: Carbamidomethyl (C)
Variable modifications:
M7 : Oxidation (M)
Ions Score: 19 **Expect:** 3
Matches (Bold Red): 19/102 fragment ions using 42 most intense peaks

#	Immon.	a	a*	a⁰	b	b*	b⁰	Seq.	v	y	y*	y⁰	#
1	136.08	136.08			164.07			Y					9
2	86.10	249.16			277.15			I	987.44	1045.52	1028.49	1027.51	8
3	120.08	396.23			424.22			F	840.37	932.44	915.41	914.43	7
4	**110.07**	533.29			**561.28**			H	703.32	785.37	768.34	767.36	6
5	**87.06**	647.33	630.30		**675.32**	658.30		N	589.27	**648.31**	**631.28**	630.30	5
6	120.08	**794.40**	777.37		**822.39**	805.37		F	442.20	**534.27**	517.24	516.26	4
7	56.05	877.44	860.41		905.43	888.40		M	**359.17**	**387.20**	370.17	369.19	3
8	102.05	1006.48	989.45	988.47	1034.47	1017.45	1016.46	E	230.12	**304.16**	287.13	**286.15**	2
9	129.11							R	74.02	**175.12**	158.09		1

Spot C2 analyzed by MS/MS ion search

Supplementary Figure 4J

MS/MS Fragmentation of **MNFRPGVLSSR**
Found in **gi|31542984**, inter-alpha-trypsin inhibitor heavy chain H4 isoform 1 precursor [Homo sapiens]

Match to Query 27: 1278.647442 from(1279.654718,1+)
From data file DATA.TXT

Click mouse within plot area to zoom in by factor of two about that point
Or, Plot from 0 to 1300 Da

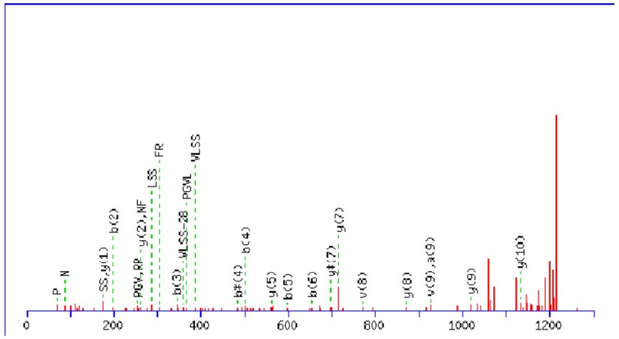

Monoisotopic mass of neutral peptide Mr(calc): 1278.65
Fixed modifications: Carbamidomethyl (C)
Variable modifications:
M1 : Oxidation (M)
Ions Score: 40 **Expect:** 0.032
Matches (Bold Red): 28/159 fragment ions using 49 most intense peaks

#	Immon.	a	a*	a^0	b	b*	b^0	d	Seq.	v	y	y*	y^0	#
1	56.05	56.05			84.04				M					11
2	**87.06**	170.09	153.07		**198.09**	181.06			N	1073.59	**1132.62**	1115.60	1114.61	10
3	120.08	317.16	300.13		**345.16**	328.13			F	**926.52**	**1018.58**	1001.55	1000.57	9
4	129.11	473.26	456.24		**501.26**	**484.23**		388.20	R	**770.42**	**871.51**	854.48	853.50	8
5	**70.07**	570.31	553.29		**598.31**	581.28		544.30	P	673.36	**715.41**	**698.38**	697.40	7
6	30.03	627.34	610.31		**655.33**	638.30			G		618.36	601.33	600.35	6
7	72.08	726.40	709.38		754.40	737.37		712.39	V	517.27	**561.34**	544.31	543.32	5
8	86.10	839.49	822.46		867.48	850.46		797.44	L	404.19	462.27	445.24	444.26	4
9	60.04	**926.52**	909.49	908.51	954.52	937.49	936.50	910.53	S	317.16	349.18	332.16	331.17	3
10	60.04	1013.55	996.53	995.54	1041.55	1024.52	1023.54	997.56	S	230.12	**262.15**	245.12	244.14	2
11	129.11								R	74.02	**175.12**	158.09		1

MS/MS Fragmentation of **LQDRGPDVLTATVSGK**
Found in **gi|31542984**, inter-alpha-trypsin inhibitor heavy chain H4 isoform 1 precursor [Homo sapiens]

Match to Query 35: 1655.875500 from(1656.882776,1+)
From data file DATA.TXT

Click mouse within plot area to zoom in by factor of two about that point
Or, Plot from 0 to 1700 Da

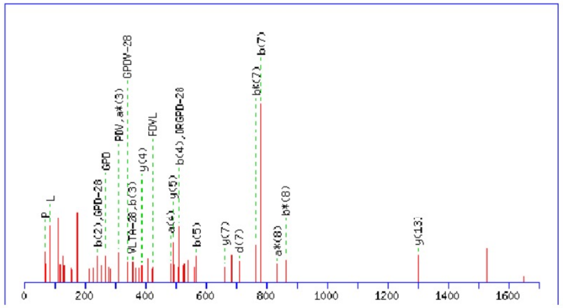

Monoisotopic mass of neutral peptide Mr(calc): 1655.88
Fixed modifications: Carbamidomethyl (C)
Ions Score: 29 **Expect:** 0.28
Matches (Bold Red): 26/276 fragment ions using 26 most intense peaks

#	Immon.	a	a*	a^0	b	b*	b^0	d	d'	Seq.	v	y	y*	y^0	#
1	**86.10**	**86.10**			114.09					L					16
2	101.07	214.16	197.13		**242.15**	225.12				Q	1470.75	1543.81	1526.78	1525.80	15
3	88.04	329.18	**312.16**	311.17	**357.18**	340.15	339.17			D	1355.73	1415.75	1398.72	1397.74	14
4	129.11	**485.28**	468.26	467.27	**513.28**	496.25	495.27	400.22		R	1199.63	**1300.72**	1283.70	1282.71	13
5	30.03	542.30	525.28	524.29	**570.30**	553.27	552.29			G		1144.62	1127.59	1126.61	12
6	**70.07**	639.36	622.33	621.35	667.35	650.33	649.34	613.34		P		1087.60	1070.57	1069.59	11
7	88.04	754.38	737.36	736.37	**782.38**	**765.35**	764.37	**710.39**		D		990.55	973.52	972.54	10
8	72.08	853.45	**836.43**	835.44	881.45	**864.42**	863.44	839.44		V		875.52	858.49	857.51	9
9	**86.10**	966.54	949.51	948.53	994.53	977.51	976.52	924.49		L		776.45	759.42	758.44	8
10	74.06	1067.58	1050.56	1049.57	1095.58	1078.55	1077.57	1051.59	1053.57	T		**663.37**	646.34	645.36	7
11	44.05	1138.62	1121.59	1120.61	1166.62	1149.59	1148.61			A		562.32	545.29	544.31	6
12	74.06	1239.67	1222.64	1221.66	1267.66	1250.64	1249.65	1223.67	1225.65	T		**491.28**	474.26	473.27	5
13	72.08	1338.74	1321.71	1320.73	1366.73	1349.71	1348.72	1324.72		V		**390.23**	373.21	372.22	4
14	60.04	1425.77	1408.74	1407.76	1453.76	1436.74	1435.75	1409.77		S		291.17	274.14	273.16	3
15	30.03	1482.79	1465.76	1464.78	1510.79	1493.76	1492.78			G		204.13	187.11		2
16	101.11									K		147.11	130.09		1

Spot C2 analyzed by MS/MS ion search

Supplementary Figure 4J

MS/MS Fragmentation of **SPEQQETVLDGNLIIR**
Found in **gi|31542984**, inter-alpha-trypsin inhibitor heavy chain H4 isoform 1 precursor [Homo sapiens]

Match to Query 37: 1810.927089 from(1811.934365,1+)
From data file DATA.TXT

Click mouse within plot area to zoom in by factor of two about that point
Or, Plot from 0 to 1700 Da

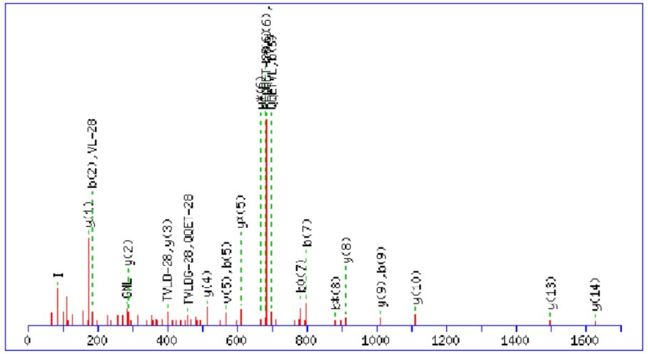

Monoisotopic mass of neutral peptide Mr(calc): 1810.94
Fixed modifications: Carbamidomethyl (C)
Ions Score: 101 **Expect:** 1.4e-008
Matches (Bold Red): 33/263 fragment ions using 23 most intense peaks

#	Immon.	a	a*	a^0	b	b*	b^0	Seq.	v	y	y*	y^0	#
1	60.04	60.04		42.03	88.04		70.03	S					16
2	70.07	157.10		139.09	**185.09**		167.08	P	1682.87	1724.92	1707.89	1706.91	15
3	102.05	286.14		268.13	314.13		296.12	E	1553.83	**1627.86**	1610.84	1609.85	14
4	101.07	414.20	397.17	396.19	442.19	425.17	424.18	Q	1425.77	**1498.82**	1481.80	1480.81	13
5	101.07	542.26	525.23	524.25	**570.25**	553.23	552.24	Q	1297.71	1370.76	1353.74	1352.75	12
6	102.05	671.30	654.27	653.29	**699.29**	**682.27**	681.28	E	1168.67	1242.71	1225.68	1224.69	11
7	74.06	772.35	755.32	754.34	**800.34**	783.32	**782.33**	T	1067.62	**1113.66**	1096.64	1095.65	10
8	72.08	871.42	854.39	853.41	899.41	**882.38**	881.40	V	968.55	**1012.61**	995.59	994.60	9
9	**86.10**	984.50	967.47	966.49	**1012.49**	995.47	994.48	L	855.47	**913.55**	896.52	895.54	8
10	88.04	1099.53	1082.50	1081.52	1127.52	1110.49	1109.51	D	740.44	800.46	783.44	782.45	7
11	30.03	1156.55	1139.52	1138.54	1184.54	1167.52	1166.53	G		**685.44**	**668.41**		6
12	87.06	1270.59	1253.56	1252.58	1298.59	1281.56	1280.58	N	**569.38**	628.41	**611.39**		5
13	**86.10**	1383.68	1366.65	1365.66	1411.67	1394.64	1393.66	L	456.29	**514.37**	497.34		4
14	**86.10**	1496.76	1479.73	1478.75	1524.75	1507.73	1506.74	I	343.21	**401.29**	384.26		3
15	**86.10**	1609.84	1592.82	1591.83	1637.84	1620.81	1619.83	I	230.12	**288.20**	271.18		2
16	129.11							R	74.02	**175.12**	158.09		1

MS/MS Fragmentation of **DQFNLIVFSTEATQWRPSLVPASAENVNK**
Found in **gi|31542984**, inter-alpha-trypsin inhibitor heavy chain H4 isoform 1 precursor [Homo sapiens]

Match to Query 38: 3260.634294 from(3261.641570,1+)
From data file DATA.TXT

Click mouse within plot area to zoom in by factor of two about that point
Or, Plot from 240 to 620 Da

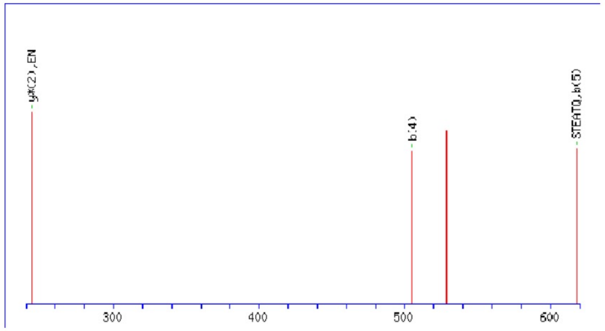

Monoisotopic mass of neutral peptide Mr(calc): 3260.65
Fixed modifications: Carbamidomethyl (C)
Ions Score: 13 **Expect:** 3
Matches (Bold Red): 6/541 fragment ions using 4 most intense peaks

#	Immon.	a	a*	a^0	b	b*	b^0	d	Seq.	v	y	y*	y^0	#
1	88.04	88.04		70.03	116.03		98.02		D					29
2	101.07	216.10	199.07	198.09	**244.09**	227.07	226.08		Q	3073.57	3146.63	3129.60	3128.62	28
3	120.08	363.17	346.14	345.16	391.16	374.13	373.15		F	2926.51	3018.57	3001.54	3000.56	27
4	87.06	477.21	460.18	459.20	**505.20**	488.18	487.19		N	2812.46	2871.50	2854.47	2853.49	26
5	86.10	590.29	573.27	572.28	**618.29**	601.26	600.28		L	2699.38	2757.46	2740.43	2739.45	25
6	86.10	703.38	686.35	685.37	731.37	714.35	713.36		I	2586.29	2644.37	2627.35	2626.36	24
7	72.08	802.45	785.42	784.44	830.44	813.41	812.43		V	2487.23	2531.29	2514.26	2513.28	23
8	120.08	949.51	932.49	931.50	977.51	960.48	959.50		F	2340.16	2432.22	2415.19	2414.21	22
9	60.04	1036.55	1019.52	1018.54	1064.54	1047.51	1046.53		S	2253.13	2285.15	2268.13	2267.14	21
10	74.06	1137.59	1120.57	1119.58	1165.59	1148.56	1147.58		T	2152.08	2198.12	2181.09	2180.11	20
11	102.05	1266.64	1249.61	1248.63	1294.63	1277.60	1276.62		E	2023.04	2097.07	2080.05	2079.06	19
12	44.05	1337.67	1320.65	1319.66	1365.67	1348.64	1347.66		A	1952.00	1968.03	1951.00	1950.02	18
13	74.06	1438.72	1421.69	1420.71	1466.72	1449.69	1448.71		T	1850.95	1896.99	1879.97	1878.98	17
14	101.07	1566.78	1549.75	1548.77	1594.77	1577.75	1576.76		Q	1722.89	1795.94	1778.92	1777.93	16
15	159.09	1752.86	1735.83	1734.85	1780.85	1763.83	1762.84		W	1536.81	1667.89	1650.86	1649.88	15
16	129.11	1908.96	1891.93	1890.95	1936.96	1919.93	1918.94	1823.90	R	1380.71	1481.81	1464.78	1463.80	14
17	70.07	2006.01	1988.99	1988.00	2034.01	2016.98	2016.00	1980.00	P		1325.71	1308.68	1307.70	13
18	60.04	2093.05	2076.02	2075.03	2121.04	2104.01	2103.03	2077.05	S		1228.65	1211.63	1210.64	12
19	86.10	2206.13	2189.10	2188.12	2234.12	2217.10	2216.11	2164.08	L		1141.62	1124.59	1123.61	11
20	72.08	2305.20	2288.17	2287.19	2333.19	2316.17	2315.18	2291.18	V		1028.54	1011.51	1010.53	10
21	70.07	2402.25	2385.22	2384.24	2430.25	2413.22	2412.23	2376.23	P		929.47	912.44	911.46	9
22	44.05	2473.29	2456.26	2455.28	2501.28	2484.26	2483.27		A		832.42	815.39	814.41	8
23	60.04	2560.32	2543.29	2542.31	2588.31	2571.29	2570.30	2544.32	S		761.38	744.35	743.37	7
24	44.05	2631.36	2614.33	2613.35	2659.35	2642.32	2641.34		A		674.35	657.32	656.34	6
25	102.05	2760.40	2743.37	2742.39	2788.39	2771.37	2770.38	2702.39	E		603.31	586.28	585.30	5
26	87.06	2874.44	2857.42	2856.43	2902.44	2885.41	2884.43	2831.44	N		474.27	457.24		4
27	72.08	2973.51	2956.48	2955.50	3001.51	2984.48	2983.49	2959.49	V		360.22	343.20		3
28	87.06	3087.55	3070.53	3069.54	3115.55	3098.52	3097.54	3044.55	N		261.16	**244.13**		2
29	101.11								K		147.11	130.09		1

Spot C2 analyzed by MS/MS ion search

Supplementary Figure 5

sample	age	age at onset	Duration of illness	Numano type	sex
1	23	19	4	2b	F
2	44	36	8	2a	F
3	21	21	0	5	F
4	53	35	18	1	F
5	55	35	20	2a	F
6	70	48	22	1	F
7	41	19	22	5	F
8	39	39	0	2b	F
9	42	23	19	5	F
10	36	35	1	2b	F
11	25	21	4	1	F
12	36	22	14	1	F
13	25	18	7	2a	F
14	68	25	43	4	F
15	51	10	41	5	F
16	57	11	46	5	F
17	42	19	23	5	F
18	18	16	2	1	F
19	43	20	23	2b	F
20	46	43	3	2b	F
21	57	36	21	1	F
22	33	21	12	5	F
mean	42	26	16		

The clinical characteristics of the patients for the ELISA assays (N=22).
